# Cartilage oligomeric matrix protein is an endogenous β-arrestin-2-selective allosteric modulator of AT1 receptor counteracting vascular injury

**DOI:** 10.1038/s41422-020-00464-8

**Published:** 2021-01-28

**Authors:** Yi Fu, Yaqian Huang, Zhao Yang, Yufei Chen, Jingang Zheng, Chenfeng Mao, Zhiqing Li, Zhixin Liu, Bing Yu, Tuoyi Li, Meili Wang, Chanjuan Xu, Yiwei Zhou, Guizhen Zhao, Yiting Jia, Wei Guo, Xin Jia, Tao Zhang, Li Li, Ziyi Liu, Shengchao Guo, Mingliang Ma, Heng Zhang, Bo Liu, Junbao Du, Wengong Wang, Chaoshu Tang, Pei Gao, Qingbo Xu, Xian Wang, Jianfeng Liu, Jinpeng Sun, Wei Kong

**Affiliations:** 1 Department of Physiology and Pathophysiology, School of Basic Medical Sciences, Peking University; Key Laboratory of Molecular Cardiovascular Science, Ministry of Education, Beijing, 100191 China; 2 Department of Pediatrics, Peking University First Hospital, Beijing, 100034 China; 3 Department of Biochemistry & Molecular Biology, School of Basic Medical Sciences, Shandong University, Jinan, Shandong 250012 China; 4 Department of Cardiology, China-Japan Friendship Hospital, Beijing, 100029 China; 5 College of Life Science and Technology, Collaborative Innovation Center for Brain Science, Huazhong University of Science and Technology; Key Laboratory of Molecular Biophysics, Ministry of Education, Wuhan, Hubei 430074 China; 6 Department of Vascular Surgery, Chinese PLA General Hospital, Beijing, 100853 China; 7 Department of Pathology, State Key Laboratory of Cardiovascular Disease, Fuwai Hospital, National Center for Cardiovascular Diseases, Chinese Academy of Medical Sciences and Peking Union Medical College, Beijing, 100037 China; 8 Department of Biochemistry and Molecular Biology, School of Basic Medical Sciences, Peking University Health Science Center, Beijing, 100191 China; 9 Department of Epidemiology and Biostatistics, School of Public Health, Peking University Health Science Center, Beijing, 100191 China; 10 Cardiovascular Division, The James Black Centre, King’s College London, London, SE5 9NU UK

**Keywords:** Extracellular signalling molecules, Mechanisms of disease

## Abstract

Compelling evidence has revealed that biased activation of G protein-coupled receptor (GPCR) signaling, including angiotensin II (AngII) receptor type 1 (AT1) signaling, plays pivotal roles in vascular homeostasis and injury, but whether a clinically relevant endogenous biased antagonism of AT1 signaling exists under physiological and pathophysiological conditions has not been clearly elucidated. Here, we show that an extracellular matrix protein, cartilage oligomeric matrix protein (COMP), acts as an endogenous allosteric biased modulator of the AT1 receptor and its deficiency is clinically associated with abdominal aortic aneurysm (AAA) development. COMP directly interacts with the extracellular N-terminus of the AT1 via its EGF domain and inhibits AT1-β-arrestin-2 signaling, but not Gq or Gi signaling, in a selective manner through allosteric regulation of AT1 intracellular conformational states. COMP deficiency results in activation of AT1a-β-arrestin-2 signaling and subsequent exclusive AAA formation in response to AngII infusion. AAAs in *COMP*^*–/–*^ or *ApoE*^*–/–*^ mice are rescued by AT1a or β-arrestin-2 deficiency, or the application of a peptidomimetic mimicking the AT1-binding motif of COMP. Explorations of the endogenous biased antagonism of AT1 receptor or other GPCRs may reveal novel therapeutic strategies for cardiovascular diseases.

## Introduction

The malfunction (overactivation) of the renin–angiotensin–aldosterone system (RAAS) plays an essential role in vascular pathogenesis, including atherosclerosis, hypertension, aortic aneurysm, etc.^1–3^ Angiotensin II (AngII), the primary mediator of the RAAS, exerts its diverse bioactive effects by activating the AT1 receptor (AngII type 1 receptor), a G protein-coupled receptor (GPCR).^4–7^ In addition to the classical view that GPCRs are working linearly, accumulating evidence has indicated that GPCRs attain distinct states of activation with “functional selectivity” for the downstream response; namely, individual pathways may be selectively activated or inactivated (biased agonism/antagonism).^8–11^ In particular, the physiological and pharmaceutical relevance of the “functional selectivity” of GPCRs was exemplified by recent studies examining AT1 receptor in which selective arrestin- and G protein-mediated downstream signaling pathways are activated by distinct physiological contexts, such as mechanical stretching.^12,13^ Additionally, the exploitation of arrestin-biased signaling has introduced new therapeutic potential to combat cardiovascular pathogenesis.^14–16^ Despite this significant progress, information about the inhibition of AT1-biased activation states by endogenous ligands, particularly how a change in this naturally occurring regulatory system correlates with disease progression, remains elusive. This question becomes immediately prominent and relevant because improperly increased AT1 activities are strongly correlated with a spectrum of vascular diseases, and thus a disruption of the endogenous antagonism of AT1 activity may also play critical causal roles in pathological processes.

Abdominal aortic aneurysm (AAA) is a life-threatening cardiovascular event with an extremely high mortality rate upon rupture.^17^ Current treatment options for AAA are limited to a recommendation of surgery for large AAAs, whereas no proven indication for pharmacological therapy is available for small-size AAAs or AAA expansion.^18,19^ Although the underlying mechanisms of AAA initiation and progression remain largely unknown, an AT1 receptor 1166 C polymorphism has been identified in patients with AAA^20^ and pharmacological blockade of the AT1 receptor by the sartan family of drugs prevents AAA in both AngII-dependent and -independent mouse models.^4,21,22^ Accordingly, a multicenter randomized trial of telmisartan is ongoing in patients with AAA.^23,24^ Although these recent findings indicated that AT1 receptor activity plays pivotal roles in AAA development, researchers have not determined whether the endogenous AT1 receptor-biased signaling system is involved in vascular homeostasis/dysfunction and how the disruption of this system contributes to the progression of AAA in the clinic. Here, by investigating clinical samples from patients with AAA, we determined that a decreased expression of cartilage oligomeric matrix protein (COMP), a matricellular glycoprotein, is strongly correlated with AAA development. According to further mechanistic studies, COMP serves as an endogenous selective inhibitor of AT1a-β-arrestin-2 signaling in mice by directly interacting with N-terminus of AT1a receptor through its epidermal growth factor (EGF) domain and allosterically regulating receptor conformations. The interaction between COMP and AT1a receptor plays a critical role in ameliorating AAA initiation and development in vivo, providing insights into an unnoticed, biased, endogenous regulatory mechanism of AT1 receptor as a modulator of vascular homeostasis and a therapeutic target for disease states. Our study not only helps elucidate the mechanisms regulating AT1 receptor activity during aortic aneurysm formation but may also provide opportunities to develop optimal medications for patients with AAA.

## Results

### Decreased COMP levels are associated with AAA in humans and mice

**Fig. 1:**
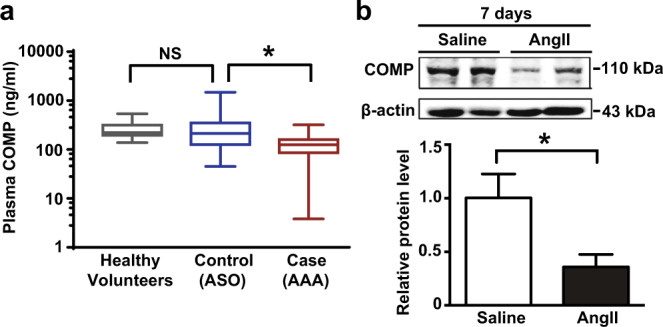
COMP is downregulated in both AAA patients and mice. **a** Plasma COMP levels measured by an ELISA in patients with AAA (Case, *n* = 88), patients with arteriosclerosis obliterans of the lower limbs without AAA (Control, *n* = 88) and healthy volunteers (*n* = 51). Data are presented as boxes (median with 1st quartile and 3rd quartile) and whiskers (minimum to maximum). *P < 0.05 by Wilcoxon signed-rank test; NS, no significance by Mann-Whitney test. **b** Representative western blot and quantitative analysis of the COMP protein level in the suprarenal aortas of *ApoE*^*–/–*^ mice infused with 1000 ng/kg/min AngII or saline for 7 days. *n* = 6 mice per group; *P < 0.05 by Mann-Whitney test.

As shown in our previous studies, COMP maintains vascular homeostasis and inhibits atherosclerosis, vascular calcification and thrombosis.^25–28^ We first designed a matched case-control study with matched gender frequency and comparable age distribution (Supplementary information, Table S1) in 88 cases (patients with AAA) and 88 controls (patients with arteriosclerosis obliterans of the lower limbs (ASO), but not AAA) to measure the plasma COMP levels and further investigated whether COMP is involved in the AAA etiology. The prevalence of diabetes in patients with AAA was significantly reduced (*P* < 0.001) in comparison with those ASO patients without AAA, consistent with previous reports,^29,30^ whereas other characteristics were not significantly different (Supplementary information, Table S1). Interestingly, substantially reduced plasma COMP levels were observed in patients with AAA (124.9 (88.0, 158.7) ng/mL, *n* = 88) compared with the control ASO group (212.6 (127.3, 351.0) ng/mL, *n* = 88, *P* < 0.0001), whereas the latter group exhibited a similar level to healthy volunteers (221.9 (197.1, 316.7) ng/mL, *n* = 51, *P* = 0.1712), indicating a substantial decrease in the plasma COMP level in patients with AAA compared to the normal plasma level (Fig. 1a). The odds ratios (95% CI) of AAA were estimated by the binary logistic regression model adjusted to the potential confounding factors including diabetes, age, hypertension, smoking and coronary heart disease. Odds ratios were calculated separately with the continuous plasma COMP levels or categorical tertiles in controls (< 213.8 ng/mL, 213.8–279.9 ng/mL and ≥ 279.9 ng/mL). The odds ratio for the risk of AAA was 44.123 (95% CI: 9.698–200.756) following a decrease in the plasma COMP levels by one unit of lg [COMP (ng/mL)] (Supplementary information, Table S2). Moreover, compared with the highest tertile of plasma COMP levels (≥ 279.9 ng/mL), the odds ratios for the risk of AAA for the middle (213.8–279.9 ng/mL) and lowest tertiles (< 213.8 ng/mL) were 57.954 (95% CI: 7.449–450.881) and 42.268 (95% CI: 4.354–410.303), respectively (Supplementary information, Table S2). Thus, plasma COMP levels may be negatively correlated with AAA. Next, we measured COMP expression in the suprarenal aortas of AngII-infused *ApoE*^*–/–*^ mice, a mouse model that displays multiple characteristics of human AAA.^31^ Reduced COMP levels were observed in the aortas of AngII-infused *ApoE*^*–/–*^ mice at an early stage (7 days) (Fig. 1b). Collectively, these data indicated that reduced COMP levels were strongly correlated with AAA in both humans and mice.

### COMP deficiency aggravates AngII-induced AAA formation in mice

**Fig. 2:**
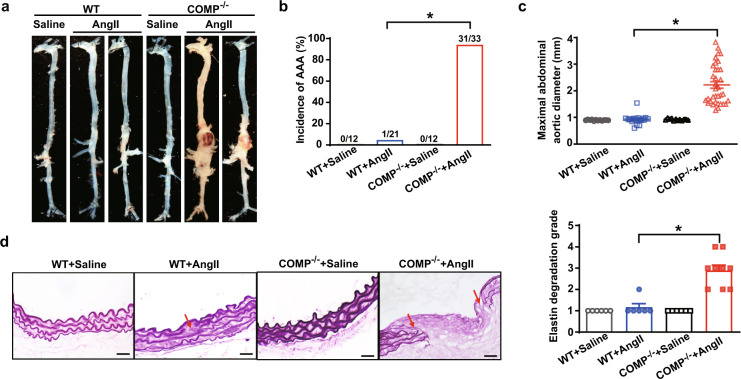
COMP deficiency aggravates AngII-induced AAA formation. **a** Representative images of the morphology of whole aortas from WT C57BL/6 J and *COMP*^*–/–*^ mice with or without 28 days of AngII infusion. **b** Incidence of AngII-induced AAA in WT (*n* = 21) and *COMP*^*–/–*^ mice (*n* = 33). *P < 0.05 by *χ*^2^ test. **c** The maximal abdominal aortic diameters (WT + Saline, *n* = 12; WT + AngII, *n* = 21; *COMP*^*–/–*^ + Saline, *n* = 12; *COMP*^*–/–*^ + AngII, *n* = 33). *P < 0.05 by Kruskal-Wallis test followed by Dunn’s test. **d** Representative images of Gomori staining and quantification of elastin degradation (WT + Saline, *n* = 6; WT + AngII, *n* = 6; *COMP*^*–/–*^ + Sali*n*e, *n* = 6; *COMP*^*–/–*^ + AngII, *n* = 9). *P < 0.05 by Kruskal-Wallis test followed by Dunn’s test. Scale bars, 50 μm.

We then used *COMP*^*–/–*^ mice to explore the role of COMP in AAA formation. AngII infusion significantly but comparably increased systolic blood pressure (SBP) in both *COMP*^*–/–*^ mice and wild-type (WT) littermates (C57BL/6 background, 5-month-old males) (Supplementary information, Table S3). WT mice infused with AngII exhibited a low incidence (~5%; 1/21) of AAA (Fig. 2a, b), consistent with a previous report.^32^ In contrast, *COMP*^*–/–*^ mice were highly susceptible to AngII induction of AAA. During the first 2 weeks of the experiment, ~11% (4/37, Supplementary information, Fig. S1) of the *COMP*^*–/–*^ mice died due to aortic dissection or aortic rupture (data not shown). At the end of 4 weeks, 31 of the 33 surviving mice exclusively suffered from suprarenal aortic aneurysms, i.e., nearly 94% (31/33) of mice developed AAA (Fig. 2a, b). Correspondingly, greater abdominal aortic diameters and elastin degradation were observed in *COMP*^*–/–*^ mice compared to WT mice upon AngII infusion (Fig. 2c, d). Thus, COMP protects against AngII-induced AAA in vivo.

We then assessed vascular inflammation, matrix metalloproteinase (MMP)-induced extracellular matrix degradation and oxidative stress in the suprarenal aortas, since these features are the major pathologies of AAA.^19,33^ Compared to WT mice, *COMP*^*–/–*^ mice exhibited greater inflammatory cell (CD45^+^ leukocytes, Mac-3^+^ macrophages, and CD4^+^ T cells) infiltration and increased MMP activity upon AngII infusion for 28 days (Supplementary information, Fig. S2a, b). As early as 7 days of the AngII infusion, suprarenal aortas of *COMP*^*–/–*^ mice released more MCP-1 and IL-6, exhibited greater MMP-9 activity and produced more reactive oxidative species (ROS) (Supplementary information, Fig. S2c–e), which have all been demonstrated to mediate AAA formation.^34–36^ Interestingly, even without AngII infusion, COMP deficiency alone markedly increased basal vascular wall inflammation, MMP activity, and oxidative stress, which may profoundly contribute to AngII-induced AAA formation (Supplementary information, Fig. S2b–e).

### Aortic COMP inhibits AngII-induced AAA formation in *ApoE*^*–/–*^ mice

AAA involves the inflammatory interaction between vascular cells and leukocytes. As COMP is expressed in both the vascular wall and leukocytes,^37^ we asked which origin of COMP contributes to the pathogenesis of AAA. First, WT mice received lethal radiation with ^60^Co followed by bone marrow transplantation from WT or *COMP*^*–/–*^ mice. The two types of chimeric mice infused with AngII displayed identical body weights and SBPs, as well as no AAA formation (0/10 vs 0/10) (Supplementary information, Fig. S3 and Table S4). Thus, the deficiency of aortic but not bone marrow-derived COMP might mainly contribute to AAA formation.

Next, we overexpressed COMP in the suprarenal aortas of *ApoE*^*–/–*^ mice to determine whether aorta-resident COMP was sufficient to prevent AAA formation, since previous reports indicated that 4–5-month-old *ApoE*^*–/–*^ mice exhibited a higher incidence of AAA (50%–70%) than C57BL/6 mice (10%).^38,39^ COMP was overexpressed in 4-month-old male *ApoE*^*–/–*^ mice via the periadventitial infection of an adenovirus encoding COMP (Ad-COMP) (Supplementary information, Fig. S4a, b). Following a 28-day AngII infusion, ~20% (3/15) of Ad-LacZ-infected *ApoE*^*–/–*^ mice died early (within two weeks) due to AAA rupture and 33% (5/15) of the remaining *ApoE*^*–/–*^ mice developed AAA, with a total AAA incidence of 53% (8/15). In contrast, only 7% (1/15) of Ad-COMP-infected *ApoE*^*–/–*^ mice developed AAA (Supplementary information, Fig. S4c–f and Table S5). Accordingly, AAA-related inflammation, MMP activity, and oxidative stress in the suprarenal aortas were markedly reduced in Ad-COMP-infected *ApoE*^*–/–*^ mice (Supplementary information, Fig. S4g–k). Similarly, we generated vascular smooth muscle cell (VSMC)-specific COMP transgenic (*COMP*^*SM-Tg*^) *ApoE*^*–/–*^ mice, as aortic COMP is mainly derived from VSMCs in the vessels.^37^ A 28-day AngII infusion caused > 50% (7/12) of the *ApoE*^*–/–*^ mice to develop AAA. In contrast, only 10% (1/10) of *ApoE*^*–/–*^*COMP*^*SM-Tg*^ mice exhibited AAA (Supplementary information, Fig. S5 and Table S6). Thus, aortic COMP prevents AngII-induced AAA formation in *ApoE*^*–/–*^ mice.

### Aortic COMP expression was downregulated by AngII through AUF-1-mediated mRNA decay

Next, we explored the mechanism of AngII-induced downregulation of aortic COMP during the pathogenesis of AAA (Fig. 1b). Since aortic COMP was mainly expressed in VSMCs,^25,37^ we firstly validated that AngII downregulated COMP expression in VSMCs at both mRNA and protein levels in a dose-dependent manner (Supplementary information, Fig. S6a, b). The results suggested that AngII may primarily regulate COMP mRNA level. We then used COMP promoter luciferase assay to examine the effect of AngII on gene transcription of COMP (Supplementary information, Fig. S6c). At the concentration of 1000 nM, AngII slightly inhibited luciferase transcription. Interestingly, 100 nM of AngII showed no significant effect on luciferase activity of COMP promoter, but still caused the decrease of COMP mRNA level. These results implied that AngII may affect COMP mRNA stability. Indeed, AngII (100 nM) significantly enhanced COMP mRNA decay following actinomycin D treatment in VSMCs (Supplementary information, Fig. S6d). HuR (also named as ELAVL1, embryonic lethal abnormal vision like RNA binding protein 1), an RNA stabilizer and AUF-1 (AU-rich binding protein 1), an RNA destabilizer are two main regulators of mRNA stability.^40^ We found that AngII mainly upregulated AUF-1, without affecting HuR expression in VSMCs (Supplementary information, Fig. S6e). In accordance, the aortic AUF-1 expression was also upregulated in vivo following 7 days of AngII infusion (Supplementary information, Fig. S6f). Moreover, silencing of AUF-1 by siRNA rescued AngII-induced COMP mRNA decay and downregulation of COMP expression (Supplementary information, Fig. S6g–j). Conversely, overexpression of AUF-1 decreased COMP expression, as well as destabilized COMP mRNA (Supplementary information, Fig. S6k–m). In addition, RNA immunoprecipitation (RIP) assay demonstrated that AUF-1 directly interacted with COMP mRNA (Supplementary information, Fig. S6n). Collectively, aortic COMP expression was downregulated by AngII mainly through AUF-1-mediated destabilization of COMP mRNA.

### AT1a deficiency rescues AAA formation in *COMP*^*–/–*^ mice in vivo

**Fig. 3:**
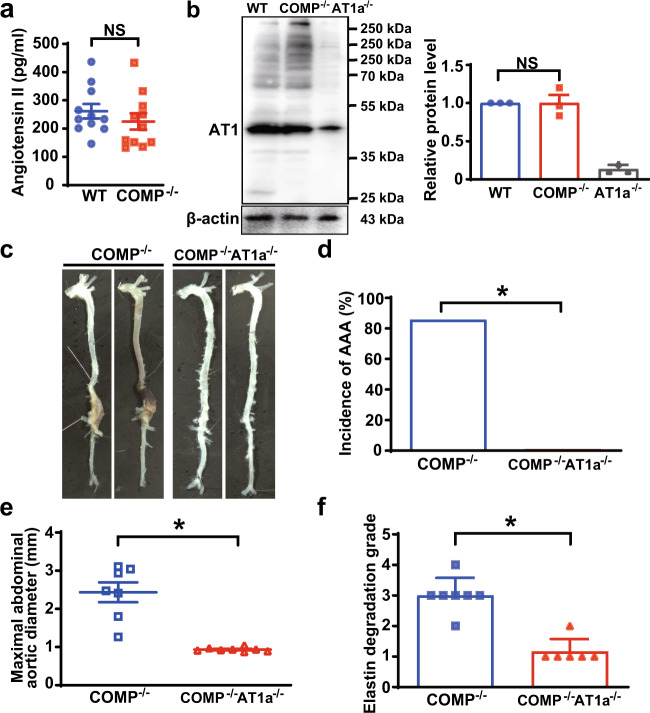
AT1a receptor mediates AAA formation in *COMP*^*–/–*^ mice. **a** AngII concentrations in aortic explants from 5-month-old WT and *COMP*^*–/–*^ mice. *n* = 11 animals per group; NS, no significance by Mann-Whitney test. **b** Western blot analysis of AT1 expression in the aortas of WT, *COMP*^*–/–*^ and *AT1a*^*–/–*^ mice. *n* = 3 mice per group. NS, no significance by One-way ANOVA followed by the Bonferroni test. **c** Representative images of the aortic morphology in *COMP*^*–/–*^ or *COMP*^*–/–*^*AT1a*^*–/–*^ mice infused with AngII (1000 ng/kg/min) for 28 days. **d** Incidence of AngII-induced AAA (*COMP*^*–/–*^: 6/7; *COMP*^*–/–*^*AT1a*^*–/–*^: 0/8). *P < 0.05 by *χ*^2^ test. **e** The maximal abdominal aortic diameters. *COMP*^*–/–*^, *n* = 7; *COMP*^*–/–*^*AT1a*^*–/–*^, *n* = 8. *P < 0.05 by Mann-Whitney test. **f** Quantification of elastin degradation. *COMP*^*–/–*^, *n* = 7; *COMP*^*–/–*^*AT1a*^*–/–*^, *n* = 6. *P < 0.05 by Mann-Whitney test.

Mouse AT1 receptors comprise two subtypes with 94% amino acid identity, AT1a and AT1b receptors. Interestingly, AT1b receptor is associated with AngII-induced vascular contractility, but not with AngII-induced aortic pathologies.^41^ AngII-induced AAA formation primarily depends on AT1a receptor-mediated vascular oxidative stress, inflammation, and MMP activation or matrix degradation in mice.^42^ AT1a deletion or inhibition abolishes AngII-induced AAA formation in mice.^43^ Therefore, we speculated that COMP deficiency may increase vascular AngII levels, upregulate AT1a receptor expression, or induce AngII downstream signaling activation. Similar levels of AngII were secreted from aortic explants of 5-month-old WT and *COMP*^*–/–*^ mice (Fig. 3a). We next examined the level of the AT1 receptor in WT and *COMP*^*–/–*^ aortas. We used a commercially available AT1 antibody (Cat# 25343-1-AP; ProteinTech) and characterized the specificity of the antibody by performing western blot of lysates from HEK293A cells overexpressing Flag-tagged mouse AT1a, AT1b and AT2 as well as human AT1 receptors, respectively. Consequently, the antibody successfully recognized human AT1, mouse AT1a and AT1b receptors, but not AT2 receptor (Supplementary information, Fig. S7). Using this antibody, AT1 expression was observed in WT aortas, but significantly decreased in *AT1a*^*–/–*^ aortas (Fig. 3b). The expression of AT1b in *AT1a*^*–/–*^ aortic tissues may account for the residual bands observed in *AT1a*^*–/–*^ aortas. Interestingly, COMP deficiency did not affect levels of the AT1 protein in aortas (Fig. 3b). Meanwhile, real-time PCR analysis verified that the mRNA level of AT1a receptor was not altered in the suprarenal aortas by COMP deficiency, either (Supplementary information, Fig. S8a). Therefore, we hypothesized that COMP deficiency may affect AngII-activated downstream signaling in vessels. The mitogen-activated protein kinase (MAPK) and Smad pathways have been reported to be downstream of the AT1a receptor^2^ and involved in aortic aneurysm formation.^44–46^ Thus, we analyzed whether COMP affected AT1 signaling in vivo. COMP deficiency alone activated extracellular signal-regulated kinase 1/2 (ERK1/2) and p38-MAPK, but not JNK-MAPK or Smad2/3, in suprarenal aortas from mice infused with saline, similar to WT mice infused with AngII. These signals were further amplified in the suprarenal aortas of 7-day AngII-infused *COMP*^*–/–*^ mice compared to tissues from WT mice (Supplementary information, Fig. S8b). It implied that COMP deficiency amplifies AngII-activated ERK1/2 and p38-MAPK pathways in vessels, which may be connected to AT1a receptor and mediate AAA formation.

To further confirm whether the pathogenic effects of COMP deficiency were mediated by AngII production or AngII receptors in vivo, 5-month-old *COMP*^*–/–*^ mice were administered with drinking water containing the AT1 receptor blocker losartan (30 mg/kg/day) or the ACE inhibitor enalapril (15 mg/kg/day) for 7 consecutive days, without AngII minipump infusion, and the suprarenal aortas were excised to measure IL-6 and MMP levels or vascular AngII production in vivo. As expected, enalapril significantly decreased the aortic AngII production (Supplementary information, Fig. S9a). Intriguingly, losartan but not enalapril reduced IL-6 expression and secretion as well as MMP-9 expression (Supplementary information, Fig. S9b–d). Previous study demonstrated that the inhibition of vascular pathologies by losartan was partially attributable to the AT2 signaling activation.^47^ We therefore additionally administered the AT2 blocker PD123319 (30 mg/kg/day) in drinking water to *COMP*^*–/–*^ mice. As expected, AT2 inhibition by PD123319 markedly aggravated the vascular pathologies in *COMP*^*–/–*^ suprarenal aortas. In the presence of PD123319, losartan still exhibited the inhibitive effects on IL-6 production and MMP-9 expression in *COMP*^*–/–*^ mice (Supplementary information, Fig. S9b–d). Taken together, AT1 receptor activation, but not the AngII level or total AT1 expression, mediates the dysfunction of *COMP*^*–/–*^ suprarenal aortas.

We next verified whether COMP deficiency led to AAA formation via the AT1a receptor in vivo using *COMP*^*–/–*^ mice and *COMP*^*–/–*^*AT1a*^*–/–*^ mice with AngII infusion for 28 days. As expected, blood pressure was significantly decreased in AngII-infused *COMP*^*–/–*^*AT1a*^*–/–*^ mice compared to *COMP*^*–/–*^ mice (Supplementary information, Table S7). Approximately 90% of the *COMP*^*–/–*^ mice developed AAA (6/7), whereas none of the *COMP*^*–/–*^*AT1a*^*–/–*^ mice exhibited AAA (Fig. 3c, d). Accordingly, the expansion of abdominal aortas and degradation of elastin were substantially reversed by AT1a knockout in *COMP*^*–/–*^ mice (Fig. 3e, f). Thus, COMP deficiency induces AAA formation via a mechanism that involves the activation of AT1a receptor.

### COMP directly binds to the N-terminus of the AT1 receptor

**Fig. 4:**
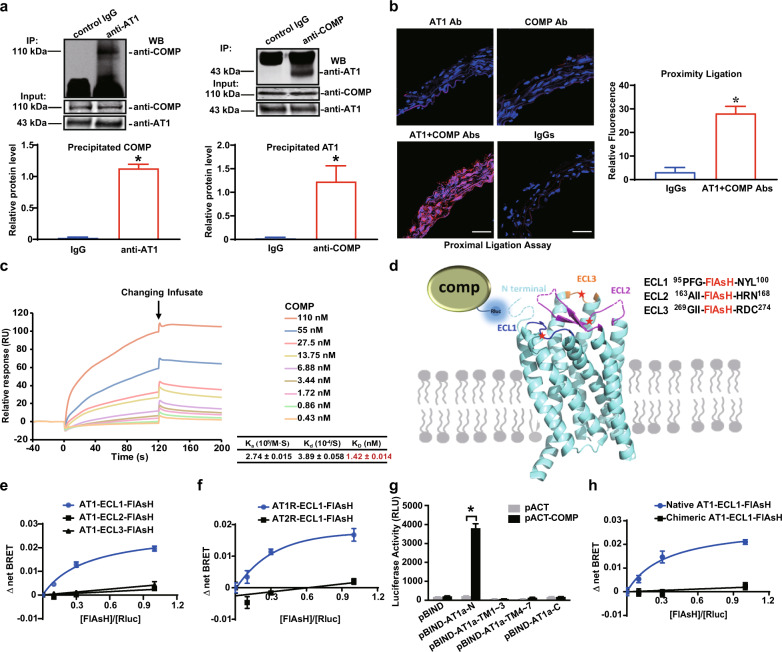
COMP directly binds to the AT1 receptor. **a** Co-IP assay with suprarenal aortas from C57BL/6 J mice. Left panel, vascular extracts were incubated with an anti-AT1 antibody or control IgG, followed by Protein A/G agarose beads. The COMP protein was examined using a western blot analysis. Right panel, vascular extracts were immunoprecipitated with an anti-COMP antibody or control IgG. The AT1 protein was then examined using a western blot analysis. Input was evaluated using aortic lysates before immunoprecipitation. *n* = 3; *P < 0.05 by the unpaired Student’s *t*-test. **b** Proximity ligation assay of AT1 receptor and COMP using specific antibodies on cross-sections of suprarenal aortas from 5-month-old male mice. *n* = 3; *P < 0.05 by the unpaired Student’s *t*-test. Scale bars, 50 μm. **c** SPR sensorgrams of the binding of an increasing amount of COMP to AT1 receptor captured on a CM5 chip. The increase in RUs from baseline was measured and used to calculate the binding kinetics, including association constant (*K*_a_), dissociation constant (*K*_d_) and affinity constant (*K*_D_) for COMP binding to immobilized AT1 receptor. **d** Structural representation of the AT1 receptor with a TC-tag (CCPGCC) inserted at corresponding sites in ECL1, ECL2 or ECL3 as AT1-FlAsH plasmids, which were further separately co-transfected with COMP-RLuc into HEK293T cells. **e** Saturation BRET signal between RLuc and FlAsH in HEK293T cells co-transfected with a fixed amount of COMP-RLuc plasmid and an increasing amount of AT1-ECL-FlAsH plasmids (*n* = 3). **f** Saturation BRET signal between RLuc and FlAsH in HEK293T cells co-transfected with a fixed amount of COMP-RLuc plasmid and an increasing amount of AT1-ECL1-FlAsH or AT2-ECL1-FlAsH plasmids (*n* = 3). **g** Mammalian two-hybrid analysis of the AT1–COMP interaction. COS-7 cells were transiently transfected with various domain constructs of AT1a receptor in the pBIND vector, together with COMP fused into the pACT vector. Cells were lysed after 48 h, and the luciferase activity was determined. The data are presented as the means ± SEM of 3 independent experiments performed in triplicate. *P < 0.05 by Two-way ANOVA followed by the Bonferroni test. **h** Saturation BRET signal between RLuc and FlAsH in HEK293T cells co-transfected with a fixed amount of COMP-RLuc plasmid and an increasing amount of AT1-ECL1-FlAsH or a chimeric AT1-ECL1-FlAsH with the alternative N-terminus from AT2 receptor (*n* = 3).

We next examined the molecular mechanism by which the matrix protein COMP interferes with the function of membrane-bound AT1a receptor during AAA formation. We asked whether COMP directly binds to the AT1 receptor. Proteins from suprarenal aortas of C57BL/6 J mice were subjected to co-immunoprecipitation (co-IP) with specific AT1/COMP antibodies or control IgG to address this hypothesis. A specific COMP band was present in the complex immunoprecipitated by the anti-AT1 antibody but not by the control IgG. Reciprocally, COMP co-immunoprecipitated with AT1 receptor (Fig. 4a). Meanwhile, COMP and AT1 receptor did not reciprocally co-immunoprecipitate with each other in *AT1a*^*–/–*^ aortic samples, indicating that COMP did not interact with AT1b in mice (Supplementary information, Fig. S10a). Furthermore, a proximity ligation assay using anti-AT1 and anti-COMP antibodies revealed an interaction between endogenous AT1 receptor and COMP in the aortic wall of C57BL/6 J mice, whereas rabbit and goat IgGs were employed to exclude the nonspecific binding of antibodies (Fig. 4b). Moreover, the co-IP assay identified a similar specific interaction between COMP and AT1a receptor in Flag-AT1a-overexpressing COS-7 cells that were incubated with the purified COMP protein for 30 min (Supplementary information, Fig. S10b).

To further validate the direct interaction between COMP and AT1 receptor, we performed surface plasmon resonance (SPR) by using AT1 receptor and COMP protein purified from baculovirus-infected insect cells (Supplementary information, Fig. S10c). Through the gel filtration chromatography and non-reduced native PAGE followed by western blotting, we verified that the purified COMP protein existed in a pentameric form with a molecular weight of 524 kDa (Supplementary information, Fig. S10d, e). For SPR assay, we applied a CM5 sensor chip immobilized with purified AT1 receptor, and the activity of immobilized receptor was validated by AngII infusion. As shown in Supplementary information, Fig. S10f, AngII bound to AT1 receptor with a *K*_D_ of 0.78 ± 0.39 nM. Then, an increasing amount (0.43–110 nM) of pentameric COMP protein was infused over the CM5 sensor chip immobilized with purified AT1 receptor. A dose-dependent increase of SPR signaling following infusion was observed, while reduced response, demonstrating the dissociation process, existed once injection was ceased, indicating the direct binding of COMP to AT1 receptor (Fig. 4c). The measured *K*_D_ value of COMP–AT1 interaction is 1.42 ± 0.014 nM, indicating a potential high-affinity binding between COMP and AT1 receptor. In contrast, infusion of the thrombospondin-1 (TSP-1), another member belonging to the thrombospondin family encompassing COMP, caused no significant SPR response (Supplementary information, Fig. S10g). Thus, COMP specifically binds to AT1.

Next, we used the intermolecular FlAsH-bioluminescence resonance energy transfer (FlAsH-BRET) assay to assess the interaction mode between AT1 receptor and COMP. This method has been successfully used to examine the interaction modes between β2-adrenergic receptor and arrestins or arrestin and downstream effectors by our previous study.^48^ COMP was tagged with the modified Renilla luciferase (RLuc) moiety at the C-terminus, without disrupting its pentameric form (Supplementary information, Fig. S11a), whereas the FlAsH motif (tetracysteine (TC)-tag, CCPGCC) was introduced into the 1st, 2nd or 3rd extracellular loop (ECL1, ECL2 or ECL3) of AT1 receptor, respectively (Fig. 4d). We firstly controlled the equal expression levels of different AT1 constructs on cell surface (Supplementary information, Fig. S11b). Then, we evaluated whether the insertions of FlAsH motifs into extracellular loops affect the AT1 downstream signaling. The AT1 receptor mainly signals through 2 types of pathways: (1) the canonical G protein signals and (2) β-arrestin-mediated functions, such as assembling receptor with downstream protein kinases together to modulate phosphorylation networks.^7,49^ In general, the dissociation of Gα and Gβγ directly indicates the activation of GPCR-coupled G proteins, whereas the interaction of β-arrestins and the AT1 receptor is crucial for β-arrestin-mediated sustained activation of AT1 signaling.^50–52^ Therefore, we measured AngII-induced Gq dissociation from Gβγ, β-arrestin-2 recruitment and the phosphorylation of protein kinase C (PKC) and ERK1/2 under the similar receptor expression, and confirmed that these insertions did not affect either G protein- or β-arrestin-related signaling (Supplementary information, Fig. S11c–f). We next confirmed that the fluorescent labeling efficiencies of different AT1-ECL-FlAsH constructs were comparable when expressed at similar levels on cell surface (Supplementary information, Fig. S11g). To ensure the functional responsiveness of the FlAsH fluorescence acceptor, we inserted the RLuc moiety at the N-terminus of three AT1-ECL-FlAsH constructs, respectively, based on the rationale that AngII-induced activation of AT1 would cause a conformational change of ECLs that could be revealed by intramolecular FlAsH-BRET assay.^10^ Our results indicated that AngII stimulation induced significant BRET signal between the N-terminal RLuc and the FlAsH acceptor at all three ECLs, which could be blocked by pre-incubation with losartan, confirming the functionality of the AT1-ECL-FlAsH constructs (Supplementary information, Fig. S11h).

The FlAsH-BRET assay was then applied. The specific saturation FlAsH-BRET signal, which indicated a constitutive interaction, was only detected between COMP-RLuc and the AT1-ECL1-FlAsH, but not the AT1-ECL2-FlAsH or AT1-ECL3-FlAsH mutants (Fig. 4e). To further verify the specificity of COMP–AT1 interaction, we constructed AT2 receptor with TC-tag inserted into ECL1, which does not impair its Gi activation under the equal surface expression compared to native AT2 receptor (Supplementary information, Fig. S11i, j). Distinct from AT1-ECL1 mutant, no constitutive BRET signal was observed between AT2-ECL1-FlAsH and COMP-RLuc (Fig. 4f). These data suggest that COMP specifically interacts with AT1 rather than AT2 receptor, in an orientation by placing the C-terminus of COMP in proximity to the ECL1 of AT1 receptor.

We then subcloned different structural domains of AT1a (N-terminus, C-terminus, and transmembrane part separated by intracellular loop 2), and characterized which part of the receptor is responsible for the COMP interaction by performing a mammalian two-hybrid dual-luciferase reporter assay. The N-terminal domain of AT1a, but not other domains, bound to COMP (Fig. 4g). To further verify the interaction between COMP and AT1 N-terminus, we replaced the N-terminus of AT1-ECL1-FlAsH with that of AT2 receptor, which shares 38% similarity in amino acid sequence with AT1 receptor. The chimeric construct showed similar Gq-activating capacity compared to native AT1-ECL1 mutant when expressed at similar levels on cell surface (Supplementary information, Fig. 11i, k). Consequently, no significant BRET signal was observed between chimeric AT1-ECL1-FlAsH and COMP-RLuc (Fig. 4h). Thus, COMP is a novel extracellular protein that specifically binds to the N-terminus of the AT1 receptor.

### COMP selectively antagonizes β-arrestin, but not G protein signaling of AT1 receptor

**Fig. 5:**
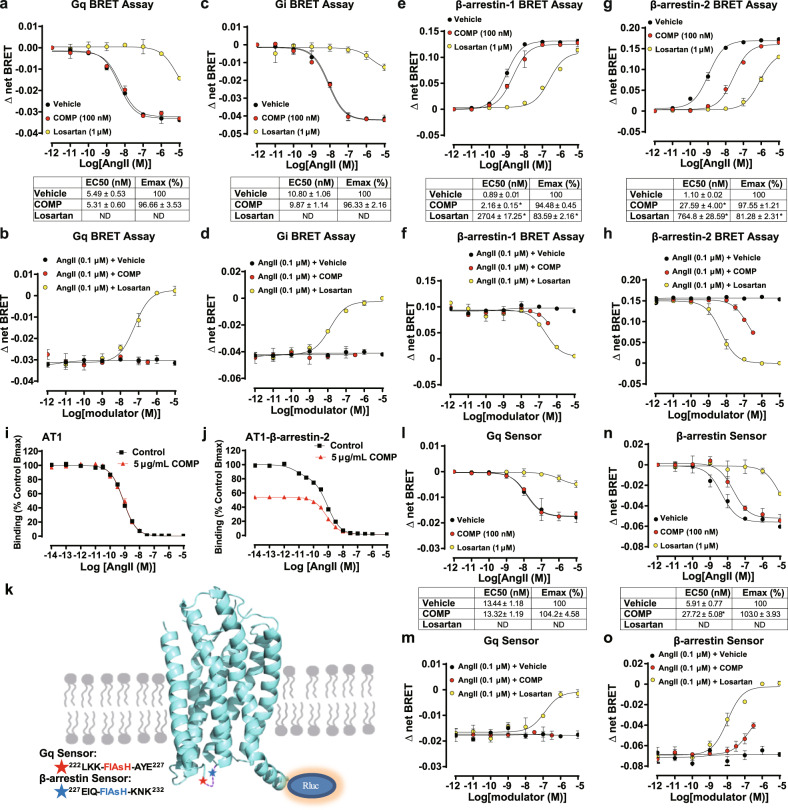
COMP selectively inhibits the β-arrestin, but not G protein, in AT1 signaling. **a–d** The effects of COMP on AngII-induced Gq and Gi activation through AT1. HEK293T cells were co-transfected with Gq BRET probes (Gq-RLucII, Gβ1, Gγ1-GFP10) (**a**, **b**) and Gi BRET probes (Gi1-RLuc8, Gβ3, Gγ9-GFP10) (**c**, **d**), respectively. The transfected cells were pre-incubated with purified COMP (100 nM) or losartan (1 μM), followed by stimulation with an increasing amount of AngII for 2 min (**a**, **c**), or with various concentrations of COMP or losartan, followed by stimulation with AngII (0.1 μM) for 2 min (**b**, **d**). The BRET signal was measured and presented as the ratio of the emission of GFP10 to RLucII (**a**, **b**) or RLuc8 (**c**, **d**). The data are presented as the means ± SEM from 3 independent experiments. One-way ANOVA followed by the Bonferroni test, *P < 0.05 vs vehicle. **e–h** The effects of COMP on AngII-induced β-arrestin-1 and β-arrestin-2 recruitment through AT1. HEK293T cells were co-transfected with the AT1-YFP and β-arrestin-1-RLuc plasmids (**e**, **f**) or the AT1-YFP and β-arrestin-2-RLuc plasmids (**g**, **h**), respectively. The transfected cells were pre-incubated with purified COMP (100 nM) or losartan (1 μM) for 30 min and then stimulated with an increasing amount of AngII for 5 min (**e**, **g**), or with various concentrations of COMP or losartan for 30 min and then stimulated with the AngII (0.1 μM) for 5 min (**f**, **h**). The BRET signal was measured and presented as the ratio of the emission of YFP to RLuc. The data are presented as the means ± SEM from 3 independent experiments performed in triplicate. One-way ANOVA followed by the Bonferroni test, *P < 0.05 vs vehicle. **i**, **j** The specific bind**i**ng of [^125^I]-AngII to WT AT1 receptor (**i**) or AT1–β-arrestin-2 fusion (**j**) with an increasing amount of unlabeled AngII in the absence or presence of purified COMP (5 μg/mL). **k** Schematics illustrating the AT1 receptor with a RLuc moiety fused to the C-terminus as well as a TC-tag (CCPGCC) inserted between K224 and A225 or Q229 and K230 in the ICL3 as a Gq- or β-arrestin-specific conformational sensor, respectively. **l**, **m** The effects of COMP on AngII-induced conformational change of AT1 Gq-specific sensor. HEK293T cells transfected with the Gq-specific conformational sensor of AT1 receptor were preincubated with purified COMP (100 nM) or losartan (1 μM) for 30 min followed by stimulation with an increasing amount of AngII for 2 min (**l**), or preincubated with various concentrations of COMP or losartan for 30 min followed by AngII (0.1 μM) stimulation for 2 min (**m**). *n* = 6; One-way ANOVA followed by the Bonferroni test. **n**, **o** The effects of COMP on AngII-induced conformational change of AT1 β-arrestin-specific sensor. HEK293T cells transfected with the β-arrestin-specific conformational sensor of AT1 receptor were preincubated with purified COMP (100 nM) or losartan (1 μM) for 30 min followed by stimulation with an increasing amount of AngII for 5 min (**n**), or preincubated with various concentrations of COMP or losartan for 30 min followed by AngII (0.1 μM) stimulation for 5 min (**o**). *n* = 6; One-way ANOVA followed by the Bonferroni test, *P < 0.05 vs vehicle.

We next studied whether aortic COMP interfered with AT1 downstream signaling through a G protein-dependent or β-arrestin-mediated pathway or both. For G protein-related signaling, AT1 receptor has been reported to couple with Gq and Gi.^53^ Therefore, we tested the effects of COMP on both signaling pathways in AT1-overexpressing HEK293T cells by BRET assay. Administration of AngII significantly induced dissociation of both Gαq and Gαi from their corresponding Gβγ in a dose-dependent manner, as evidenced by the decrease of BRET signals (Fig. 5a, c). These dissociations were dose-dependently inhibited by the AT1 antagonist losartan but not by COMP, even with an increasing concentration up to 300 nM (Fig. 5a–d). In accordance, the addition of COMP displayed no significant effect on the Gq downstream signaling, including intracellular calcium mobilization and PKC phosphorylation (Supplementary information, Fig. S12). Therefore, COMP had little effect on G protein-mediated signaling of AT1 receptor induced by AngII.

Two isoforms of β-arrestin, β-arrestin-1 and -2, are involved in AT1 signal transduction. Although β-arrestin-1 has been reported to be much more abundant than β-arrestin-2 in cardiac tissue,^54^ these two isoforms are comparably expressed in suprarenal aortic tissue in mice, as shown by real-time PCR analysis (Supplementary information, Fig. S13a). Herein, we next explored the potential effects of COMP on both β-arrestin-1- and β-arrestin-2-related AT1 signaling. The BRET assay in HEK293T cells revealed that COMP (100 nM) significantly inhibited AngII-induced recruitment of both β-arrestin-1 and β-arrestin-2 to AT1 receptor (Fig. 5e, EC_50_: Vehicle 0.89 ± 0.01 nM vs COMP 2.16 ± 0.15 nM; Fig. 5g, EC_50_: Vehicle 1.10 ± 0.02 nM vs COMP 27.56 ± 4.00 nM). Meanwhile, the dose-dependent inhibitory effects of COMP on both β-arrestin-1 and β-arrestin-2 recruitment were observed (Fig. 5f, h). In contrast, COMP did not influence the isoprenaline-induced recruitment of β-arrestin-2 to the β2-adrenergic receptor (Supplementary information, Fig. S13b). Notably, AT1 expressions on cell surface in both G protein activation and β-arrestin recruitment assays were comparable (Supplementary information, Fig. S13c), excluding the possibility that the distinct effects of COMP on selective AT1 signal pathways were due to the difference of the receptor expression levels. Taken together, COMP is a selective inhibitor of AT1 receptor, by selectively inhibiting β-arrestin recruitment to AT1 receptor, but not affecting G protein-related AT1 signaling. Since COMP inhibits both β-arrestin-mediated AT1 signaling, we then explored whether β-arrestin-1 or β-arrestin-2 contributed to AngII-induced vascular pathologies through ex vivo culture of aortic rings from *β-arrestin-1*^*–/–*^ and *β-arrestin-2*^*–/–*^ mice, respectively. As a result, the deficiency of β-arrestin-2, rather than β-arrestin-1, abolished AngII-induced MCP-1 and IL-6 production in suprarenal aortic rings (Supplementary information, Fig. S13d). This data was consistent with previous studies,^55–58^ which showed that β-arrestin-2, but not β-arrestin-1, mediates AngII-induced ERK1/2 activation and both thoracic aortic aneurysm and AAA formation. In accordance, we further demonstrated that COMP inhibited the phosphorylation of ERK1/2 induced by 15-min stimulation with AngII (Supplementary information, Fig. S13e), which was attributable to β-arrestin-2-related signaling.^59^ Thus, we mainly focused on the effect of COMP on β-arrestin-2-related AT1 signaling in the pathological process of AAA.

To investigate the molecular basis of COMP as a selective inhibitor, we conducted competitive radio-ligand binding assay using HEK293T cells overexpressing the unfused AT1 or AT1 receptor with β-arrestin-2 fusion to its C-terminus. The GPCR-transducer fusion proteins serve as an effective approach to quantify the biased signaling and elucidate the molecular basis of selective property, as evaluated in both AT1 and VLGR1 receptors in previous studies.^60,61^ As shown in Fig. 5i, AngII dose-dependently inhibited [^125^I]-AngII binding to the unfused AT1 receptor (*K*_D_ = 0.79 nM, 95% CI: 0.72–0.87 nM), whereas the addition of COMP exerted no effect on the competitive feature (*K*_D_ = 0.82 nM, 95% CI: 0.68–0.99 nM), indicating that COMP may not interfere with the orthosteric site of AT1 receptor. We next used the AT1–β-arrestin-2 fusion plasmid to dissect the effect of the AngII binding on high-affinity state of the AT1–β-arrestin-2 complex. Similar to the previous report,^60^ the competitive curve of unlabeled AngII to displace [^125^I]-AngII on AT1 fused with β-arrestin-2 fitted into a two-site binding model (low affinity: *K*_Lo_ = 0.88 nM, 95% CI: 0.67–1.15 nM; high affinity: *K*_Hi_ = 6.78 pM, 95% CI: 1.41–32.57 pM), compared to the curve for the unfused AT1, indicating two affinity states of AT1, the uncoupled (low affinity, equal to the unfused AT1) and the β-arrestin-2-coupled (high affinity) receptors (Fig. 5j). Interestingly, the presence of COMP altered the competitive binding curve which fitted preferentially to the one-site binding model (*K*_D_ = 0.75 nM, 95% CI: 0.69–0.82 nM, close to the *K*_Lo_ for low affinity or the *K*_D_ for unfused/uncoupled AT1 receptor). Moreover, the maximal [^125^I]-AngII bound to β-arrestin-2-fused AT1 receptor was markedly decreased by > 40% in the presence of COMP. Thus, these results indicated that COMP allosterically regulated the high-affinity conformational state of AT1–β-arrestin-2 complex.

To further define how COMP regulated conformational states of AT1 receptor and explore the potential structural mechanism by which COMP selectively regulated AT1 signaling, we performed the intramolecular FlAsH-BRET assay using our recently developed two AT1 receptor-biased conformational sensors (incorporation of FlAsH into the 3rd intracellular loop (ICL3) at K224–A225 to generate a Gq signaling-specific conformational sensor and incorporation of FlAsH at Q229–K230 to generate a β-arrestin signaling-specific conformational sensor) (Fig. 5k; Supplementary information, Fig. S14a, b). This intramolecular FlAsH-BRET assay has been successfully applied to characterize the conformational changes occurring during GPCR signaling, as well as conformational states of AT1 in previous studies.^4,55,56,62^ Here, AT1 receptor with insertion of the FlAsH motif did not significantly impair Gq signaling or β-arrestin recruitment, compared to the similar expression amount of native AT1 (AT1-WT) or AT1 tagged with the RLuc moiety at the C-terminus (Supplementary information, Fig. S14c–e), suggesting that the mutants did not disrupt the structural integrity of the AT1 receptor. COMP exhibited no significant effect on the conformational change in the Gq sensor in response to AngII, which dose-dependently increased the distance between the ICL3 and C-terminus as revealed by BRET signals (Fig. 5l, m); in contrast, COMP significantly blocked the conformational change in the β-arrestin sensor in response to AngII (EC_50_, Vehicle 5.97 ± 0.77 nM vs COMP 27.72 ± 5.08 nM). Moreover, the dose-dependent inhibitory effect of COMP on β-arrestin-specific conformational change of AT1 was observed (Fig. 5n, o). These data suggests that a specific conformational modulation is correlated with the function of COMP as an endogenous β-arrestin-selective antagonist.

Moreover, the interaction of β-arrestin and the AT1 receptor is followed by receptor internalization and desensitization, which is critical for further signal transduction.^50–52^ Specifically, we monitored receptor internalization by confocal microscopy and flow cytometry analysis in AT1-GFP-overexpressing HeLa cells and HEK293A cells transfected with Flag-AT1 plasmid, respectively (Supplementary information, Fig. S15a, b). Consequently, AngII caused AT1 internalization, which was reversed by addition of purified COMP protein. Meanwhile, the desensitization of AT1 receptor, indicated by a transient AngII-induced phosphorylation of PKC, was markedly abolished by COMP, as evidenced by a long-term activation of PKC by AngII in the presence of COMP (Supplementary information, Fig. S15c).

### β-Arrestin-2 mediates AAA formation in *COMP*^*–/–*^ mice

**Fig. 6:**
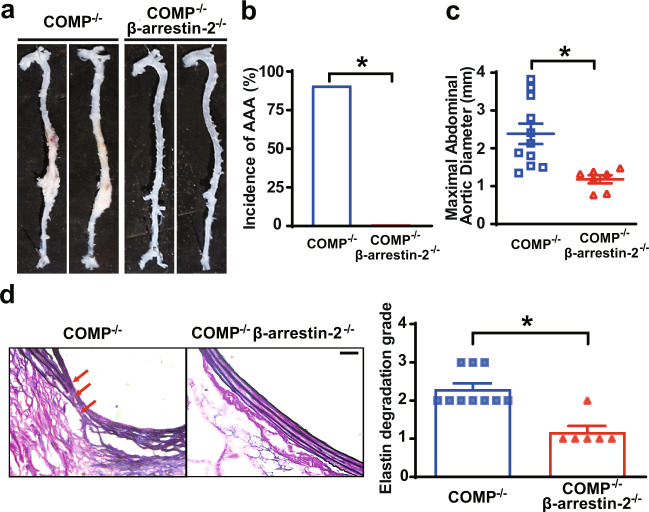
β-Arrestin-2 mediates the pathological activation of the AT1 receptor aggravated by COMP deficiency. **a** Representative images of the aortic morphology in *COMP*^*–/–*^ or *COMP*^*–/–*^*β-arrestin-2*^*–/–*^ mice infused with AngII (1000 ng/kg/min) for 28 days. **b** Incidence of AngII-induced AAA (*COMP*^*–/–*^: 10/11; *COMP*^*–/–*^*β-arrestin-2*^*–/–*^: 0/7). *P < 0.05 by *χ*^2^ test. **c** The maximal abdominal aortic diameters. *COMP*^*–/–*^ + AngII, *n* = 11; *COMP*^*–/–*^*β-arrestin-2*^*–/–*^ + AngII, *n* = 7. *P < 0.05 by Mann-Whitney test. **d** Representative images of VVG staining and quantification of elastin degradation. *COMP*^*–/–*^ + AngII, *n* = 10; *COMP*^*–/–*^*β-arrestin-2*^*–/–*^ + AngII, *n* = 6. *P < 0.05 by Mann-Whitney test. Scale bar, 50 μm.

We next crossed *COMP*^*–/–*^ mice with *β-arrestin-2*^*–/–*^ mice to compare AngII-induced AAA formation between *COMP*^*–/–*^ and *COMP*^*–/–*^*β-arrestin-2*^*–/–*^ mice and further validate whether β-arrestin-2 mediates the effect of COMP deficiency on AAA formation in vivo. Significant differences in body weights, SBPs, total cholesterol and triglyceride levels, and fasting blood glucose were not observed between these two mouse genotypes (Supplementary information, Table S8). Greater than 90% of the *COMP*^*–/–*^ mice developed AAA (10/11), whereas none of the *COMP*^*–/–*^*β-arrestin-2*^*–/–*^ mice exhibited AAA (0/7) (Fig. 6a, b). Consistent with these findings, the maximal abdominal aortic diameter and elastin degradation were substantially decreased in *COMP*^*–/–*^*β-arrestin-2*^*–/–*^ mice compared to *COMP*^*–/–*^ mice (Fig. 6c, d). Thus, AT1a-β-arrestin-2 signaling mediates the COMP deficiency-aggravated AngII-induced AAA formation in mice.

### The peptidomimetic of COMP EGF2 domain directly binds to AT1 and ameliorates AAA formation

**Fig. 7:**
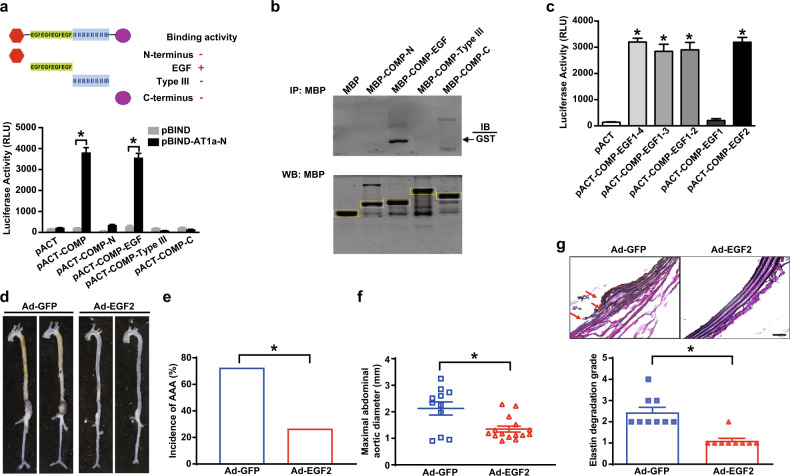
The COMP EGF2 domain ameliorates AAA formation. **a** Upper panel, schematics illustrating the COMP constructs used to map the corresponding domains (N-terminus, EGF, type III, and C-terminus) that bind to the AT1a N-terminus. Presence or absence of binding between COMP domains and the AT1a N-terminus is indicated by + or –, respectively. Lower panel, mammalian two-hybrid analysis of the interaction between COMP and the AT1a N-terminus. COS-7 cells were transiently transfected with various domains of COMP cloned into pACT, together with the AT1a N-terminus cloned into pBIND. Cells were lysed after 48 h, and the luciferase activity was determined. The data are presented as the means ± SEM of 3 independent experiments performed in triplicate. *P < 0.05 by Two-way ANOVA followed by the Bonferroni test. **b** Upper panel, purified MBP-fusion proteins of the COMP N-terminus (MBP-COMP-N), EGF domain (MBP-COMP-EGF), Type III repeat (MBP-COMP-Type III) and C-terminus (MBP-COMP-C) were immobilized on amylose resin and incubated with the purified GST-fused N-terminus of AT1a. Trapped proteins were examined by immunoblotting with an anti-GST antibody. Purified MBP (first lane) was used as a control. Lower panel, western blot analysis of MBP-COMP-N, MBP-COMP-EGF, MBP-COMP-Type III and MBP-COMP-C. **c** Mammalian two-hybrid assay of the AT1a N-terminus with various EGF domains of COMP. The data are presented as the means ± SEM of 3 independent experiments performed in triplicate. One-way ANOVA followed by the Bonferroni test, *P < 0.05 vs pACT. **d** Representative images of the aortic morphology in 5-month-old male *ApoE*^*–/–*^ mice periadventitially infected with Ad-GFP (Control) or Ad-EGF2 (COMP-EGF2) followed by 28 days of AngII infusion. **e** Incidence of AngII-induced AAA in *ApoE*^*–/–*^ mice periadventitially infected with Ad-GFP (8/11) or Ad-EGF2 (4/15). *P < 0.05 by *χ*^2^ test. **f** The maximal abdominal aortic diameters of *ApoE*^*–/–*^ mice periadventitially infected with Ad-GFP or Ad-EGF2. *P < 0.05 by Mann-Whitney test. **g** Representative images of VVG staining and quantification of elastin degradation. *n* = 9; *P < 0.05 by Mann-Whitney test. Scale bar, 50 μm.

Next, we identified the specific domain of COMP that directly bound to AT1 receptor. Various domains of COMP (N-terminus, EGF repeats, type III repeats and C-terminus) were subcloned into the pACT plasmid to generate the pACT-COMP-N, pACT-COMP-EGF, pACT-COMP-Type III, and pACT-COMP-C constructs, respectively, which were separately co-transfected into COS-7 cells with the pBIND vector harboring the N-terminal domain of AT1a receptor. A luciferase assay revealed that only the EGF domain of COMP bound to the extracellular N-terminus of AT1a receptor (Fig. 7a). Accordingly, only the maltose-binding protein (MBP)-fused EGF domain of COMP pulled down the glutathione S-transferase (GST)-fused N-terminus of AT1a receptor (Fig. 7b). The mouse COMP EGF domain includes 4 repeats: EGF1 (aa 85–124), EGF2 (aa 125–177), EGF3 (aa 178–220), and EGF4 (aa 221–265). Deletion of EGF2–4 (EGF1, aa 85–124), but not EGF3–4 (EGF1–2, aa 85–177) and EGF4 (EGF1–3, aa 85–220), disrupted the interaction between the COMP EGF domain and AT1a N-terminus, indicating that EGF2 may play a critical role in the COMP–AT1a interaction. Indeed, the EGF2 domain directly interacted with the AT1a N-terminus (Fig. 7c). Thus, COMP bound to the N-terminus of AT1a receptor through its EGF2 motif.

To further validate the possible function of the peptidomimetic of EGF2 domain, we created an adenovirus encoding COMP-EGF2 domain fused with a Flag tag at N-terminus, as shown by western blot to overexpress a secretable Flag-tagged EGF2 peptide, while the EGF1 domain, which does not bind to AT1a (Fig. 7c), was applied to package a control virus encoding a secretory EGF1 (Supplementary information, Fig. S16a). We firstly evaluated the effect of adenovirus infection on AngII-induced aortic pathologies in aortic rings ex vivo. As shown in Supplementary information, Fig. S16b, c, overexpression of EGF2, but not EGF1, markedly inhibited AngII-induced aortic inflammation. Of interest, overexpression of the secretory EGF1 exhibited no effect on AngII-induced inflammation, compared to Ad-GFP infection, indicating that the secretion process would not influence the pathogenic effects of AngII on aortas. Thus, Ad-COMP-EGF2 or Ad-GFP mixed with pluronic gel was then applied periadventitially to the suprarenal aortas of *ApoE*^*–/–*^ mice 3 days before the AngII infusion, succeeding to EGF2 overexpression for further binding to aortic AT1 receptor (Supplementary information, Fig. S16d–f). Upon AngII stimulation, ~18.2% (2/11) of Ad-GFP-infected *ApoE*^*–/–*^ mice died early (within two weeks) due to AAA rupture and 66.7% (6/9) of the remaining *ApoE*^*–/–*^ mice developed AAA at 28 days, with a total AAA incidence of 72.7% (8/11), whereas only 26.7% (4/15) of Ad-COMP-EGF2-infected *ApoE*^*–/–*^ mice exhibited AAA (Fig. 7d, e). Of note, the blood pressure was similar within two groups (Supplementary information, Table S9). Consistent with these findings, the maximal abdominal aortic diameter and elastin degradation were reduced in Ad-COMP-EGF2- compared to Ad-GFP-infected *ApoE*^*–/–*^ mice (Fig. 7f, g). Therefore, the EGF2 peptidomimetic of COMP directly binds to AT1a receptor and inhibits AAA formation in vivo.

## Discussion

Aberrant activation of the AT1 receptor is a pivotal causal factor and hallmark of the development of many cardiovascular diseases. Moreover, at least two different biased signals, Gq and β-arrestin pathways, have recently been shown to mediate distinct AT1 functions.^10,49,57,58^ These observations led to two tightly associated and important unanswered questions regarding the role of AT1 signaling complexes in vascular homeostasis and disease states, which are: (1) how is endogenous AT1-biased signaling fine-tuned in healthy vessels to maintain cardiovascular homeostasis, and (2) what is the mechanism underlying the dysregulation of AT1-biased signaling associated with vascular pathogenesis, such as AAA formation?

**Fig. 8:**
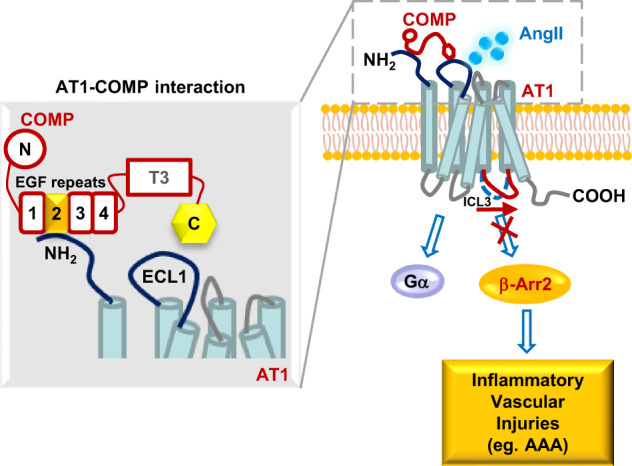
Schematic diagram of selective allosteric inhibition of AT1 signaling by COMP in pathogenesis of AAA. COMP, as an endogenous selective allosteric inhibitor of AT1 receptor, inhibits AngII-induced AT1-β-arrestin-2 signaling, but not G protein (Gq or Gi) signaling, thereby counteracting inflammatory vascular injuries. COMP directly binds to N-terminus of AT1 through its EGF2 domain, in a form that the C-terminus of COMP was placed in proximity to ECL1 of the AT1 receptor. β-Arr2, β-arrestin-2; ECL1, extracellular loop 1; ICL3, intracellular loop 3. COMP molecule: N, N-terminus; T3, type 3 repeats; C, C-terminus. AAA, abdominal aortic aneurysm.

In the present study, a decrease in COMP expression dysregulated AT1a signaling during the onset of the disease. Our mechanistic investigations revealed that COMP is an endogenous selective allosteric inhibitor of AT1a-β-arrestin-2 signaling in vessels (Fig. 8). COMP selectively inhibited AT1a-β-arrestin-2 activation but allowed normal AT1a-Gq or -Gi signaling, maintaining proper AT1a function, such as contraction and blood pressure maintenance in vessels. However, the loss of COMP expression was responsible for the increased activation of the AT1a-β-arrestin-2 pathway, ultimately leading to AAA formation and progression. These findings were supported by our clinical observations, signaling and functional studies using *ApoE*^*–/–*^, *COMP*^*–/–*^, *AT1a*^*–/–*^ and *β-arrestin-2*^*–/–*^ mice, the biophysical studies with intramolecular FlAsH-BRET sensors, and the competitive radio-ligand binding assay using both low-affinity uncoupled and high-affinity β-arrestin-2-coupled AT1 receptors. These results are not only consistent with recently published data showing reduced AAA formation in *β-arrestin-2*^*–/–*^ mice upon AngII stimulation,^63^ but also delineate a landscape in depth with its clinical relevance, in vivo regulatory mechanism, detailed structural basis and pharmacological characterization of biased AT1 signaling regulated by COMP, an endogenous biased modulator. Of interest, the selective antagonistic effect of COMP on AT1-β-arrestin-2 signaling is Ca^2+^ sensitive, as evidenced by the observation that Ca^2+^ depletion by EGTA completely abrogated the inhibitory effect of COMP on AngII-induced β-arrestin-2 recruitment (Supplementary information, Fig. S17). Notably, angiotensin-1–7 and mechanical stretch selectively activate endogenous β-arrestin-dependent AT1 signaling.^12,57^ However, the clinical significance of these endogenous regulatory systems remains unknown and both stimuli are endogenous agonists, leaving the endogenous selective antagonism of AT1 signaling as an open question. Here, through the identification of COMP as an endogenous selective inhibitor of AT1-β-arrestin-2 signaling, we presented an example of how endogenous biased signaling of GPCR family members is delicately regulated in physiological contexts and its relevance in preventing disease onset, such as AAA development. Of interest, β-arrestin-biased agonists were proposed to be utilized in the therapy of heart failure due to its cardioprotective effects,^7^ indicating that the same GPCR-biased signaling may play distinct roles in different tissues or organs. Thus, therapeutic application of EGF2 peptidomimetic or compound derived from interaction motifs between AT1 and COMP to selectively inhibit AT1-β-arrestin-2 signaling in AAA may require additional concerns of the potential detrimental effects in other tissues or organs, such as heart.

Another interesting finding of our study is that COMP regulated AT1 function in an allosteric manner. Our SPR assay revealed the direct binding of COMP to AT1 receptor with a *K*_D_ of 1.42 ± 0.014 nM, indicating a relatively high affinity. Further biochemical mapping of this interaction suggested that COMP interacted with N-terminus of AT1 receptor, a position that is apart from the classic AT1 orthosteric ligand-binding site.^64,65^ This biochemical characterization is consistent with the competitive radio-ligand binding data, as COMP did not significantly affect the AngII binding affinity toward WT AT1 receptor alone. Moreover, our intermolecular BRET experiments indicated that COMP bound to N-terminus of AT1 but not AT2 receptor, in a form that the C-terminus of COMP was placed in proximity to ECL1 of the AT1 receptor, also as shown in the proposed working model in Fig. 8. Notably, one of the advantages of allosteric regulation is its exquisite subtype selectivity and maintenance of spatiotemporal control over orthosteric signaling. Although the in vivo spatiotemporal regulation of AT1a signaling by COMP mainly depends on the expression pattern of COMP in different tissues or cell subtypes in different physiological processes, COMP showed substantial receptor selectivity, as it did not bind to or affect signaling mediated by other receptors such as mouse AT1b (data not shown) or AT2 receptor, which respectively share 94% or 38% amino acid identity with AT1a, and β2-adrenergic receptor-β-arrestin-2 signaling was not affected by COMP, either (Supplementary information, Fig. S13b). The delicate allosteric regulation of AT1 activation by COMP was directly strengthened by evidence showing that COMP only blocked the conformational change specific to arrestin signaling, but not G protein signaling. In particular, our competitive radio-ligand binding assay further indicated that COMP allosterically regulated the binding of AngII to the high-affinity state of the AT1–β-arrestin-2 complex, as evidenced by the elimination of the high-affinity state but keeping the low-affinity binding state alone. Moreover, mimicking the allosteric interaction of COMP–AT1a by overexpressing the peptidomimetic of EGF2 domain (53 amino acids) showed therapeutic potential for AAA treatment, suggesting that an allosteric antagonism of AT1a signaling may represent a potential new therapeutic strategy for the treatment of cardiovascular diseases, such as AAA.

Interestingly, although a previous study identified that the vessel wall-resident but not bone marrow-derived AT1a receptor mediates AAA formation,^43^ recent data from cell-specific AT1a-knockout mice suggest that AT1a deficiency in vascular endothelial cells, VSMCs or adventitial fibroblasts alone does not affect AngII-induced aortic pathologies, such as AAA, atherosclerosis and medial hyperplasia of descending aortas.^66,67^ These findings indicate a potential synergistic effect between different vascular cell types during overactivation of AT1a receptor signaling in disease states. As COMP is mainly a secreted matricellular protein, COMP may exert a profound effect on multiple cell types rather than one cell type involved in the vessel wall via direct interactions and allosteric regulation of the AT1a receptor. In conclusion, our study not only revealed a novel mechanism for counteracting AT1 receptor overactivation during physiological homeostasis but also disclosed the disease-relevant activation of AT1a receptor aggravated by COMP deficiency. We extended the repertoire of endogenous GPCR regulatory systems by identifying that allosteric antagonism of AT1-biased signaling by COMP maintains vascular homeostasis and inhibits AngII-induced AAA formation in vivo. Given the large number of GPCR family members, the biased regulation of GPCR functions by endogenous selective allosteric inhibitors is likely a previously unappreciated but important common mechanism for many GPCR systems, highlighting the function of fine-tuned GPCR signaling in different physiological and pathological contexts and providing a potential new avenue for future drug discoveries.

## Materials and Methods

### Materials

AngII, losartan, enalapril and the antibody against Flag were purchased from Sigma-Aldrich (St. Louis, MO, USA). PD123319 was obtained from Selleckchem (Houston, TX, USA). Antibodies against COMP (Cat# ab42225, for western blot) and total p38-MAPK were obtained from Abcam (Cambridge, UK). The antibody against β-actin was purchased from Santa Cruz Biotechnology (Santa Cruz, CA, USA). Antibodies against β-arrestin-2, p-p38, phosphorylated and total ERK (p-ERK and t-ERK), p- and t-Smad2/3, p- and t-JNK, p- and t-pan-PKC and AUF-1 were obtained from Cell Signaling Technology (Boston, MA, USA). The antibody against AT1 (Cat# 25343-1-AP) was obtained from Proteintech Group, Inc. (Rosemont, IL, USA).

### Human study population

A matched case-control study was conducted in China between July 2011 and December 2012. Patients (*n* = 88) who were diagnosed with AAA at the Vascular and Endovascular Surgery Department of the Chinese PLA General Hospital in Beijing were enrolled. Patients with AAA must have been diagnosed with infrarenal AAAs using abdominal Doppler ultrasound or computed tomography (CT). The control participants (*n* = 88) who were selected from the Vascular and Endovascular Surgery ward at the Chinese PLA General Hospital were diagnosed with arteriosclerosis obliterans of the lower limbs, but not AAA. The patients were excluded if 1) they had been diagnosed with inflammatory AAA, which has a different etiology; 2) they were experiencing serious, active, inflammatory processes that could lead to hyperpyrexia, which might influence the predictive value of the examined biochemical parameters; or 3) they had any mental disorders or were pregnant. AAA and control groups were matched by gender frequency and age distribution in both genders. Moreover, blood samples from 51 healthy volunteers were collected to assess the normal plasma COMP levels.

The study was conducted in accordance with the ethical guidelines of the 1975 Declaration of Helsinki, and the study protocol was approved by the Scientific and Ethics Committee of the Chinese PLA General Hospital. Informed consent was obtained from all study participants. All experiments were performed in accordance with relevant guidelines and regulations.

### Data collection procedures and statistical analysis of data from the human study

A standardized questionnaire was used to collect data related to the subjects’ demographic characteristics, history of chronic diseases, chronic medications, and lifestyle factors. The participants underwent a standard physical examination. AAA was defined as a maximal infrarenal aortic diameter ≥ 30 mm. The participants in the control group underwent abdominal ultrasonographic examinations by two trained specialists to exclude subjects with AAA. All blood samples were centrifuged to separate serum or plasma within 1 h of collection and stored at –80 °C until analysis. Biochemical parameters, including blood glucose and lipid levels, were analyzed at the clinical laboratory of the Chinese PLA General Hospital. Plasma COMP levels were evaluated using a human COMP Quantikine ELISA kit from R&D Systems (Cat# DCMP0, Minneapolis, MN, USA). A detailed description of the assessment of major environmental risk factors was reported in our previous study.^68^ Subjects were considered smokers if they had smoked > 100 cigarettes over their lifetimes. Drinking was defined as consuming at least 50 mL of white spirits at a time and having a drink at least once a week for a period of > 6 months. Self-reported chronic diseases included type 2 diabetes and coronary artery disease (CAD). Hypertension was defined either as a SBP ≥ 140 mmHg, a diastolic blood pressure (DBP) ≥ 90 mmHg, or the use of hypotensive drugs.

Data are presented as the means ± standard deviations (SD) or medians (quartile 1, quartile 3) for normally or non-normally distributed continuous variables, and as frequencies or percentages for categorical variables. The unpaired Student’s *t*-test or Wilcoxon signed-rank test was applied to evaluate the statistical significance continuous variables with or without a normal distribution, respectively. Meanwhile, the *χ*^2^ test was used to determine any statistical significance between the proportions of the two groups. As the plasma COMP levels demonstrated a skewed distribution, log transformation was applied to fit a more normal distribution. Then, binary logistic regression was used to evaluate the associations between lg [COMP (ng/mL)] and AAA. Multivariate adjusted models (variables adjusted for diabetes, age, hypertension, smoking and CAD) were applied to evaluate the risk of AAA for both continuous and tertile categorical plasma COMP levels. A two-sided probability level of 0.05 was used to evaluate statistical significance. All analyses were performed with the statistical software SPSS.

### Animal preparation

All animal studies adhered to the guidelines of the Animal Care and Use Committee of Peking University. The *COMP*^*–/–*^ mice on a C57BL/6 J background were kindly provided by Professor Oldberg Ake from the Department of Cell and Molecular Biology at Lund University, Sweden.^69^ The *COMP*^*SM-Tg*^ mice on a C57BL/6 J background were created by excising the sequence of the sm22 promoter linked with the mouse COMP cDNA sequence from the vector prior to pronuclear injection into fertilized C57BL/6 J mouse oocytes. The *COMP*^*SM-Tg*^ mice were crossed with *ApoE*^*–/–*^ mice to produce *ApoE*^*–/–*^*COMP*^*SM-Tg*^ mice for further studies.^37^ The *AT1a*^*–/–*^ mice were purchased from the Jackson Laboratory (Sacramento, CA, USA). The *β-arrestin-1*^*–/–*^ and *β-arrestin-2*^*–/–*^ mice on a C57BL/6 J background were kindly provided by Prof. Robert J. Lefkowitz from Duke University and Prof. Gang Pei from Tongji University as previously described.^70^ The *COMP*^*–/–*^*AT1a*^*–/–*^ mice and *COMP*^*–/–*^*β-arrestin-2*^*–/–*^ mice were produced by crossbreeding *COMP*^*–/–*^ mice with *AT1a*^*–/–*^ mice and *β-arrestin-2*^*–/–*^ mice, respectively. Five-month-old male *COMP*^*–/–*^, *COMP*^*–/–*^*AT1a*^*–/–*^, *COMP*^*–/–*^*β-arrestin-2*^*–/–*^ or WT mice and 4-month-old male *ApoE*^*–/–*^ or *ApoE*^*–/–*^*COMP*^*SM-Tg*^ mice fed a normal chow diet were infused with 1000 ng/kg/min AngII or saline for 7 or 28 days.^31^

The SBP was monitored using tail-cuff plethysmography. A noninvasive computerized tail-cuff system (CODA High Throughput System, Kent Scientific) was used to measure the SBP of mice.^71,72^ The equipment was cleaned to ensure that it was free from foreign scents and blood odors. The investigator was blinded to the experimental groups when performing the measurements, and mice were tested in a randomized fashion. Mice underwent 7 consecutive days of training sessions from 1 to 5 PM each day to become accustomed to the tail-cuff procedure. Measurements were then recorded by a single investigator at the same time on 3 consecutive days. Five measurements were recorded from each mouse daily, and the average of 15 measurements was reported as the SBP of each mouse. Then, the mice were sacrificed, blood samples were obtained, and the abdominal aortas were excised for further experiments.

### Immunohistochemical staining for COMP expression in the aortic wall

Paraffin sections of frozen sections of mouse suprarenal aortas were incubated with 3% hydrogen peroxide, followed by blocking with 3% normal blocking serum. Sections were incubated with a primary antibody against COMP (1:200, Cat# MABT36, Millipore, Danvers, MA, USA) overnight at 4 °C, followed by incubation with an HRP-labeled anti-rat IgG secondary antibody before staining with a DAB Kit (ZSGB-BIO, Beijing, China). Nuclei were counterstained with hematoxylin. Sections incubated with rat IgG alone were used as negative controls.

### Western blot analysis

Cell and tissue extracts containing equal amounts of total proteins were resolved on 10% or 15% SDS-PAGE gels for western blot analyses. For measuring the pentameric form of COMP, 6% native PAGE gels were applied in a procedure without using SDS and β-mecaptoethanol in the buffer system. Blots were incubated with the primary antibody and an IRDye 700DX-conjugated secondary antibody (Rockland Inc., Gilbertsville, PA, USA). The immunofluorescent signal was detected and quantified using an Odyssey infrared imaging system (LI-COR Biosciences, Lincoln, NB, USA).

### Analysis of plasma lipid

Plasma samples were collected from AngII- or saline-infused mice. Total plasma triglyceride and cholesterol levels were assessed with kits from Zhong Sheng Bio-technology (Beijing, China).

### Measurement of blood glucose

The blood glucose levels were monitored from tail vein blood of 5-month-old male *COMP*^*–/–*^ and *COMP*^*–/–*^*β-arrestin-2*^*–/–*^ mice fed with chow diet using a glucose monitor (OneTouch; LifeScan Inc., Milpitas, CA, USA), following a 16-h fasting.

### Morphology of aortas

The AngII- or saline-infused mice were sacrificed, perfused with 4% paraformaldehyde, and the aortas were isolated from the mice. Aortas were fixed on a wax plate with a scale bar to measure the aortic diameters. The maximal diameters of the suprarenal aortas were used in the statistical analysis. Aortas with diameters > 1.5-fold larger than the controls were considered to have aneurysms. Following the measurement of the maximal aortic diameter, the suprarenal regions of abdominal aortas immediately proximal to the right renal branch were excised. Serial cryosections (6 μm thick, 300 μm apart) were analyzed by performing Gomori staining or Verhoeff-Von Gieson (VVG) staining for elastin assessments. Elastin degradation was graded as 1, < 25% degradation; 2, 25%–50% degradation; 3, 50%–75% degradation; or 4, > 75% degradation. The data were evaluated by two independent investigators in a blinded manner and are presented as the means of 16 serial sections from each mouse.

### Dual immunofluorescence staining

Frozen aortic sections were incubated with antibodies against CD4 (1:200, BD Biosciences), Mac-3 (1:50, Santa Cruz Biotechnology), or CD45 (1:200, BD Biosciences), followed by a secondary TRITC-conjugated goat anti-rat IgG (1:400) or FITC-conjugated goat anti-mouse IgG (1:300) antibody (Rockland Inc., Gilbertsville, PA, USA). Nuclei were counterstained with Hoechst 33342. The negative control was the primary antibody replaced with the corresponding species-specific IgG. The fluorescence signal was monitored using confocal laser scanning microscope (Leica Microsystems).

### Cytokine measurement by ELISA

Suprarenal aortas from the mice that had been subcutaneously infused with AngII (1000 ng/kg/min) for 7 days were incubated in culture medium for 20 h. The levels of MCP-1 and IL-6 secreted in the conditioned medium were determined using commercially available ELISA kits (Biovision, Inc., Milpitas, CA, USA).

### Gelatin zymography

Suprarenal aortas from C57BL/6 J mice that had been subcutaneously infused with AngII (1000 ng/kg/min) for 7 days were excised and incubated in culture medium for 20 h. The conditioned medium was electrophoresed on SDS-PAGE gels containing 1.0 mg/mL gelatin (Sigma-Aldrich, Saint Louis, MI, USA). Gels were washed twice with 2.5% Triton X-100, incubated for 24 h (37 °C) with zymography buffer (50 mM Tris-HCl, pH 7.5, 150 mM NaCl, 10 mM CaCl_2_, and 0.05% sodium azide) and stained with Coomassie brilliant blue.

For in situ zymography, freshly cut frozen aortic sections (6 μm) were incubated overnight at 37 °C with the fluorogenic substrate DQ-gelatin-FITC (Molecular Probes, Eugene, Oregon, USA) dissolved in zymography buffer. The gelatin with a fluorescent tag remains caged (no fluorescence) until it is cleaved by an enzyme with gelatinase activity. The proteolytic activity of MMPs was detected as green fluorescence (530 nm) using confocal microscopy. In control sections, gelatinolytic activity was inhibited by adding the ion chelator EDTA (20 mM) to the medium.

### ROS analysis

Freshly cut frozen aortic sections were incubated with dihydroethidine hydrochloride (DHE, 5 μM; Life Technologies, Grand Island, NY, USA) for 30 min at 37 °C to reveal the presence of ROS as red fluorescence (585 nm) under a confocal microscope.

### Bone marrow transplantation

Bone marrow transplantation was performed as described in a previous report.^37^ WT male mice (8 weeks old) were exposed to γ-irradiation from ^60^Co (Department of Applied Chemistry, Peking University) followed by the injection of bone marrow cells (5 × 10^6^ cells/mice) from WT or *COMP*^*–/–*^ mice (6 weeks old) via the tail vein. Following 6-week post-transplantation, bone marrow reconstitution was evaluated by blood genotyping and routine blood tests. Then, the reconstituted mice were fed till 5 months old, and were infused with AngII as described above.

### Recombinant adenovirus construction and infection

An adenovirus encoding the full-length mouse *COMP* cDNA (PubMed# NM_016685.2) (Ad-COMP) was constructed and amplified according to the manufacturer’s protocol (BD Biosciences, Franklin Lakes, NJ, USA). An adenovirus carrying β-galactosidase (LacZ) was used as a negative control. For production of peptidomimetic, another adenovirus (Ad-COMP-EGF2) expressing EGF2 domain of COMP (aa 125–177), sequentially fused with BM40 secretory signal peptide and Flag-tag at its N-terminus, was constructed, whereas an adenovirus encoding GFP was applied as a negative control. For in vivo studies, 1 × 10^9^ pfu of adenovirus dissolved in a 30% pluronic gel solution were perivascularly delivered to the suprarenal aortas as described previously.^73^ Three days after the periaortic application of adenovirus, the overexpression efficiency was evaluated by western blotting, immunohistochemical staining or real-time PCR analysis of suprarenal aortic tissues.

### Analysis of AngII concentrations

AngII concentrations in the culture medium were measured using an [^125^I]-AngII radioimmunoassay kit (Bühlmann Laboratories, Basel, Switzerland).^4^ The samples, calibrators and controls were first preincubated with an anti-AngII antibody for 16 h. [^125^I]-AngII was then added and competed with AngII present in samples, calibrators and controls for the same antibody-binding sites in a second 6-h incubation step. After this incubation, a solid-phase secondary antibody was added to the mixture. The antibody-bound fraction was precipitated and counted in a gamma counter. The radioactive values in calibrators were regressed with respective AngII concentrations into the standard curve. Based on this curve, the concentrations of AngII in samples were calculated.

### Quantitative real-time PCR

Real-time PCR amplification involved the use of an Mx3000 Multiplex Quantitative PCR System (Stratagene Corp, La Jolla, CA, USA) and the SYBR Green I reagent. The relative mRNA level was calculated using 2^–ΔΔCt^ method. The ΔCt was computed with normalization to the internal control β-actin. Then, the ΔCt of untreated or control cells for each independent experiment was chosen as a calibrator for in vitro studies, whereas the mean of ΔCt in the untreated, WT or *ApoE*^*–/–*^ samples was used as a calibrator for in vivo experiments. The primer sequences for real-time PCR are shown in Supplementary information, Table S10.

### Cell culture and plasmid transfection

Rat VSMC, COS-7, HEK293T, HEK293A and HeLa cells were maintained in DMEM supplemented with 10% fetal bovine serum at 37 °C in a humidified atmosphere containing 5% CO_2_. The plasmid transfections were performed using the JetPEI DNA transfection reagent (Polyplus-transfection, Strasbourg, France), while the siRNA transfection into rat VSMCs was performed using the Lipofectamine RNAiMAX transfection reagent (Life Technologies, Grand Island, NY, USA).

### Measurement of mRNA stability

To measure the stability of COMP mRNA, rat VSMCs were pretreated with actinomycin D (2 μg/mL, Sigma, St. Louis, MO, USA) for 30 min. Total RNA of cells was then collected at 0, 2, 6, 12, and 24 h after actinomycin D treatment, followed by real-time qPCR analysis using primers for the COMP coding sequence. The turnover rate and half-life of mRNA were estimated according to a previously published paper.^74^

### RIP

The RNA (20 μg) isolated from rat VSMCs was incubated in immunoprecipitation (IP) buffer (150 nmol/L NaCl, 0.1% NP-40, 10 mmol/L Tris-HCl, pH 7.4) containing anti-AUF1 antibody (1 μg) and RNasin (1 U/μL) at 4 °C for 12 h. Then, Protein A/G agarose resin (30 μL) was added and continuously incubated at 4 °C for 2 h. After being centrifuged five times and washed with IP buffer, the AUF-1-bound RNA was enriched and subjected to real-time qPCR analysis. Moreover, rabbit IgG was applied as the negative control.

### Co-IP

Mouse suprarenal aorta lysates were incubated with an antibody against COMP (Abcam), AT1 (ProteinTech) or Flag (Sigma-Aldrich) before immunoprecipitation with Protein A/G agarose beads (Santa Cruz Biotechnology). The precipitated proteins were resolved by 10% SDS-PAGE and then immunoblotted with an antibody against AT1 or COMP. Rabbit normal IgG served as a negative control.

The cDNA encoding the mouse AT1a (NCBI Reference sequence: NM_177322.3) receptor was subcloned in-frame into the Flag vector to generate the indicated plasmids. For the co-IP assay, COS-7 cells were transfected with these plasmids and then incubated with the purified COMP protein^75^ for 30 min. The cell lysates were incubated with an anti-Flag antibody or control IgG followed by Protein A/G agarose beads, and COMP protein expression was examined using western blot analysis.

### In situ proximity ligation assay

The interaction of AT1a and COMP was analyzed in vivo using the Duolink In Situ Red Kit (Goat/Rabbit, Millipore, Danvers, MA, USA) according to the manufacturer’s instructions. Briefly, following fixation, frozen sections of mouse suprarenal aortas were incubated with a blocking solution (provided by the kit) for 1 h at room temperature and then with the primary antibodies recognizing COMP (rat, MABT36, Millipore) and AT1 (rabbit, 25343-1-AP, ProteinTech) at a 1:100 dilution overnight at 4 °C. On the following day, sections were washed and then incubated with anti-rat IgG (goat, AF005, R&D biosystems) at a 1:5000 dilution for 1 h at room temperature, followed by the labeling of two proximity ligation assay probes (PLUS and MINUS) to detect the appropriate primary antibodies for 1 h at room temperature. Sections were then washed and incubated with the ligation solution (provided by the kit) for 45 min at 37 °C, and then with the amplification solution (provided by the kit) at 37 °C for 100 min. Finally, cell nuclei were counterstained with Hoechst 33258 and sections were mounted using the Vectashield mounting medium (Vector Laboratories). The fluorescence signal was monitored using a confocal laser scanning microscope (Leica Microsystems).

### Purification of COMP and AT1 proteins

Both COMP and AT1 proteins were overexpressed using the Bac-to-Bac baculovirus expression system (Invitrogen). COMP cDNA (NM_016685.2) fused with a His-tag sequence at its C-terminus (COMP-His) and AT1 cDNA (NM_000685.4, with truncation of the sequences encoding the 40 amino acids close to C-terminus) encoding 1–318 amino acids with an N-terminal sequential fusion of the Flag-tag and BRIL sequences (Flag-BRIL-AT1) were cloned into the pFastBac vector for protein expression, respectively. The recombinant Bacmid DNA was transfected into *Spodoptera frugiperda* (Sf9) insect cells to produce AT1 and COMP proteins.

To purify COMP protein, the conditioned media of Bacmid-expressed Sf9 cells were flew through the cOmplete® His-tag affinity resin column (Roche). The resin was further washed with 20-column volumes of washing buffer (20 mM HEPES, pH 7.5, 100 mM NaCl, 2 mM EDTA, 20 mM Imidazole), and then resin-bound proteins were eluted by the buffer containing 20 mM HEPES (pH 7.5), 100 mM NaCl, 2 mM EDTA and 150 mM Imidazole. The eluent was concentrated using an Amicon® ultra centrifugal filter (100 kDa cutoff) and further purified over the gel filtration column (Superdex® 200 Increase 10/300 GL; GE Healthcare). Peak fractions of COMP protein were pooled, verified by SDS-PAGE and further concentrated to 4 mg/mL.

To purify AT1 protein, lysates of Sf9 cells infected with AT1 baculovirus were purified by M1 anti-FLAG affinity resin column and size exclusion column (Superose® 6 Increase 10/300 GL; GE Healthcare) following our previous approaches in purification of other GPCRs.^48^ Peak fractions of AT1 protein were pooled and further concentrated to 2 mg/mL.

### SPR

The interaction between purified COMP and AT1 proteins was analyzed using the BiaCore 8 K system in a running buffer containing 10 mM HEPES (pH 7.4), 150 mM NaCl, 0.05% (v/v) Surfactant P20, 0.01% (w/v) DDM and 0.002% (w/v) CHS. AT1 protein was immobilized to the surface of a CM5 sensor chip using the amine-coupling procedure at a density of ~4500 resonance units (RUs). COMP protein diluted in running buffer was injected as an analyte with the concentrations in serial two-fold dilutions for 120 s, as the period of association. Subsequently, the running buffer was alternatively perfused over the chip to allow the bound COMP to undergo a 200-s period of disassociation. The sensorgrams were record in real time during the experiment and data were analyzed in the BiaCore 8 K system using a 1:1 binding model for calculation of association constant (*K*_a_), dissociation constant (*K*_d_) and affinity (*K*_D_). In addition, TSP-1 protein was applied as a negative control for the interaction with the immobilized AT1 receptor on chip, following the identical procedure of COMP administration.

To evaluate the activity of immobilized AT1 receptor on CM5 chip, the interaction between AngII and AT1 was analyzed using the BiaCore T200 system. AngII diluted in the same running buffer as mentioned above was injected as an analyte with the concentrations in serial two-fold dilutions for 60 s, as the period of association. Subsequently, the running buffer was alternatively perfused over the chip to allow the bound AngII to undergo a 120-s period of disassociation. The altered response units (ΔRUs) at steady state were plotted as AngII bound and fitted to the Langmuir equation with corresponding AngII concentrations to yield the *K*_D_ of the AngII–AT1 interaction.

### Cell-surface ELISA

The AT1 expression on cell surface was evaluated by the cell-surface ELISA of Flag-tag without permeability on plasmid-transfected HEK293T cells. HEK293T cells were transfected with varying concentrations of plasmid encoding target receptor, including Flag-tagged AT1 with or without insertion of TC or Flag-tagged AT2 with or without insertion of TC, or co-transfected with Flag-tagged AT1 together with G protein probes, or Flag-tagged AT1-YFP together with β-arrestin-1-RLuc or β-arrestin-2-RLuc. Forty-eight hours after transfection, the cells were fixed with DPBS containing 4% formaldehyde for 5 min. Cells were washed 3 times in Tris-buffered saline containing Tween 20 (1:1000, TBST) and nonspecific binding sites were blocked by incubating cells for 1 h in blocking solution (5% BSA in TBST). Cells were washed 3 times and incubated for 1 h with anti-Flag M2 monoclonal antibody (Sigma-Aldrich, 1:1000). After 3 washes in TBST, the cells were then incubated for 1 h with HRP-conjugated goat anti-mouse IgG antibody (1:3000). The cells were further incubated with TMB/E solution (EMD Millipore, Billenca, MA, USA) and 0.25 M HCl was subsequently added to stop the reaction. The HRP activity was determined by measuring the absorbance at 450 nm using an Infinite M200 PRO microplate reader (Tecan, Männedorf, Switzerland).

### FlAsH-BRET assay

#### Intermolecular

A plasmid encoding mouse COMP fused with the modified RLuc moiety at the C-terminus and a series of human AT1 expression plasmids in which a TC-tag was inserted at G97–N98 in extracellular loop 1 (ECL1), I165–H166 in ECL2 and I271–R272 in ECL3 were generated to evaluate the interaction between AT1 and COMP. HEK293T cells were co-transfected with the COMP-RLuc plasmid and TC-tagged AT1 plasmids using Lipofectamine 2000 (Life Technologies, Grand Island, NY, USA). At 48-h post-transfection, the TC-tag of AT1 in HEK293A cells was labeled with 2.5 μM FlAsH-ETD_2_ solution using a TC-FlAsH II In-Cell Tetracysteine Tag Detection Kit (Thermo Scientific) according to the manufacturer’s instructions. Then, BRET between RLuc and FlAsH acceptor was measured after the addition of the RLuc substrate coelenterazine-h (5 μM, Promega) using a Mithras LB940 microplate reader (Berthold Technologies) equipped with BRET filter sets. The BRET signal was determined by calculating the ratio of the light intensity emitted by fluorescence acceptor (530 nm) over the light intensity emitted by RLuc (485 nm). Net BRET was calculated by subtracting the BRET ratio obtained in the sample before labeling from the ratio obtained in the respective sample with FlAsH labeling. The change in net BRET due to the interaction between AT1 and COMP was reported as ΔBRET. In addition, the N-terminus of AT1-ECL1-FlAsH was replaced with that of AT2 by PCR-directed homologous DNA recombination (Vazyme Inc., Nanjing, China), and then was utilized in this assay to verify the role of N-terminus in the AT1–COMP interaction.

The labeling efficiency of FlAsH to the TC-tags at different ECLs of AT1 was verified according to previous reports.^76,77^ Briefly, after labeling of FlAsH-ETD_2_, the cell membrane was dounce-homogenized, differentially centrifugated, and resuspended in cold assay buffer (50 mM Tris-HCl, pH 7.4, 150 mM NaCl, 12.5 mM MgCl_2_, 0.2% BSA). The membrane fractions were distributed into a 96-well black plate and treated with or without 5 mM BAL (1,2-dimercaptopropanol) for 10 min. The fluorescence intensity was measured in an EnVision multi-label microplate detector (Perkin Elmer) with the 460 nm excitation and 500–600 nm emission spectrum.

#### Intramolecular

Plasmids encoding the mouse AT1a receptor with a TC-tag inserted into a β-arrestin-sensitive site (Q229–K230) or a Gq-sensitive site (K224–A225) in the ICL3 and a modified RLuc moiety fused to the C-terminus were generated as β-arrestin- or Gq-biased sensor, respectively, to measure the conformational changes in AT1a as previously reported.^4^ Following transfection and FlAsH labeling, a BRET assay was performed as described above. The cells were preincubated with or without COMP or losartan before stimulation with an increasing amount of ligands (AngII, TRV056 or TRV027). The change in BRET due to addition of AngII, TRV027 or TRV056 was recorded as Δ net BRET.

To verify the functionality of AT1-ECL-FlAsH constructs, RLuc was fused at the N-terminus of these AT1-ECL-FlAsH plasmids. Following transfection and FlAsH labeling, the AngII-induced BRET signal was recorded as described above.

### Mammalian two-hybrid assay

Fragments encoding the 4 functional domains of mouse COMP (i.e., the N-terminus (N; aa 20–83), EGF repeat domain (EGF; aa 84–261), type III repeat domain (type III; aa 266–520), and C-terminus (C; aa 521–755)), as well as a series of truncated EGF repeats (EGF1–3: aa 85–220; EGF1–2: aa 85–177; EGF1: aa 85–124; EGF2: aa 125–177) were amplified by PCR and subcloned in-frame into the *Sal*I/*Eco*RV sites of pACT to generate pACT-COMP-N, pACT-COMP-EGF, pACT-COMP-type III, and pACT-COMP-C, respectively. The cDNAs encoding various domains of the mouse AT1a (NCBI Reference sequence: NM_177322.3) receptor (N-terminus (N; aa 1–27), transmembrane region (TM) 1–3 (aa 28–142), TM4–7 (aa 143–299), and C-terminus (C; aa 300–359)) were subcloned in-frame into the pBIND vector to generate the indicated plasmids. COS-7 cells were co-transfected with the target and bait constructs, together with the reporter plasmid pG5luc-luciferase at a ratio of 1:1:1. After 48 h, the transfected cells were harvested, and the cell lysates were used for a luciferase assay with the Dual-Luciferase Reporter Assay System (Promega, Madison, WI, USA).

### Competitive radio-ligand binding assay

HEK293T cells were transfected with AT1 or AT1–β-arrestin-2 fusion plasmids as described previously.^60^ Forty-eight hours after transfection, cells were washed twice with serum-free DMEM containing 1 mg/mL BSA (DMEM/BSA), followed by incubation with or without 5 μg/mL COMP. Then the cells were washed and then incubated with DMEM/BSA containing 1 nM [^125^I]-AngII in the presence of different concentrations of unlabeled AngII (10^–14^–10^–5^ M) at 37 °C for 1 h. The reaction was terminated by washing cells with cold Hank’s balanced salt solution on ice. The cells were lysed by 0.5 M NaOH and collected using cotton swabs into polystyrene tubes. The radioactivity was detected in a gamma counter. Nonspecific binding was defined as the binding in the presence of 10 μM unlabeled AngII and was subtracted from the data. Each concentration point was measured in triplicate. The maximal binding (B_max_) and dissociation constants were determined using GraphPad Prism 8.0 software (GraphPad Software, San Diego, CA, USA).

### BRET measurement

#### G protein activation

Gq BRET probes (Gq-RLucII, Gβ1, Gγ1-GFP10) and Gi BRET probes (Gi1-RLuc8, Gβ3, Gγ9-GFP10) were generated according to previous reports.^53,78^ G protein activation BRET assay was performed as previously described.^53^ Briefly, HEK293T cells were transiently co-transfected with WT or chimeric AT1 or AT2 plasmid together with Gq or Gi BRET probes. Twenty-four hours after transfection, cells were distributed into a 96-well microplate (Corning Inc., Corning, NY, USA) for additional 24 h and then preincubated with or without COMP protein or losartan for 30 min, followed by stimulation with an increasing amount of AngII for 2 min. BRET^2^ between RLucII and GFP10 or between RLuc8 and GFP10 was measured after the addition of the substrate coelenterazine 400a (5 μM, Interchim) using a Mithras LB940 multimode reader (Berthold Technologies). The BRET^2^ signal was calculated as the ratio of emission of GFP10 (510 nm) to RLucII or RLuc8 (400 nm).

#### β-arrestin recruitment

HEK293T cells were transiently co-transfected with the YFP-tagged or TC-inserted AT1 together with β-arrestin-1-RLuc or β-arrestin-2-RLuc plasmids. Twenty-four hours after transfection, cells were distributed into a 96-well microplate (Corning Inc., Corning, NY, USA) for additional 24 h and then preincubated with or without COMP protein or losartan for 30 min, followed by stimulation with an increasing amount of AngII for 5 min. BRET between RLuc and YFP was measured after the addition of the RLuc substrate coelenterazine-h (5 μM, Promega) using a BioTek Cytation 5 plate reader. The BRET signal was calculated as the ratio of emission of YFP (540 nm) to RLuc (460 nm). To verify the functionality of AT1 functional sensors with the insertion of TC-tags at ICLs and RLuc at C-terminus, HEK293T cells were transiently co-transfected with the AT1 sensor or AT1-RLuc together with β-arrestin-2-YFP plasmids. The BRET signal was measured as described above.

To assess the effect of Ca^2+^ on the antagonistic function of COMP in AngII-induced β-arrestin recruitment, HEK293T cells were transiently co-transfected with the AT1-YFP together with β-arrestin-2-RLuc plasmids. Forty-eight hours after transfection, cells were dissociated and washed with Hank’s balanced salt solution without calcium or magnesium (Thermo Fisher Scientific) before centrifugation. Cells was resuspended in Ca^2+^-free buffer (135 mM NaCl, 5 mM KCl, 1 mM EGTA, 5 mM D-glucose, and 0.1% BSA in 10 mM HEPES, pH 7.4) or in Ca^2+^-containing buffer substituting EGTA with 1 mM CaCl_2_. Cells were preincubated with or without COMP for 30 min and stimulated with an increasing amount of AngII (COMP and AngII were dissolved in Ca^2+^-free buffer or Ca^2+^-containing buffer according to the experimental requirement) for 5 min. The BRET signal was measured as described above.

### Measurement of the intracellular Ca^2+^ mobilization

The intracellular Ca^2+^ concentration was measured using Fluo-4-AM (Solarbio, Beijing, China). HEK293A cells were incubated with 4 μM of Fluo-4-AM in HEPES buffered saline (10 mM HEPES, pH 7.4, 1 mM Na_2_HPO_4_, 137 mM NaCl, 5 mM KCl, 1 mM CaCl_2_, 0.5 mM MgCl_2_, 5 mM Glucose, 0.1% BSA) for 60 min at 37 °C in 5% CO_2_. After being rinsed with HEPES buffered saline for 3 times, HEK293A cells were distributed into a 96-well microplate (Corning Inc., Corning, NY, USA), followed by preincubation with or without COMP protein or losartan for 30 min. Then, the 96-well microplate was placed into BioTek Cytation 5 plate reader. The Ca^2+^ fluorescence was measured using the spectrum of excitation and emission at 485 nm and 525 nm, respectively. The fluorescence intensity recorded before AngII stimulation served as baseline data (F0); the fluorescence intensities were real-time recorded at 7-s intervals following administration of an increasing amount of AngII, till 2 min of stimulation. The intracellular Ca^2+^ mobilization was presented as the ratio of maximal fluorescent intensity (F) after AngII stimulation to F_0_.

### Ex vivo culture of mouse aortic rings

The suprarenal aortas were dissected from sacrificed WT, *β-arrestin-1*^*–/–*^ or *β-arrestin-2*^*–/–*^ mice without the fixation by paraformaldehyde. The aortic tissues were cut into 5 mm-thick rings and cultured in DMEM for 48 h in the absence or presence of AngII (1 μM). The conditioned media were collected for ELISA measurement of MCP-1 and IL-6.

### AT1 internalization assays

#### Confocal microscopy

HeLa cells were transfected with AT1-GFP plasmid (with GFP fused to N-terminus). Forty-eight hours post transfection, cells were pretreated with or without COMP protein (5 μg/mL) for 30 min, followed by AngII (1 μM) stimulation and real-time observation under confocal laser scanning microscope (Leica) for 15 min.

#### Flow cytometry analysis

HEK293A cells were transfected with AT1-Flag plasmid (with a Flag-tag at N-terminus). Forty-eight hours later, cells were dissociated by Accutase (Sigma-Aldrich), and grouped into 1 × 10^5^ per sample for further treatment. Following 30-min incubation with purified COMP protein (5 μg/mL), cells were stimulated with AngII for 15 min. Then, cells were incubated with PE-anti-Flag antibodies (BioLegend) for 30 min on ice. The cell surface-located AT1 receptors were analyzed by BD FACS Calibur system.

### GST and MBP pull-down assays

The N-terminal domain (aa 1–27) of mouse AT1a was subcloned into the pGEX-6P-1 vector. Fragments encoding the 4 functional domains of mouse COMP were subcloned into a pMAL-p5x vector (New England Biolabs, Beverly, MA, USA), expressed in *Escherichia coli* BL21 cells and purified according to the manufacturer’s instructions. Equimolar amounts of the GST and MBP fusion proteins were incubated at 4 °C for 2 h. The mixture was then incubated with amylose resin (50 μL) for 30 min at room temperature. The bound proteins were denatured, separated on 12% SDS-PAGE gels, and detected by western blot analysis with an anti-GST antibody.

### Statistical analyses of cell and animal studies

All results, except human study, are presented as means ± SEM. Statistical analyses were performed using GraphPad Prism 8.0 software (GraphPad Software, San Diego, CA, USA). For statistical comparisons, we first evaluated whether the data were normally distributed using Shapiro-Wilk normality test. Then, we applied Student’s *t*-test, with Welch’s correction if equal standard deviations are not assumed through an F test, for two-group comparisons of normally distributed data. In addition, the Brown-Forsythe test was used to assess equal variances among data from > 2 groups; we applied ordinary ANOVA or Welch ANOVA for equal variances assumed or not, respectively. Nonparametric tests were used when the data were not normally distributed. In all cases, a statistically significant difference was present when the two-tailed probability was < 0.05. The details of the statistical analysis applied to each experiment are presented in the corresponding figure legends.

## Supplementary information

Supplementary information, Table S1

Supplementary information, Table S2

Supplementary information, Table S3

Supplementary information, Table S4

Supplementary information, Table S5

Supplementary information, Table S6

Supplementary information, Table S7

Supplementary information, Table S8

Supplementary information, Table S9

Supplementary information, Table S10

Supplementary information, Figure S1

Supplementary information, Figure S2

Supplementary information, Figure S3

Supplementary information, Figure S4

Supplementary information, Figure S5

Supplementary information, Figure S6

Supplementary information, Figure S7

Supplementary information, Figure S8

Supplementary information, Figure S9

Supplementary information, Figure S10

Supplementary information, Figure S11

Supplementary information, Figure S12

Supplementary information, Figure S13

Supplementary information, Figure S14

Supplementary information, Figure S15

Supplementary information, Figure S16

Supplementary information, Figure S17

## References

[1] Eguchi S, Kawai T, Scalia R, Rizzo V (2018). Understanding angiotensin II type 1 receptor signaling in vascular pathophysiology. Hypertension.

[2] Forrester SJ (2018). Angiotensin II signal transduction: an update on mechanisms of physiology and pathophysiology. Physiol. Rev.

[3] Wu CH (2018). Renin-angiotensin system and cardiovascular functions. Arterioscler. Thromb. Vasc. Biol..

[4] Li T (2018). Homocysteine directly interacts and activates the angiotensin II type I receptor to aggravate vascular injury. Nat. Commun..

[5] Liu CH (2017). Arrestin-biased AT1R agonism induces acute catecholamine secretion through TRPC3 coupling. Nat. Commun..

[6] Lymperopoulos A (2009). An adrenal beta-arrestin 1-mediated signaling pathway underlies angiotensin II-induced aldosterone production in vitro and in vivo. Proc. Natl. Acad. Sci. USA.

[7] Maning J, Negussie S, Clark MA, Lymperopoulos A (2017). Biased agonism/antagonism at the AngII-AT1 receptor: Implications for adrenal aldosterone production and cardiovascular therapy. Pharmacol. Res..

[8] Wootten D, Christopoulos A, Marti-Solano M, Babu MM, Sexton PM (2018). Mechanisms of signalling and biased agonism in G protein-coupled receptors. Nat. Rev. Mol. Cell Biol..

[9] Smith JS, Lefkowitz RJ, Rajagopal S (2018). Biased signalling: from simple switches to allosteric microprocessors. Nat. Rev. Drug Discov..

[10] Wingler LM (2020). Angiotensin and biased analogs induce structurally distinct active conformations within a GPCR. Science.

[11] Suomivuori CM (2020). Molecular mechanism of biased signaling in a prototypical G protein-coupled receptor. Science.

[12] Rakesh K (2010). beta-Arrestin-biased agonism of the angiotensin receptor induced by mechanical stress. Sci. Signal.

[13] Kim KS (2012). Beta-arrestin-biased AT1R stimulation promotes cell survival during acute cardiac injury. Am. J. Physiol. Heart Circ. Physiol..

[14] Violin JD, Lefkowitz RJ (2007). Beta-arrestin-biased ligands at seven-transmembrane receptors. Trends Pharmacol. Sci..

[15] Violin JD, Crombie AL, Soergel DG, Lark MW (2014). Biased ligands at G-protein-coupled receptors: promise and progress. Trends Pharmacol. Sci.

[16] Ryba DM (2017). Long-term biased beta-arrestin signaling improves cardiac structure and function in dilated cardiomyopathy. Circulation.

[17] Sakalihasan N (2018). Abdominal aortic aneurysms. Nat. Rev. Dis. Primers.

[18] Schermerhorn ML (2015). Long-term outcomes of abdominal aortic aneurysm in the medicare population. N. Engl. J. Med..

[19] Golledge J (2019). Abdominal aortic aneurysm: update on pathogenesis and medical treatments. Nat. Rev. Cardiol..

[20] Jones GT (2008). Angiotensin II type 1 receptor 1166C polymorphism is associated with abdominal aortic aneurysm in three independent cohorts. Arterioscler. Thromb. Vasc. Biol..

[21] Iida Y (2012). Efficacy and mechanism of angiotensin II receptor blocker treatment in experimental abdominal aortic aneurysms. PLoS One.

[22] Xuan H (2018). Inhibition or deletion of angiotensin II type 1 receptor suppresses elastase-induced experimental abdominal aortic aneurysms. J. Vasc. Surg..

[23] Morris DR (2015). TElmisartan in the management of abDominal aortic aneurYsm (TEDY): The study protocol for a randomized controlled trial. Trials.

[24] Morris DR (2016). Erratum to: ‘TElmisartan in the management of abDominal aortic aneurYsm (TEDY): The study protocol for a randomized controlled trial’. Trials.

[25] Wang M (2016). Cartilage oligomeric matrix protein prevents vascular aging and vascular smooth muscle cells senescence. Biochem. Biophys. Res. Commun..

[26] Wang L (2010). Cartilage oligomeric matrix protein maintains the contractile phenotype of vascular smooth muscle cells by interacting with alpha(7)beta(1) integrin. Circ. Res..

[27] Liang Y (2015). Cartilage oligomeric matrix protein is a natural inhibitor of thrombin. Blood.

[28] Du Y (2011). Cartilage oligomeric matrix protein inhibits vascular smooth muscle calcification by interacting with bone morphogenetic protein-2. Circ. Res.

[29] Shantikumar S, Ajjan R, Porter KE, Scott DJA (2010). Diabetes and the abdominal aortic aneurysm. Eur. J. Vasc. Endovasc. Surg..

[30] Pafili K, Gouni-Berthold I, Papanas N, Mikhailidis DP (2015). Abdominal aortic aneurysms and diabetes mellitus. J. Diabetes Complicat.

[31] Lu H, Rateri DL, Cassis LA, Daugherty A (2008). The role of the renin-angiotensin system in aortic aneurysmal diseases. Curr. Hypertens. Rep..

[32] Zhang Y, Ramos KS (2008). The development of abdominal aortic aneurysms in mice is enhanced by benzo(a)pyrene. Vasc. Health Risk Manag..

[33] Weintraub NL (2009). Understanding abdominal aortic aneurysm. N. Engl. J. Med..

[34] Pyo R (2000). Targeted gene disruption of matrix metalloproteinase-9 (gelatinase B) suppresses development of experimental abdominal aortic aneurysms. J. Clin. Invest..

[35] McCormick ML, Gavrila D, Weintraub NL (2007). Role of oxidative stress in the pathogenesis of abdominal aortic aneurysms. Arterioscler. Thromb. Vasc. Biol..

[36] Tieu BC (2009). An adventitial IL-6/MCP1 amplification loop accelerates macrophage-mediated vascular inflammation leading to aortic dissection in mice. J. Clin. Invest..

[37] Fu Y (2016). Shift of macrophage phenotype due to cartilage oligomeric matrix protein deficiency drives atherosclerotic calcification. Circ. Res.

[38] Cao RY, Amand T, Ford MD, Piomelli U, Funk CD (2010). The murine angiotensin II-induced abdominal aortic aneurysm model: rupture risk and inflammatory progression patterns. Front. Pharmacol.

[39] Liu J, Daugherty A, Lu H (2015). Angiotensin II and abdominal aortic aneurysms: an update. Curr. Pharm. Des..

[40] Suresh Babu S, Joladarashi D, Jeyabal P, Thandavarayan RA, Krishnamurthy P (2015). RNA-stabilizing proteins as molecular targets in cardiovascular pathologies. Trends Cardiovasc. Med..

[41] Poduri A (2012). Regional variation in aortic AT1b receptor mRNA abundance is associated with contractility but unrelated to atherosclerosis and aortic aneurysms. PLoS One.

[42] Xu B, Xuan H, Iida Y, Miyata M, Dalman RL (2018). Pathogenic and therapeutic significance of angiotensin II type I receptor in abdominal aortic aneurysms. Curr. Drug Targets.

[43] Cassis LA, Rateri DL, Lu H, Daugherty A (2007). Bone marrow transplantation reveals that recipient AT1a receptors are required to initiate angiotensin II-induced atherosclerosis and aneurysms. Arterioscler. Thromb. Vasc. Biol..

[44] Nakao T (2017). Genetic ablation of microRNA-33 attenuates inflammation and abdominal aortic aneurysm formation via several anti-inflammatory pathways. Arterioscler. Thromb. Vasc. Biol..

[45] Zhang C (2016). Matricellular protein CCN3 mitigates abdominal aortic aneurysm. J. Clin. Invest..

[46] Schepers D (2018). A mutation update on the LDS-associated genes TGFB2/3 and SMAD2/3. Hum. Mutat..

[47] Habashi JP (2011). Angiotensin II type 2 receptor signaling attenuates aortic aneurysm in mice through ERK antagonism. Science.

[48] Yang F (2018). Allosteric mechanisms underlie GPCR signaling to SH3-domain proteins through arrestin. Nat. Chem. Biol..

[49] Takezako T, Unal H, Karnik SS, Node K (2017). Current topics in angiotensin II type 1 receptor research: Focus on inverse agonism, receptor dimerization and biased agonism. Pharmacol. Res..

[50] Thomsen ARB (2016). GPCR-G protein-beta-arrestin super-complex mediates sustained G protein signaling. Cell.

[51] Wei H, Ahn S, Barnes WG, Lefkowitz RJ (2004). Stable interaction between beta-arrestin 2 and angiotensin type 1A receptor is required for beta-arrestin 2-mediated activation of extracellular signal-regulated kinases 1 and 2. J. Biol. Chem..

[52] Cahill TJ (2017). Distinct conformations of GPCR-beta-arrestin complexes mediate desensitization, signaling, and endocytosis. Proc. Natl. Acad. Sci. USA.

[53] Namkung Y (2018). Functional selectivity profiling of the angiotensin II type 1 receptor using pathway-wide BRET signaling sensors. Sci. Signal..

[54] McCrink KA (2017). Cardiac βarrestin2 improves contractility and adverse remodeling in heart failure, but is underexpressed in humans. J. Am. Coll. Cardiol..

[55] Nuber S (2016). beta-Arrestin biosensors reveal a rapid, receptor-dependent activation/deactivation cycle. Nature.

[56] Hoffmann C (2005). A FlAsH-based FRET approach to determine G protein-coupled receptor activation in living cells. Nat. Methods.

[57] Galandrin S (2016). Cardioprotective angiotensin-(1-7) peptide acts as a natural-biased ligand at the angiotensin II type 1 receptor. Hypertension.

[58] Felker GM (2015). Heart failure therapeutics on the basis of a biased ligand of the angiotensin-2 type 1 receptor. Rationale and design of the BLAST-AHF study (Biased Ligand of the Angiotensin Receptor Study in Acute Heart Failure). JACC Heart Fail.

[59] Tohgo A, Pierce KL, Choy EW, Lefkowitz RJ, Luttrell LM (2002). beta-Arrestin scaffolding of the ERK cascade enhances cytosolic ERK activity but inhibits ERK-mediated transcription following angiotensin AT1a receptor stimulation. J. Biol. Chem..

[60] Strachan RT (2014). Divergent transducer-specific molecular efficacies generate biased agonism at a G protein-coupled receptor (GPCR). J. Biol. Chem..

[61] Hu QX (2014). Constitutive Galphai coupling activity of very large G protein-coupled receptor 1 (VLGR1) and its regulation by PDZD7 protein. J. Biol. Chem..

[62] Lee MH (2016). The conformational signature of beta-arrestin2 predicts its trafficking and signalling functions. Nature.

[63] Trivedi DB (2013). beta-Arrestin-2 deficiency attenuates abdominal aortic aneurysm formation in mice. Circ. Res..

[64] Hunyady L, Balla T, Catt KJ (1996). The ligand binding site of the angiotensin AT1 receptor. Trends Pharmacol. Sci..

[65] Zhang H (2015). Structure of the Angiotensin receptor revealed by serial femtosecond crystallography. Cell.

[66] Rateri DL (2012). Depletion of endothelial or smooth muscle cell-specific angiotensin II type 1a receptors does not influence aortic aneurysms or atherosclerosis in LDL receptor deficient mice. PLoS One.

[67] Poduri A (2015). Fibroblast angiotensin II type 1a receptors contribute to angiotensin II-induced medial hyperplasia in the ascending aorta. Arterioscler. Thromb. Vasc. Biol..

[68] Liu J (2016). Hyperhomocysteinaemia is an independent risk factor of abdominal aortic aneurysm in a Chinese Han population. Sci. Rep..

[69] Svensson L (2002). Cartilage oligomeric matrix protein-deficient mice have normal skeletal development. Mol. Cell Biol..

[70] Bohn LM (1999). Enhanced morphine analgesia in mice lacking beta-arrestin 2. Science.

[71] Feng M, DiPetrillo K (2009). Non-invasive blood pressure measurement in mice. Methods Mol. Biol..

[72] Kurtz TW, Griffin KA, Bidani AK, Davisson RL, Hall JE (2005). Recommendations for blood pressure measurement in humans and experimental animals: part 2: blood pressure measurement in experimental animals: a statement for professionals from the Subcommittee of Professional and Public Education of the American Heart Association Council on High Blood Pressure Research. Arterioscler. Thromb. Vasc. Biol..

[73] Zhao G (2017). Unspliced XBP1 confers VSMC homeostasis and prevents aortic aneurysm formation via FoxO4 interaction. Circ. Res..

[74] Chen CY, Ezzeddine N, Shyu AB (2008). Messenger RNA half-life measurements in mammalian cells. Methods Enzymol.

[75] Ma B (2018). Cartilage oligomeric matrix protein is a novel notch ligand driving embryonic stem cell differentiation towards the smooth muscle lineage. J. Mol. Cell Cardiol..

[76] Zurn A (2010). Site-specific, orthogonal labeling of proteins in intact cells with two small biarsenical fluorophores. Bioconjug. Chem..

[77] Hoffmann C (2010). Fluorescent labeling of tetracysteine-tagged proteins in intact cells. Nat. Protoc.

[78] Olsen RHJ (2020). TRUPATH, an open-source biosensor platform for interrogating the GPCR transducerome. Nat. Chem. Biol..

