# Supplementary material for: Cartilage oligomeric matrix protein is an endogenous β-arrestin-2-selective allosteric modulator of AT1 receptor counteracting vascular injury
Source: Cell Res. 2021 Jan 28;31(7):773–90. doi: 10.1038/s41422-020-00464-8 (PMC8249609; doi:10.1038/s41422-020-00464-8)
Supplement: Supplementary file 1 — Supplementary information, Table S1 [file 41422_2020_464_MOESM1_ESM.pdf]

## Supplementary Information, Tables

**Table S1. Characteristics of patients with abdominal aortic aneurysm and control subjects**

| | | Cases (n=88) | Controls (n=88) | Statistics* | *P* |
|---|---|---|---|---|---|
| Gender [n (%)] | | | | | |
| | Male | 67（76.1） | 67（76.1） | Matching Factor | |
| | Female | 21（23.9） | 21（23.9） | Matching Factor | |
| Age (year) | | | | | |
| | Male | 67.06±8.297 | 66.15±10.15 | Matching Factor | |
| | Female | 65.38±10.96 | 66.52±10.92 | Matching Factor | |
| BMI | | 24.22±3.46 | 23.68±3.40 | 1.009 | 0.316 |
| SBP (mmHg) | | 139.27±17.03 | 146.01±19.14 | -2.224 | 0.029 |
| DBP (mmHg) | | 81.59±10.90 | 78.85±10.05 | 1.750 | 0.084 |
| Smoking [n (%)] | | | | | |
| | Ever | 23（26.1） | 14（15.9） | 3.026 | 0.220 |
| | Current | 35（39.8） | 43（48.9） | | |
| | Never | 30（34.1） | 31（35.2） | | |
| Excessive alcohol use [n (%)] | | | | | |
| | Ever | 8（9.1） | 12（13.6） | 0.960 | 0.619 |
| | Current | 28（31.8） | 28（31.8） | | |
| | Never | 52（59.1） | 48（54.6） | | |
| Hypertension [n (%)] | | | | | |
| | No | 31（35.2） | 27（30.7） | 5.141 | 0.076 |
| | Yes, with control | 41（46.6） | 32（36.3） | | |
| | Yes, w/o control | 16（18.2） | 29（33.0） | | |
| Diabetes [n (%)] | | | | | |
| | Yes | 9（10.2） | 37（42.0） | 23.074 | <0.001 |
| | No | 79（89.8） | 51（58.0） | | |
| Coronary heart disease [n (%)] | | | | | |
| | Yes | 34（38.6） | 27（30.7） | 1.229 | 0.268 |
| | No | 54（61.4） | 61（69.3） | | |
| Heart rate | | 76.26±12.53 | 79.57±10.39 | -1.848 | 0.068 |
| Glucose (mmol/L) | | 5.22±1.37 | 5.61±2.23 | -1.341 | 0.183 |
| TG（mmol/L） | | 1.30（0.95, 1.64） | 1.42（0.95, 2.01） | -0.619 | 0.538 |
| TC（mmol/L） | | 4.33（3.57, 4.34） | 4.48（3.72, 5.10） | -0.294 | 0.769 |
| HDL（mmol/L） | | 1.03±0.23 | 1.046±0.27 | -0.273 | 0.785 |
| LDL（mmol/L） | | 2.81±1.02 | 2.86±0.97 | -0.404 | 0.688 |

*Data were present as means ± SD or median (quartile 1, quartile 3) for normally or non-normally distributed continuous variables and as frequency or percentage for categorical variables. The unpaired Student's *t*-test or the Wilcoxon signed-rank test was applied to evaluate statistical significance for continuous variables with or without normal distribution respectively. Meanwhile, the Chi-square test was used to evaluate the statistical significance between the proportions of the two groups.
AAA, abdominal aortic aneurysm; COMP, cartilage oligomeric matrix protein; BMI, body mass index ; SBP, systolic blood pressure; DBP, diastolic blood pressure; TC, total cholesterol; TG, triglyceride; HDL, high density lipoprotein; LDL, low density lipoprotein
