# Supplementary material for: Cartilage oligomeric matrix protein is an endogenous β-arrestin-2-selective allosteric modulator of AT1 receptor counteracting vascular injury
Source: Cell Res. 2021 Jan 28;31(7):773–90. doi: 10.1038/s41422-020-00464-8 (PMC8249609; doi:10.1038/s41422-020-00464-8)
Supplement: Supplementary file 2 — Supplementary information, Table S2 [file 41422_2020_464_MOESM2_ESM.pdf]

Table S2. Adjusted odds ratios for the risk of abdominal aortic aneurysm for plasma COMP levels

	Unit	P	Adjusted OR	95% CI
COMP	Log[COMP (ng/ml)]	<0.001	44.123	9.698~200.756
COMP	⩾279.9 ng/ml	<0.001	reference category	reference category
	213.8~279.9 ng/ml	<0.001	57.954	7.449~450.881
	<213.8 ng/ml	0.001	42.268	4.354~410.303

OR, odds ratio; CI, confidence interval.
