# Supplementary material for: Cartilage oligomeric matrix protein is an endogenous β-arrestin-2-selective allosteric modulator of AT1 receptor counteracting vascular injury
Source: Cell Res. 2021 Jan 28;31(7):773–90. doi: 10.1038/s41422-020-00464-8 (PMC8249609; doi:10.1038/s41422-020-00464-8)
Supplement: Supplementary file 3 — Supplementary information, Table S3 [file 41422_2020_464_MOESM3_ESM.pdf]

Table S3. Characteristics of WT and *COMP*$^{-/-}$ mice infused with saline or AngII.

Group	WT+Saline	WT+AngII	*COMP*$^{-/-}$+Saline	*COMP*$^{-/-}$+AngII
**No.**	12	21	12	33
**Weight (g)**	32.3±0.82	33.0±1.27	32.3±0.82	32.4±1.38
**SBP (mmHg)**	102.7±2.52	160.9±16.60*	111.0±5.57	162.0±14.00$^{\#}$
**TC (mM)**	1.95±0.66	2.26±0.77	1.92±0.71	2.26±0.43
**TG (mM)**	1.28±0.15	1.43±0.11	1.28±0.14	1.49±0.13

*P<0.05 *vs.* WT+Saline; $^{\#}P$<0.05 *vs.* *COMP*$^{-/-}$+Saline.

SBP, systolic blood pressure; TC, total cholesterol; TG, triglyceride.

Data are presented as means ± SEM.
