# Supplementary material for: Cartilage oligomeric matrix protein is an endogenous β-arrestin-2-selective allosteric modulator of AT1 receptor counteracting vascular injury
Source: Cell Res. 2021 Jan 28;31(7):773–90. doi: 10.1038/s41422-020-00464-8 (PMC8249609; doi:10.1038/s41422-020-00464-8)
Supplement: Supplementary file 4 — Supplementary information, Table S4 [file 41422_2020_464_MOESM4_ESM.pdf]

**Table S4. Characteristics of bone marrow transplantation-created chimeric mice infused with AngII.**

| Group | WT→WT | *COMP*$^{-/-}$ →WT |
|---|---|---|
| **No.** | 10 | 10 |
| **Weight (g)** | 26.4±1.14 | 26.8±1.48 |
| **SBP (mmHg)** | 168.0±7.83 | 165.8±7.41 |
| **AAA Incidence (%)** | 0 | 0 |
| **Maximal abdominal aortic diameter (mm)** | 0.94±0.07 | 0.93±0.09 |

SBP, systolic blood pressure.

Data are presented as means ± SEM.
