# Supplementary material for: Cartilage oligomeric matrix protein is an endogenous β-arrestin-2-selective allosteric modulator of AT1 receptor counteracting vascular injury
Source: Cell Res. 2021 Jan 28;31(7):773–90. doi: 10.1038/s41422-020-00464-8 (PMC8249609; doi:10.1038/s41422-020-00464-8)
Supplement: Supplementary file 5 — Supplementary information, Table S5 [file 41422_2020_464_MOESM5_ESM.pdf]

Table S5. Characteristics of Ad-LacZ/Ad-COMP-treated *ApoE*$^{-/-}$ mice infused with saline or AngII.

Group	Ad-LacZ+Saline	Ad-LacZ+AngII	Ad-COMP+Saline	Ad-COMP+AngII
**No.**	12	15	12	15
**Weight (g)**	30.3±0.82	31.7±0.92	30.8±0.75	32.3±1.21
**SBP (mmHg)**	105.7±2.89	170.3±10.99*	106.7±2.22	174.3±7.21$^{\#}$
**TC (mM)**	6.15±1.46	5.92±0.41	5.88±0.35	5.49±1.09
**TG (mM)**	1.74±0.24	1.58±0.68	1.64±0.26	1.60±0.40

*P<0.05 *vs.* LacZ+Saline; $^{\#}P$<0.05 *vs.* COMP+Saline.

SBP, systolic blood pressure; TC, total cholesterol; TG, triglyceride.

Data are presented as means ± SEM.
