# Supplementary material for: Cartilage oligomeric matrix protein is an endogenous β-arrestin-2-selective allosteric modulator of AT1 receptor counteracting vascular injury
Source: Cell Res. 2021 Jan 28;31(7):773–90. doi: 10.1038/s41422-020-00464-8 (PMC8249609; doi:10.1038/s41422-020-00464-8)
Supplement: Supplementary file 6 — Supplementary information, Table S6 [file 41422_2020_464_MOESM6_ESM.pdf]

Table S6. Characteristics of $ApoE^{-/-}$ and $ApoE^{-/-}$ $COMP^{SM\text{-}Tg}$ mice infused with AngII.

**Group**	$ApoE^{-/-}$	$ApoE^{-/-}$ $COMP^{SM\text{-}Tg}$
**No.**	12	10
**Weight (g)**	31.4±0.92	32.1±1.17
**SBP (mmHg)**	170.6±7.99	174.2±7.25
**TC (mM)**	5.42±0.48	5.59±1.02
**TG (mM)**	1.60±0.68	1.58±0.44

SBP, systolic blood pressure; TC, total cholesterol; TG, triglyceride.

Data are presented as means ± SEM.
