# Supplementary material for: Cartilage oligomeric matrix protein is an endogenous β-arrestin-2-selective allosteric modulator of AT1 receptor counteracting vascular injury
Source: Cell Res. 2021 Jan 28;31(7):773–90. doi: 10.1038/s41422-020-00464-8 (PMC8249609; doi:10.1038/s41422-020-00464-8)
Supplement: Supplementary file 7 — Supplementary information, Table S7 [file 41422_2020_464_MOESM7_ESM.pdf]

Table S7. Characteristics of *COMP*$^{-/-}$ and *COMP*$^{-/-}$*AT1a*$^{-/-}$ mice infused with AngII.

**Group**	***COMP*$^{-/-}$**	***COMP*$^{-/-}$*AT1a*$^{-/-}$**
**No.**	7	8
**Weight (g)**	32.3±1.56	32.1±2.50
**SBP (mmHg)**	168.26±6.62	112.48±6.93*
**TC (mM)**	2.14±0.21	2.05±0.32
**TG (mM)**	1.46±0.08	1.48±0.12

*P<0.05 *vs.* *COMP*$^{-/-}$.

SBP, systolic blood pressure; TC, total cholesterol; TG, triglyceride.

Data are presented as means ± SEM.
