# Supplementary material for: Cartilage oligomeric matrix protein is an endogenous β-arrestin-2-selective allosteric modulator of AT1 receptor counteracting vascular injury
Source: Cell Res. 2021 Jan 28;31(7):773–90. doi: 10.1038/s41422-020-00464-8 (PMC8249609; doi:10.1038/s41422-020-00464-8)
Supplement: Supplementary file 8 — Supplementary information, Table S8 [file 41422_2020_464_MOESM8_ESM.pdf]

Table S8. Characteristics of *COMP*$^{-/-}$ and *COMP*$^{-/-}$*β-arrestin 2*$^{-/-}$ mice infused with AngII.

**Group**	***COMP***$^{-/-}$	***COMP***$^{-/-}$***β-arrestin 2***$^{-/-}$
**No.**	11	7
**Weight (g)**	32.4±0.92	32.3±1.02
**SBP (mmHg)**	168.3±8.99	164.3±10.21
**TC (mM)**	1.92±0.31	2.09±0.98
**TG (mM)**	1.48±0.16	1.60±0.14
**FBG (mM)**	7.37±0.18	7.62±0.23

SBP, systolic blood pressure; TC, total cholesterol; TG, triglyceride; FBG, fasting blood glucose.

Data are presented as means ± SEM.
