# Supplementary material for: Cartilage oligomeric matrix protein is an endogenous β-arrestin-2-selective allosteric modulator of AT1 receptor counteracting vascular injury
Source: Cell Res. 2021 Jan 28;31(7):773–90. doi: 10.1038/s41422-020-00464-8 (PMC8249609; doi:10.1038/s41422-020-00464-8)
Supplement: Supplementary file 9 — Supplementary information, Table S9 [file 41422_2020_464_MOESM9_ESM.pdf]

Table S9. Characteristics of Ad-GFP/Ad-COMP-EGF2-infected *ApoE$^{-/-}$* mice infused with AngII.

Group	Ad-GFP	Ad-COMP-EGF2
**No.**	11	15
**Weight (g)**	31.0±0.87	31.7±0.98
**SBP (mmHg)**	166.3±7.69	169.3±8.27
**TC (mM)**	5.96±0.81	6.09±0.68
**TG (mM)**	1.58±0.29	1.56±0.41

SBP, systolic blood pressure; TC, total cholesterol; TG, triglyceride.

Data are presented as means ± SEM.
