# Supplementary material for: Cartilage oligomeric matrix protein is an endogenous β-arrestin-2-selective allosteric modulator of AT1 receptor counteracting vascular injury
Source: Cell Res. 2021 Jan 28;31(7):773–90. doi: 10.1038/s41422-020-00464-8 (PMC8249609; doi:10.1038/s41422-020-00464-8)
Supplement: Supplementary file 10 — Supplementary information, Table S10 [file 41422_2020_464_MOESM10_ESM.pdf]

Table S10. Primer sequences for real-time PCR.

	Forward Primer (5'-3')	Reverse Primer (5'-3')
**Mouse β-actin**	GAGACCTTCAACACCCCAGCC	TCGGGGCATCGGAACCGCTCA
**Mouse AT1a**	ACCGCTATGGAATACCGCTG	GAGACACGTGAGCAGGAACA
**Mouse IL-6**	GCTCTGGTCTTCTGGAGTTCC	GAGTTGGATGGTCTTGGTCCT
**Mouse MMP-9**	GCTGACTACGATAAGGACGGCA	TAGTGGTGCAGGCAGAGTAGGA
**Mouse EGF2**	GACGTTAATGAGTGCAATGCT	CGTGCAAACTTGTTTGTTGGA
**Mouse β-arrestin-1**	GGCTACAGGAGCGACTCATC	GGTTCTCAGCACAGAAGGCT
**Mouse β-arrestin-2**	TGGATGGGCAGCTCAAACAT	CAGCTTCACCTTGACCCTGT
**Rat AUF-1**	CAGACGAGGTGGTCATCAAA	AGAGGGACCCAACGTCATAC
**Rat HuR**	GTGAGAACGGACCAAAGAGT	GAAGGGATGCGAGAAATACAAT
**Rat COMP**	GTGACTTCGATGCTGACAAGGT	GTCTTGATAGCCGAAGATGAAGC
**Rat β-actin**	GAGACCTTCAACACCCCAGCC	TCGGGGCATCGGAACCGCTCA
