# Supplementary material for: Cartilage oligomeric matrix protein is an endogenous β-arrestin-2-selective allosteric modulator of AT1 receptor counteracting vascular injury
Source: Cell Res. 2021 Jan 28;31(7):773–90. doi: 10.1038/s41422-020-00464-8 (PMC8249609; doi:10.1038/s41422-020-00464-8)
Supplement: Supplementary file 11 — Supplementary information, Figure S1 [file 41422_2020_464_MOESM11_ESM.pdf]

Supplementary Information, Figure S1

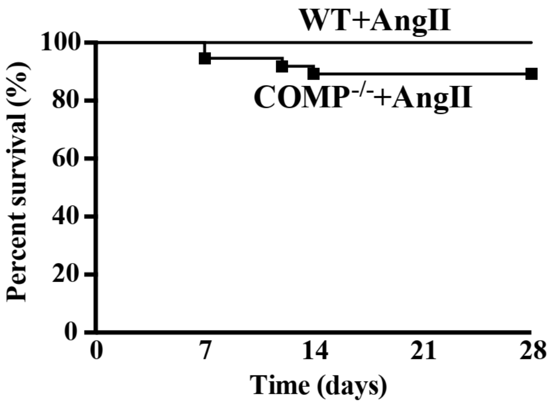

Fig. S1: Kaplan-Meier survival curves of WT (n=21) and *COMP*$^{-/-}$ mice (n=37) administered with the AngII infusion.
