# Supplementary material for: Cartilage oligomeric matrix protein is an endogenous β-arrestin-2-selective allosteric modulator of AT1 receptor counteracting vascular injury
Source: Cell Res. 2021 Jan 28;31(7):773–90. doi: 10.1038/s41422-020-00464-8 (PMC8249609; doi:10.1038/s41422-020-00464-8)
Supplement: Supplementary file 12 — Supplementary information, Figure S2 [file 41422_2020_464_MOESM12_ESM.pdf]

Supplementary Information, Figure S2

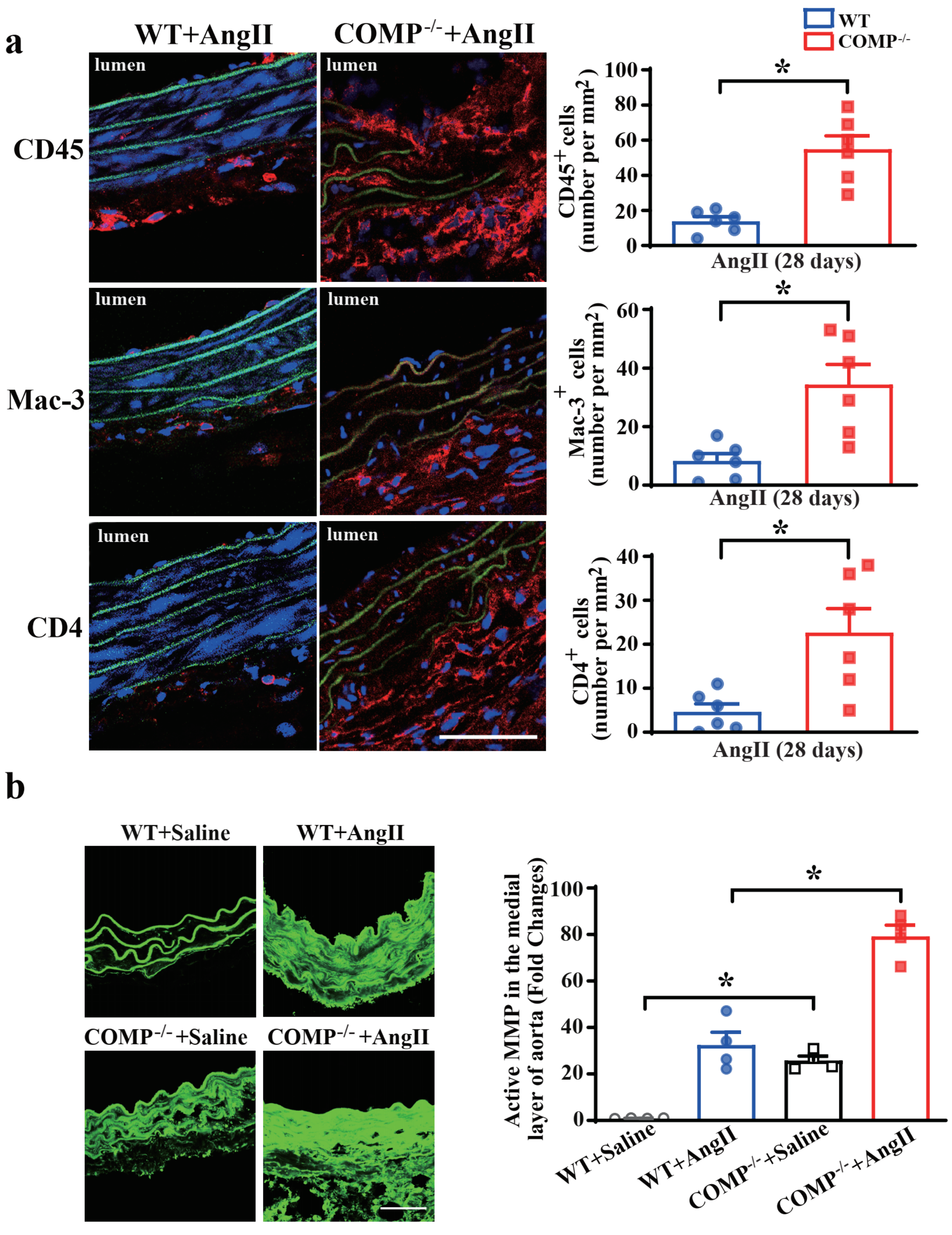

Fig. S2: a. Representative images of immunofluorescent staining (red) and quantification of leukocyte ($CD45^+$), macrophage ($Mac\text{-}3^+$) and T cell ($CD4^+$) infiltration in the adventitia of the

suprarenal aortas of WT (n=6) and *COMP*$^{-/-}$ (n=6) mice after the administration of the AngII infusion for 28 days. The nuclei were counterstained with Hoechst 33342 (blue). *P<0.05 in Mann-Whitney test. Scale bar, 50 μm. **b.** *In situ* zymography of gelatinase activity in suprarenal aortas from WT and *COMP*$^{-/-}$ mice infused with 1,000 ng/kg/min AngII or saline for 28 days. n=4 mice per group, *P<0.05 in Kruskal-Wallis test followed by Dunn's test. Scale bar, 50 μm.

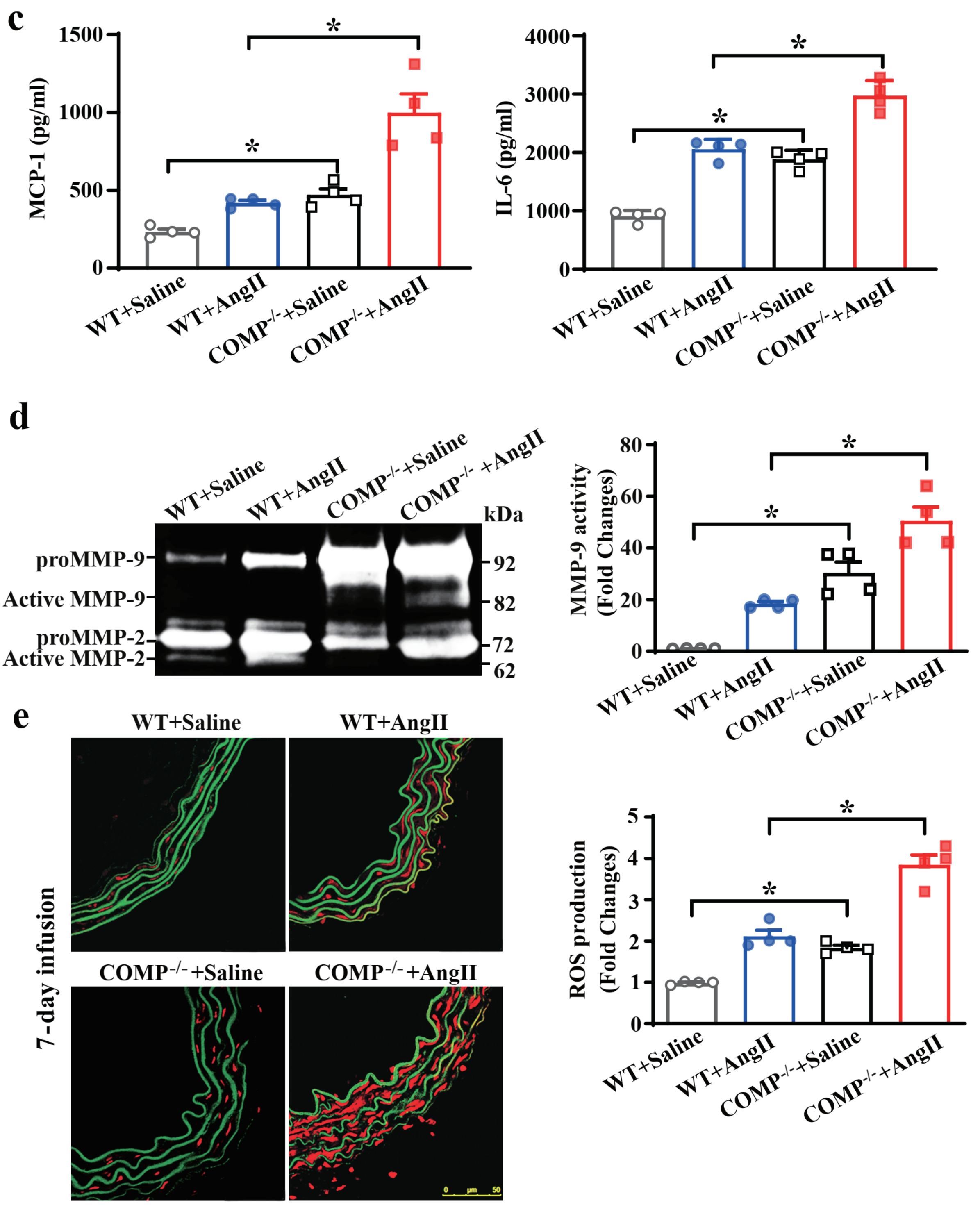

Fig. S2: c. MCP-1 and IL-6 secretion from *ex vivo* suprarenal aorta organ cultures. Aortas from WT and *COMP*$^{-/-}$ mice infused with 1,000 ng/kg/min AngII or saline for 7 days were incubated in culture medium for 20 hours, n=4 mice per group, *P<0.05 in Kruskal-Wallis test followed by Dunn's test. **d.** Representative images of gelatin zymography and quantification of

conditioned medium from suprarenal aorta organ cultures. WT and *COMP*$^{-/-}$ mice were infused with 1,000 ng/kg/min AngII or saline for 7 days, and the suprarenal aortas were incubated in culture medium for 20 hours. n=4 mice per group, **P*<0.05 in Kruskal-Wallis test followed by Dunn's test. **e.** *In situ* dihydroethidium (DHE) staining of suprarenal aortas from WT and *COMP*$^{-/-}$ mice infused with saline or AngII for 7 days. n=4 mice per group, **P*<0.05 in Mann-Whitney test. Scale bar, 50 μm.
