# Supplementary material for: Cartilage oligomeric matrix protein is an endogenous β-arrestin-2-selective allosteric modulator of AT1 receptor counteracting vascular injury
Source: Cell Res. 2021 Jan 28;31(7):773–90. doi: 10.1038/s41422-020-00464-8 (PMC8249609; doi:10.1038/s41422-020-00464-8)
Supplement: Supplementary file 13 — Supplementary information, Figure S3 [file 41422_2020_464_MOESM13_ESM.pdf]

Supplementary Information, Figure S3

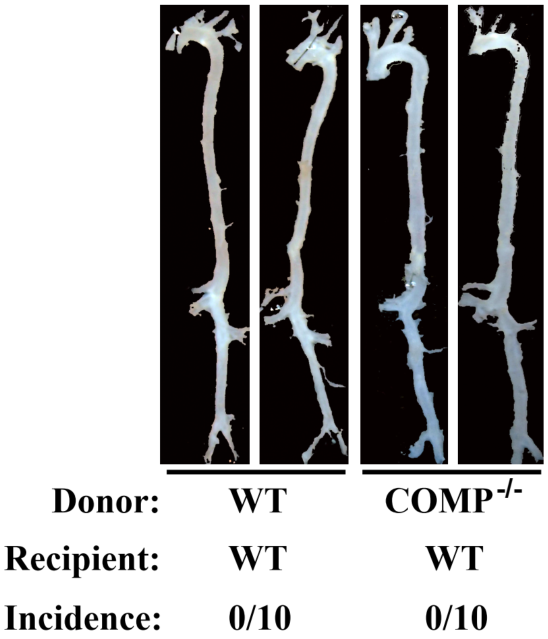

Fig. S3: Representative images of morphological features of AAA in lethally irradiated 5-month-old male WT mice receiving WT and *COMP*$^{-/-}$ bone marrow, then infused with 1,000 ng/kg/min AngII for 28 days (n=10 per group).
