# Supplementary material for: Cartilage oligomeric matrix protein is an endogenous β-arrestin-2-selective allosteric modulator of AT1 receptor counteracting vascular injury
Source: Cell Res. 2021 Jan 28;31(7):773–90. doi: 10.1038/s41422-020-00464-8 (PMC8249609; doi:10.1038/s41422-020-00464-8)
Supplement: Supplementary file 14 — Supplementary information, Figure S4 [file 41422_2020_464_MOESM14_ESM.pdf]

Supplementary Information, Figure S4

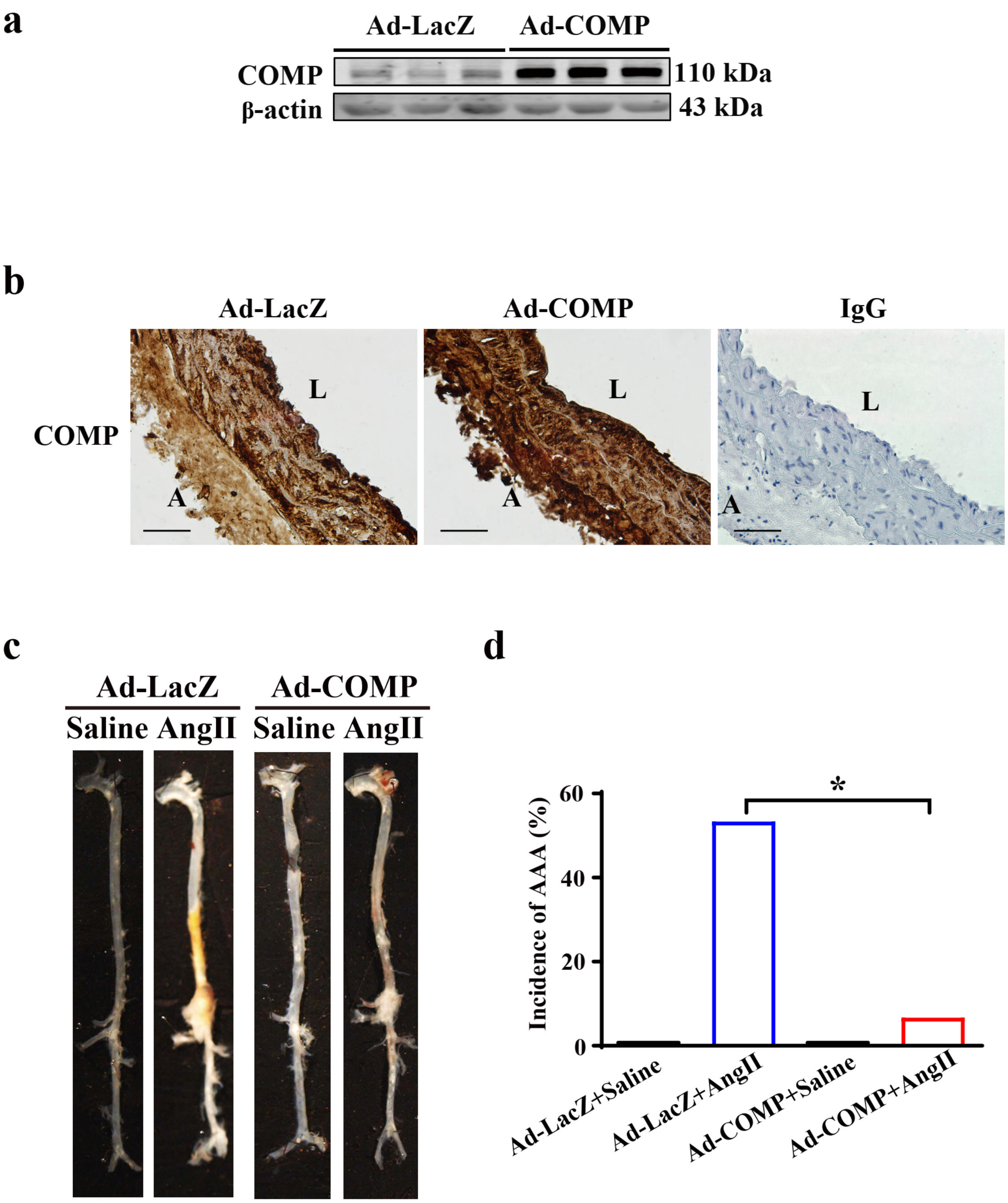

Fig. S4: a. Western blot analysis of the COMP protein in suprarenal aortas from *ApoE*$^{-/-}$ mice infected with 10^9 pfu Ad-COMP or Ad-LacZ for 3 days. **b.** Immunohistochemical staining for COMP in suprarenal aortas from *ApoE*$^{-/-}$ mice infected with 10^9 pfu Ad-COMP or Ad-LacZ for 3 days. L, lumen; A, adventitia; Scale bar, 20 μm. **c.** Representative images of

morphological features of AAA in periadventitially infected 4-month-old male *ApoE*$^{-/-}$ mice infused with 1,000 ng/kg/min AngII for 28 days. **d.** Incidence of AAA (Ad-LacZ+Saline: 0/12; Ad-LacZ+AngII: 8/15; Ad-COMP+Saline: 0/12; Ad-COMP+AngII: 1/15). *P<0.05 in Chi-square test.

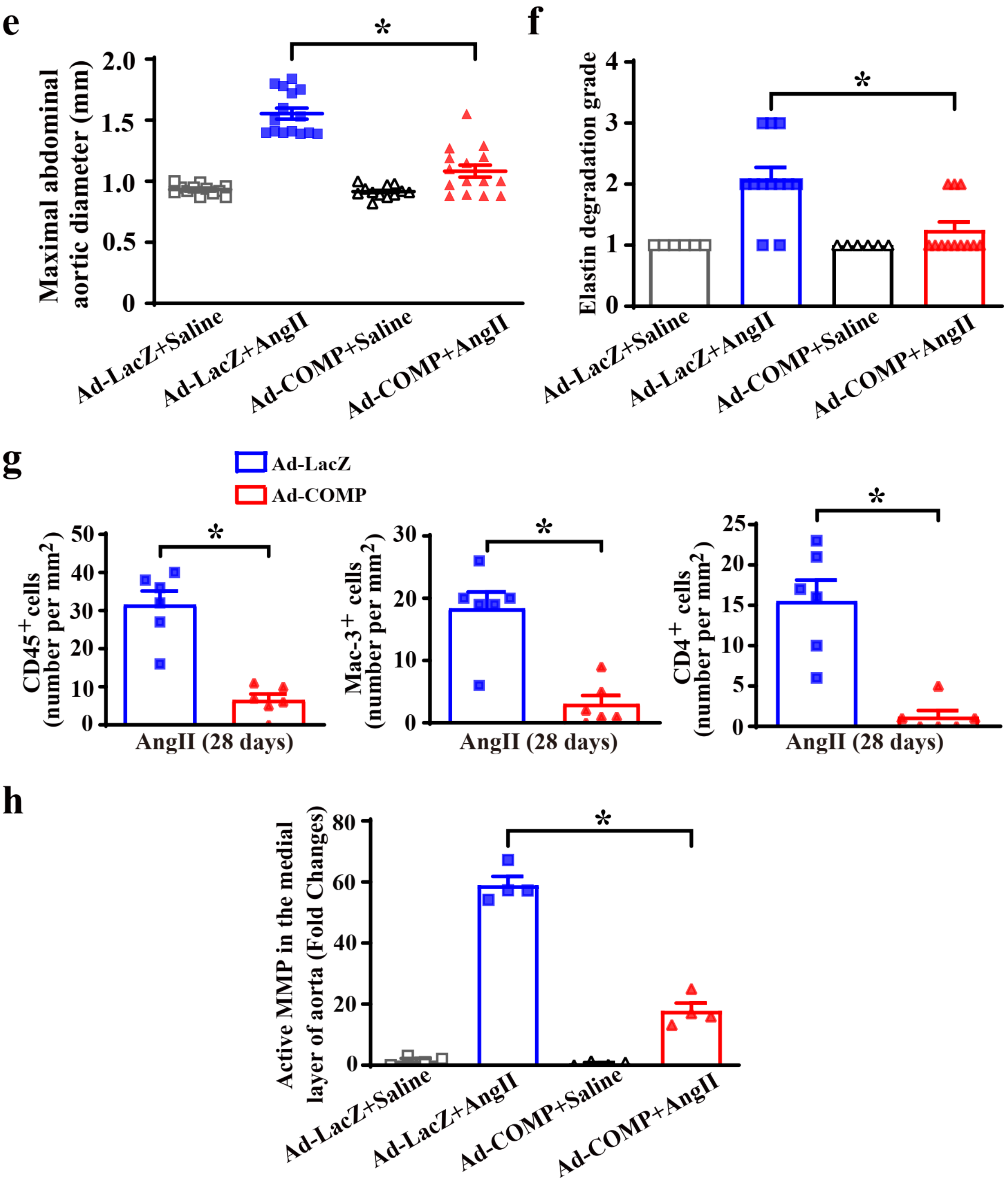

Fig. S4: e. The maximal abdominal aortic diameter. Kruskal-Wallis test followed by Dunn's test, *P<0.05. **f.** Quantification of elastin degradation. *P<0.05 in Kruskal-Wallis test followed by Dunn's test. **g.** Quantification of leukocyte ($CD45^+$), macrophage ($Mac\text{-}3^+$), and T cell ($CD4^+$) infiltration in the adventitia of suprarenal aortas in *ApoE*$^{-/-}$ mice following an AngII infusion for 28 days. n=6, *P<0.05 in Mann-Whitney test. **h.** *In situ* zymography of gelatinase activity in abdominal aortas from *ApoE*$^{-/-}$ mice infused with saline or AngII for 28 days. n=4, *P<0.05 in Kruskal-Wallis test followed by Dunn's test.

i

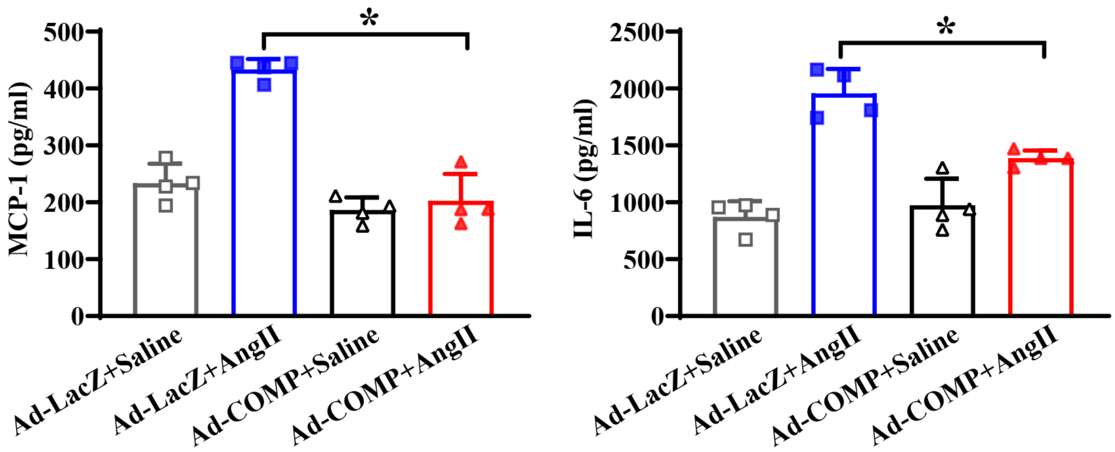

j

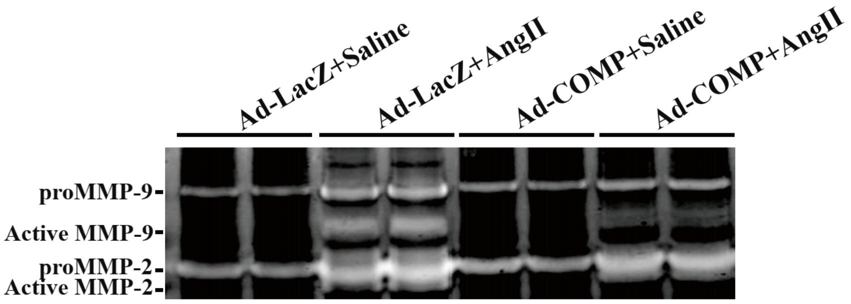

k

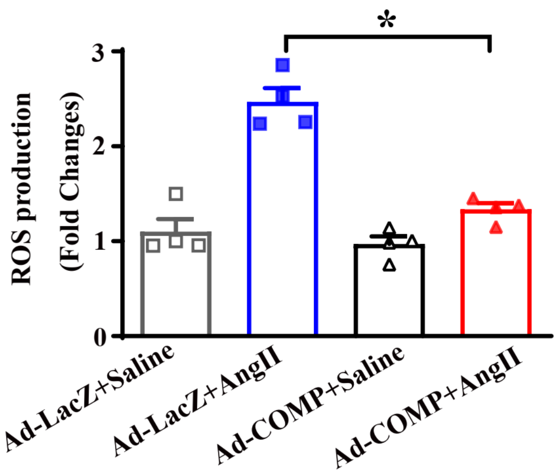

Fig. S4: i. MCP-1 and IL-6 secretion from suprarenal aorta organ cultures. Suprarenal aortas of *ApoE*$^{-/-}$ mice that had been periadventitially infected with Ad-LacZ or Ad-COMP. After 3 days, the mice were infused with 1,000 ng/kg/min AngII or saline for 7 days. Suprarenal aortas from mice were incubated in culture medium for 20 hours. *P<0.05 in Kruskal-Wallis test followed by Dunn's test. **j.** Gelatin zymography of conditioned medium from suprarenal aorta

organ cultures. Aortas from *ApoE*$^{-/-}$ mice that had been infused with saline or AngII for 7 days were incubated in culture medium for 20 hours. **k.** *In situ* DHE staining of aortas and quantification of ROS production from *ApoE*$^{-/-}$ mice infused with saline or AngII for 7 days. *P<0.05 in Kruskal-Wallis test followed by Dunn's test.
