# Supplementary material for: Cartilage oligomeric matrix protein is an endogenous β-arrestin-2-selective allosteric modulator of AT1 receptor counteracting vascular injury
Source: Cell Res. 2021 Jan 28;31(7):773–90. doi: 10.1038/s41422-020-00464-8 (PMC8249609; doi:10.1038/s41422-020-00464-8)
Supplement: Supplementary file 15 — Supplementary information, Figure S5 [file 41422_2020_464_MOESM15_ESM.pdf]

Supplementary Information, Figure S5

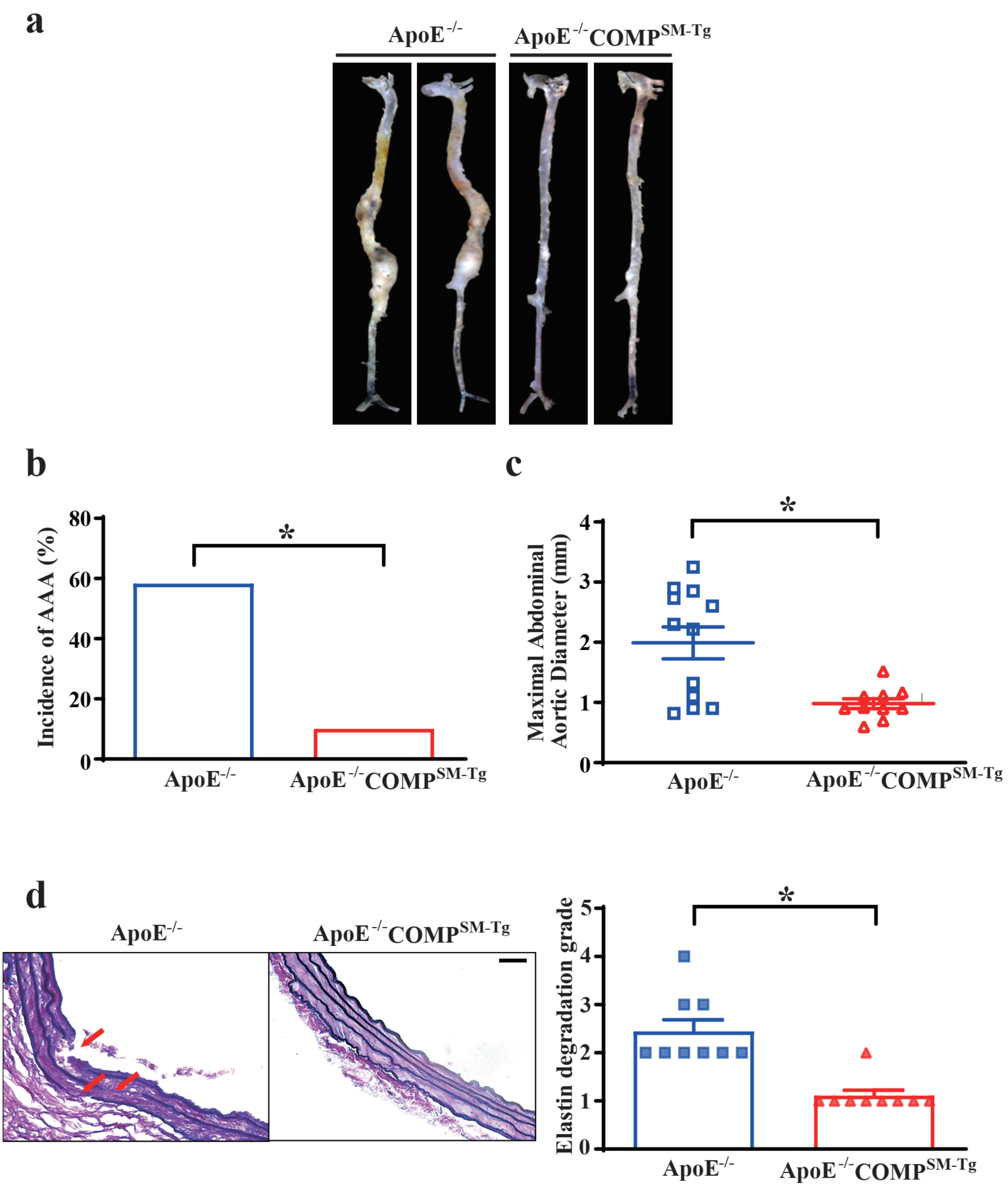

Fig. S5: a. Representative images of morphological features of AAA in 4-month-old male *ApoE*$^{-/-}$ and *ApoE*$^{-/-}$*COMP*$^{SM\text{-}Tg}$ mice infused with 1,000 ng/kg/min AngII for 28 days. **b.** Incidence of AAA (*ApoE*$^{-/-}$: 7/12; *ApoE*$^{-/-}$ *COMP*$^{SM\text{-}Tg}$: 1/10). **P*<0.05 in Chi-square test. **c.**

The maximal abdominal aortic diameter (*ApoE*$^{-/-}$: 7/12; *ApoE*$^{-/-}$ *COMP*$^{SM\text{-}Tg}$: 1/10). Mann-Whitney test, **P*<0.05. **d.** Representative images of VVG staining and quantification of elastin degradation. **P*<0.05 in Mann-Whitney test. Scale bar, 50 μm.
