# Supplementary material for: Cartilage oligomeric matrix protein is an endogenous β-arrestin-2-selective allosteric modulator of AT1 receptor counteracting vascular injury
Source: Cell Res. 2021 Jan 28;31(7):773–90. doi: 10.1038/s41422-020-00464-8 (PMC8249609; doi:10.1038/s41422-020-00464-8)
Supplement: Supplementary file 16 — Supplementary information, Figure S6 [file 41422_2020_464_MOESM16_ESM.pdf]

Supplementary Information, Figure S6

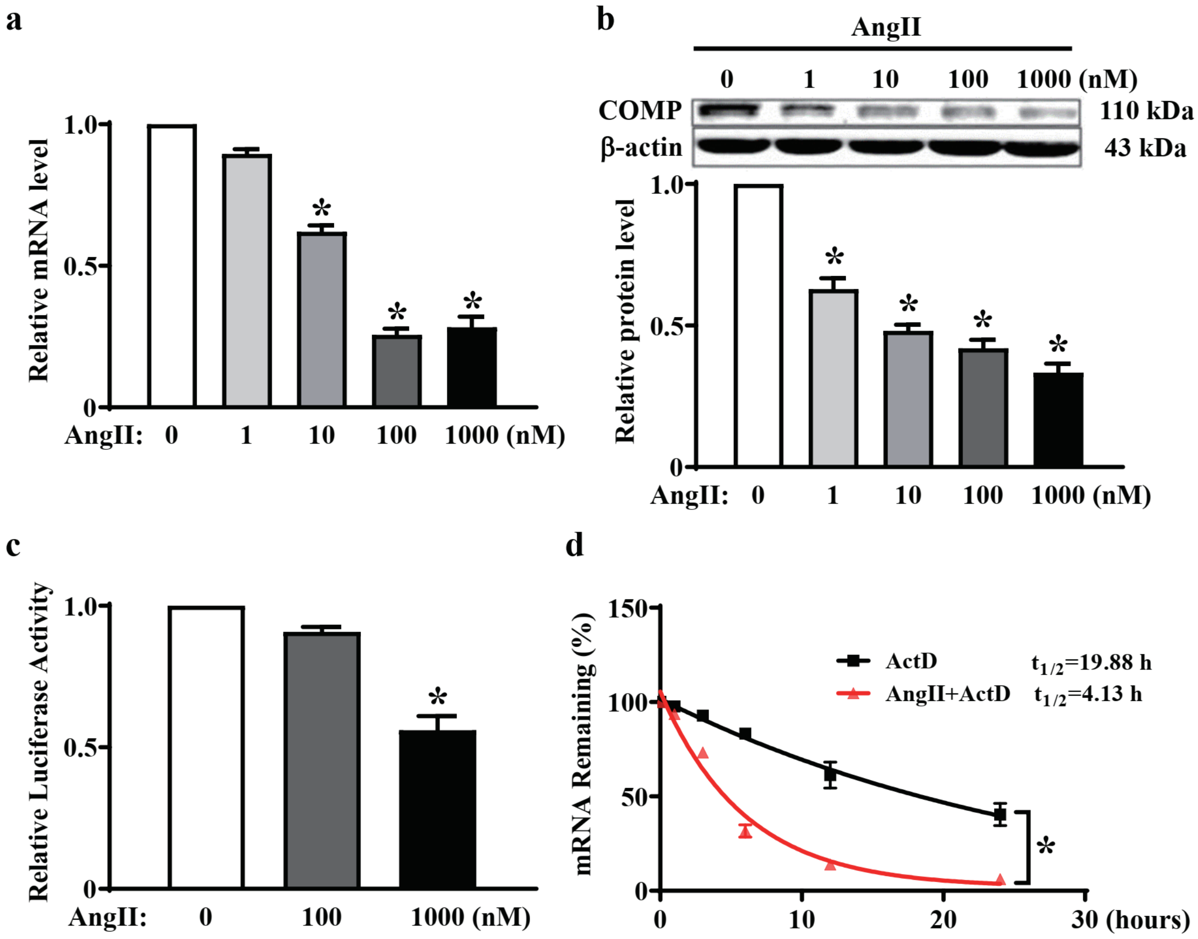

Fig. S6: a-b. Real-time PCR and western blot analysis of COMP expression in rat VSMCs treated with an increasing amount of AngII for 24 hours (**a**) or 48 hours (**b**). n=6, *P<0.05 in One-way ANOVA followed by the Bonferroni test. **c.** Luciferase assay on rat VSMCs transfected with COMP-promoter-luciferase reporter, following 24-hour of AngII stimulation. n=6, *P<0.05 in One-way ANOVA followed by the Bonferroni test. **d.** The mRNA lifetime of COMP in rat VSMCs was evaluated by real-time PCR following pretreatment with actinomycin D to inhibit transcription. n=4, *P<0.05 in the unpaired Student's *t*-test.

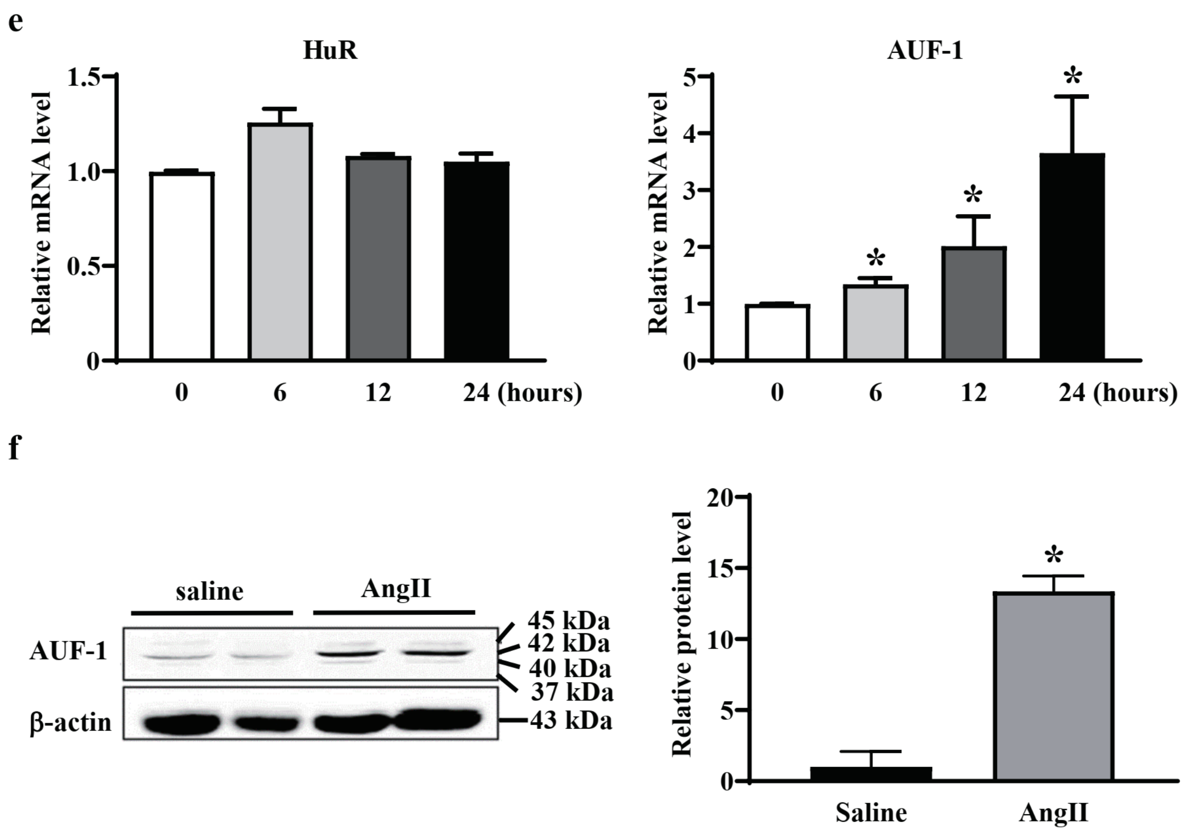

Fig. S6: e. Real-time PCR analysis of HuR and AUF-1 expression in rat VSMCs treated with AngII (0.1 μM) for various time points. n=6, *P<0.05 in One-way ANOVA followed by the Bonferroni test. **f.** Representative western blot and quantitative analysis of the AUF-1 protein level in the suprarenal aortas of *Apo*E$^{-/-}$ mice infused with 1,000 ng/kg/min AngII or saline for 7 days. n=6 mice per group, *P<0.05 in the unpaired Student's *t* test.

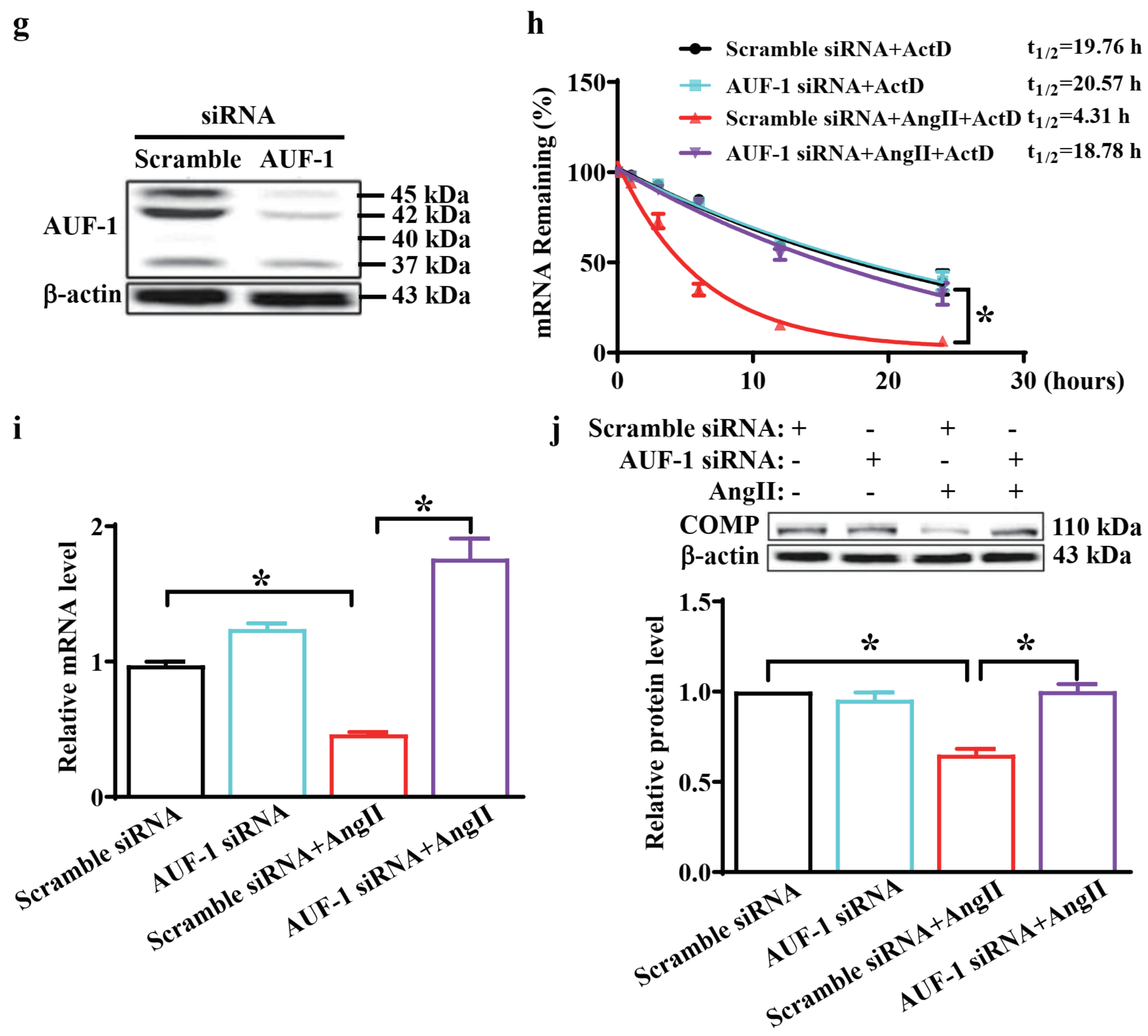

Fig. S6: g. Western blot analysis of AUF-1 expression in rat VSMCs in 72 hours after siRNA transfection. **h.** The mRNA lifetime of COMP in rat VSMCs transfected with scramble or AUF-1 siRNA followed by AngII (0.1 μM) stimulation was evaluated by real-time PCR after the pretreatment with actinomycin D to inhibit transcription. n=4, **P*<0.05 in One-way ANOVA followed by the Bonferroni test. **i.** Real-time PCR analysis of COMP expression in rat VSMCs transfected with siRNA, followed by AngII (0.1 μM) stimulation for 24 hours. n=6, **P*<0.05 in One-way ANOVA followed by the Bonferroni test. **j.** Representative western blot and quantitative analysis of COMP expression in rat VSMCs transfected with siRNA, followed by AngII (0.1 μM) stimulation for 48 hours. n=6, **P*<0.05 in One-way ANOVA followed by the Bonferroni test.

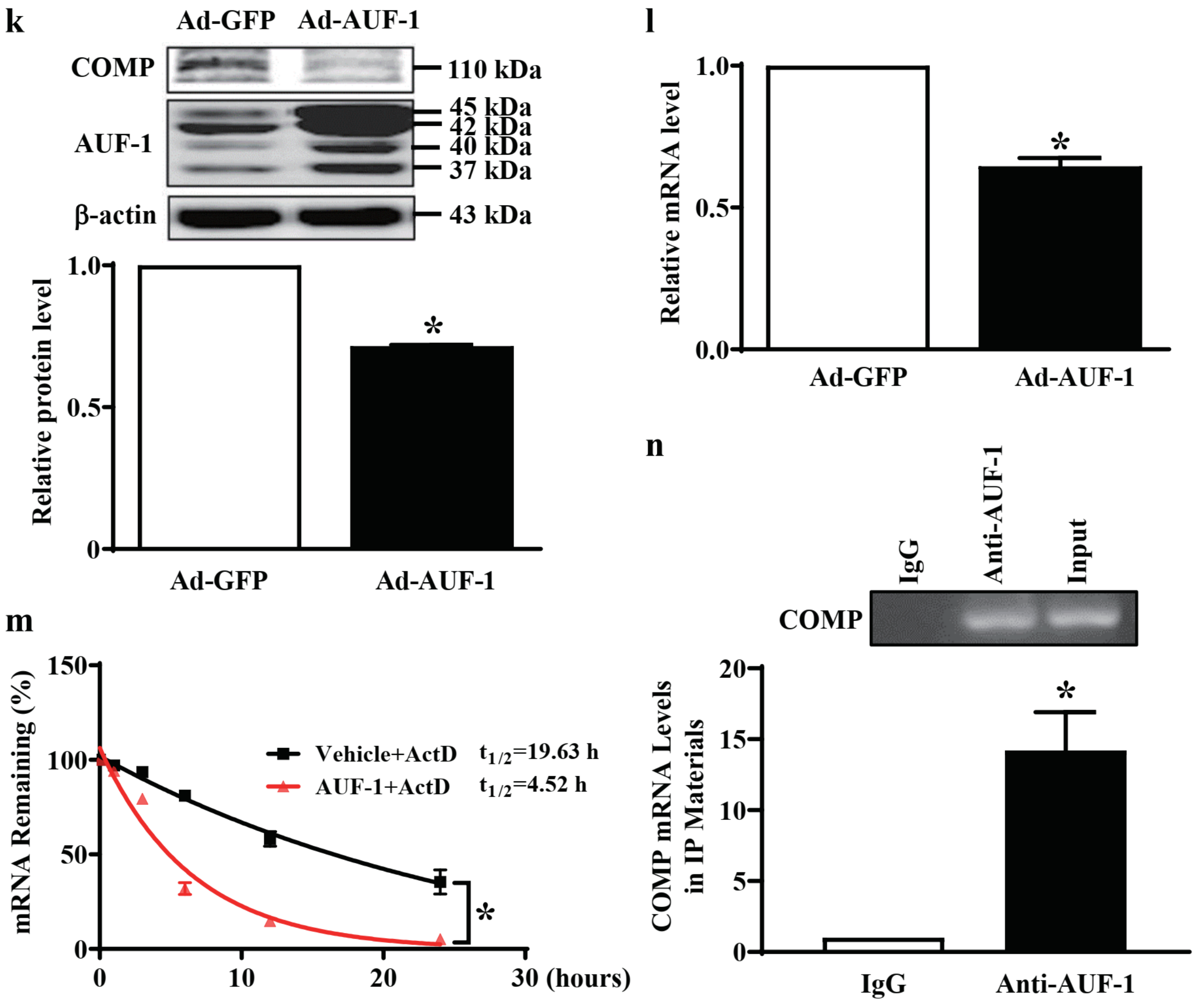

Fig. S6: k. Representative western blot and quantitative analysis of COMP and AUF-1 expression in rat VSMCs infected with GFP or AUF-1 adenovirus for 48 hours. n=6, **P*<0.05 in the paired Student's *t* test. **l.** Real-time PCR analysis of COMP expression in rat VSMCs infected with GFP or AUF-1 adenovirus for 24 hours. n=6, **P*<0.05 in paired Student's *t* test. **m.** The mRNA lifetime of COMP in rat VSMCs infected with GFP or AUF-1 adenovirus was evaluated by real-time PCR following pretreatment with actinomycin D to inhibit transcription. n=4, **P*<0.05 in the unpaired Student's *t*-test. **n.** RNA immunoprecipitation assay in rat VSMCs. n=3, **P*<0.05 in the paired Student's *t*-test.
