# Supplementary material for: Cartilage oligomeric matrix protein is an endogenous β-arrestin-2-selective allosteric modulator of AT1 receptor counteracting vascular injury
Source: Cell Res. 2021 Jan 28;31(7):773–90. doi: 10.1038/s41422-020-00464-8 (PMC8249609; doi:10.1038/s41422-020-00464-8)
Supplement: Supplementary file 17 — Supplementary information, Figure S7 [file 41422_2020_464_MOESM17_ESM.pdf]

Supplementary Information, Figure S7

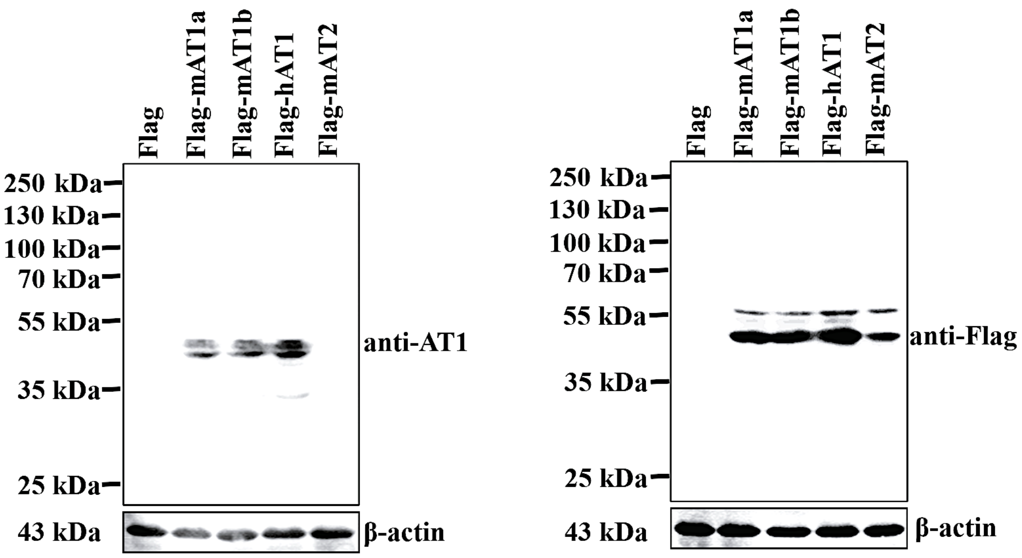

Fig. S7: Western blot analysis using anti-AT1 antibody (Left panel, dilution: 1:1000, exposure time using Odyssey Fc system: 30 sec.) or anti-Flag antibody (Right panel, dilution: 1:1000, exposure time using Odyssey Fc system: 1 min.) in HEK293A cells transfected with Flag-mouse AT1a, Flag-mouse AT1b, Flag-human AT1 and Flag-mouse AT2 plasmids.
