# Supplementary material for: Cartilage oligomeric matrix protein is an endogenous β-arrestin-2-selective allosteric modulator of AT1 receptor counteracting vascular injury
Source: Cell Res. 2021 Jan 28;31(7):773–90. doi: 10.1038/s41422-020-00464-8 (PMC8249609; doi:10.1038/s41422-020-00464-8)
Supplement: Supplementary file 18 — Supplementary information, Figure S8 [file 41422_2020_464_MOESM18_ESM.pdf]

Supplementary Information, Figure S8

a

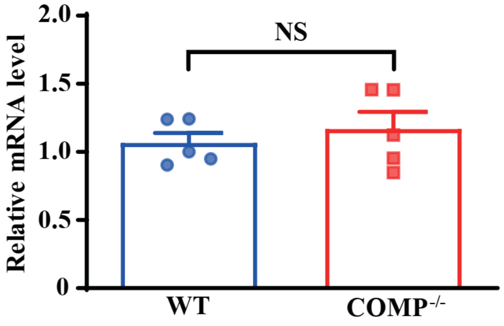

b

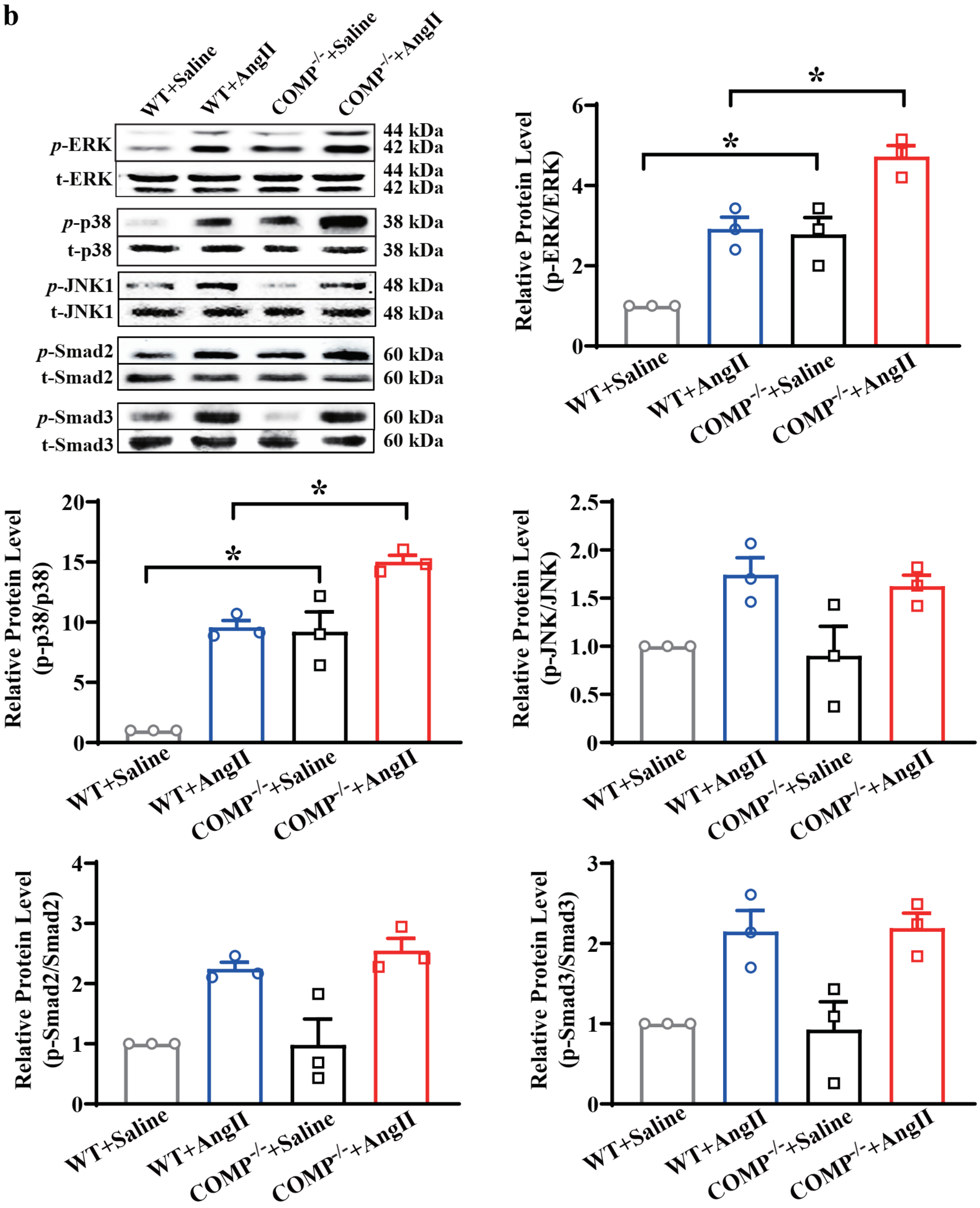

Fig. S8: a. Level of the AT1a mRNA in the mouse suprarenal aortas. Suprarenal aortas isolated from WT or *COMP*$^{-/-}$ mice. n=5 mice per group, NS, no significance in the unpaired Student's *t*-test. **b.** Western blot analysis of signaling molecules (ERK1/2, p38, JNK1, Smad2 and Smad3) in the suprarenal aortas of WT and *COMP*$^{-/-}$ mice infused with saline or AngII for 7 days. n=3, *$P<0.05$ in One-way ANOVA followed by the Bonferroni test.
