# Supplementary material for: Cartilage oligomeric matrix protein is an endogenous β-arrestin-2-selective allosteric modulator of AT1 receptor counteracting vascular injury
Source: Cell Res. 2021 Jan 28;31(7):773–90. doi: 10.1038/s41422-020-00464-8 (PMC8249609; doi:10.1038/s41422-020-00464-8)
Supplement: Supplementary file 19 — Supplementary information, Figure S9 [file 41422_2020_464_MOESM19_ESM.pdf]

Supplementary Information, Figure S9

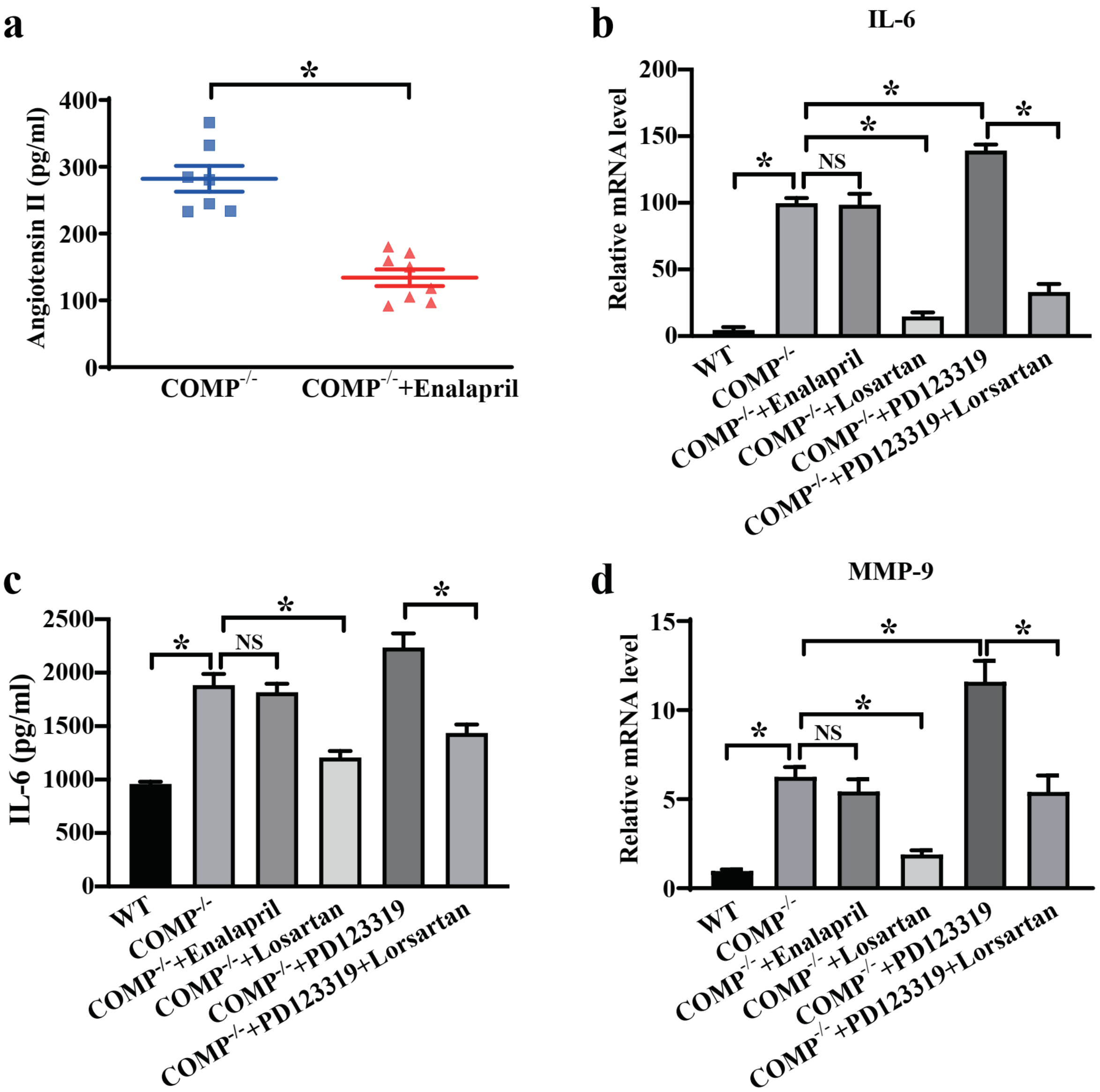

Fig. S9: a. AngII concentrations in aortic explants isolated from *COMP*$^{-/-}$ mice with or without administration of drinking water containing enalapril (15 mg/kg/day) for 7 days. n=7-8 mice per group, **P*<0.05 in Mann-Whitney test. **b.** Level of the IL-6 mRNA in the mouse suprarenal aorta. Suprarenal aortas isolated from WT or *COMP*$^{-/-}$ mice administered drinking water containing losartan (30 mg/kg/day), PD123319 (30 mg/kg/day) or enalapril (15 mg/kg/day) for 7 days. n=6-8 mice per group, Kruskal-Wallis test followed by Dunn's test, **P*<0.05, NS, no significance. **c.** IL-6 secretion into the conditioned medium from suprarenal aorta organ cultures. Suprarenal aortas were isolated from WT or *COMP*$^{-/-}$ mice or *COMP*$^{-/-}$ mice administered drinking water containing losartan, PD123319 or enalapril. n=6-8 mice per group, Kruskal-Wallis test followed by Dunn's test, **P*<0.05. NS, no significance. **d.** Level of the MMP-9 mRNA in the *ex vivo* cultured suprarenal aortas of 5-month-old *COMP*$^{-/-}$ mice administered drinking water containing losartan, PD123319 or enalapril for 7 days. n=6-8 mice per group, Kruskal-Wallis test followed by Dunn's test, **P*<0.05, NS, no significance.
