# Supplementary material for: Cartilage oligomeric matrix protein is an endogenous β-arrestin-2-selective allosteric modulator of AT1 receptor counteracting vascular injury
Source: Cell Res. 2021 Jan 28;31(7):773–90. doi: 10.1038/s41422-020-00464-8 (PMC8249609; doi:10.1038/s41422-020-00464-8)
Supplement: Supplementary file 20 — Supplementary information, Figure S10 [file 41422_2020_464_MOESM20_ESM.pdf]

Supplementary Information, Figure S10

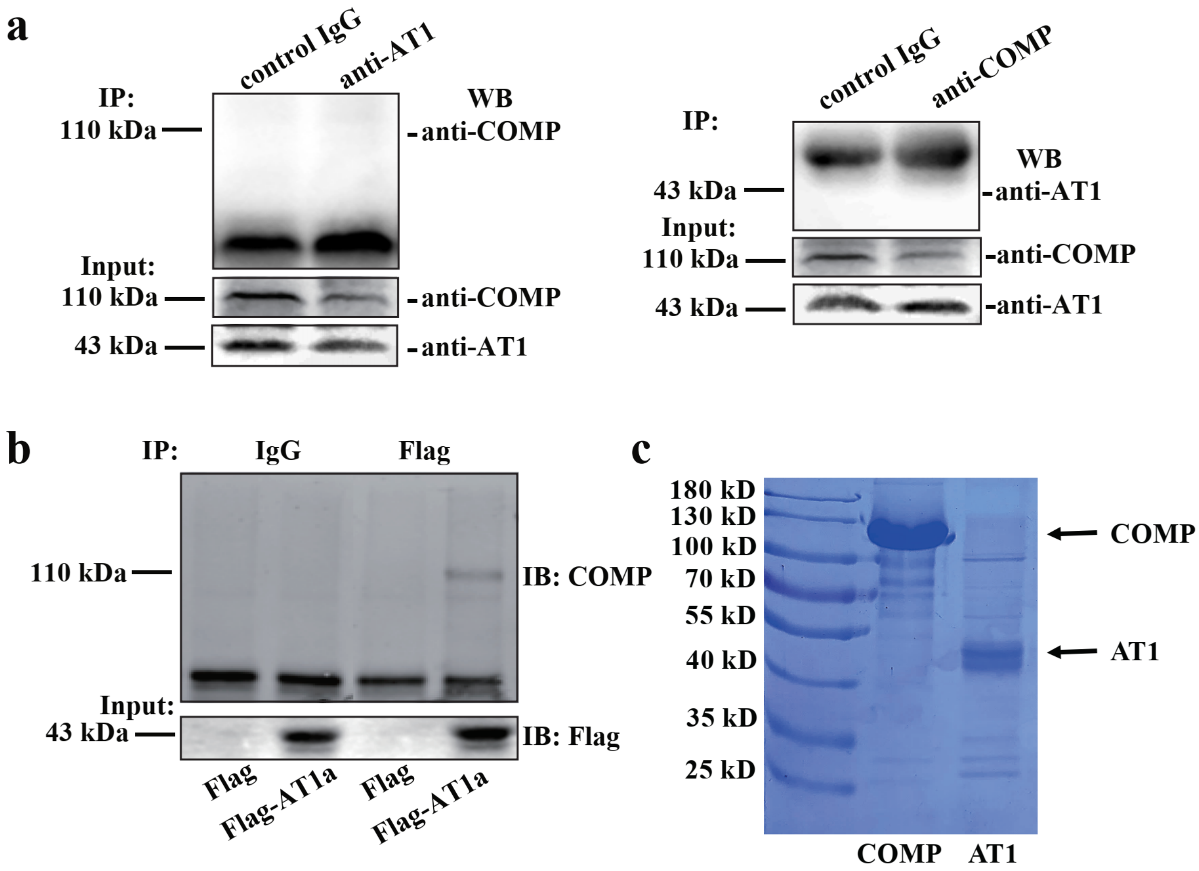

Fig. S10: a. Co-IP assay of suprarenal aortas from AT1a$^{-/-}$ mice. Left panel, Vascular extracts were incubated with an anti-AT1 antibody or control IgG, followed by Protein A/G agarose beads. The COMP protein was examined using a western blot analysis. Right panel, Vascular extracts were immunoprecipitated with an anti-COMP antibody or control IgG. The AT1 protein was then examined using a western blot analysis. Input was evaluated using aortic lysates before immunoprecipitation. **b.** The cDNA encoding the mouse AT1a receptor (NCBI reference sequence: NM_177322.3) was subcloned in-frame into the flag vector to generate the Flag-AT1a plasmid. Co-IP of COS-7 cells transfected with these plasmids, followed by incubation with purified COMP (5 μg/ml) for 30 min. Cell lysates were incubated with an anti-flag antibody or control IgG followed by Protein A/G agarose beads, and the bound COMP

protein was examined using a western blot analysis. **c.** Coomassie Brilliant Blue G250 staining of COMP and AT1 protein purified through baculovirus-infected insect cells.

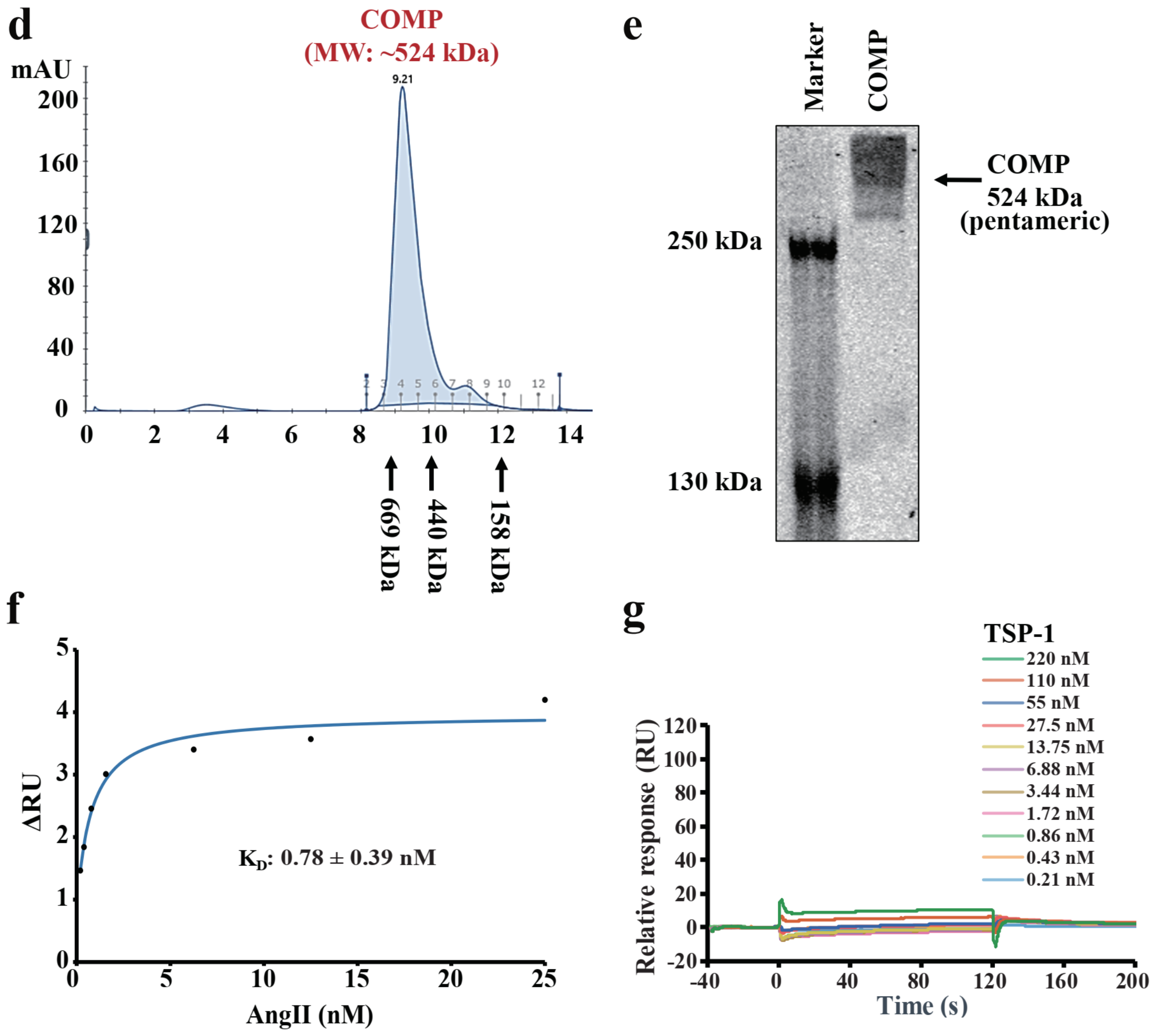

Fig. S10: d. The gel filtration chromatography analysis of purified COMP protein. **e.** Non-reduced western blot analysis of purified COMP protein by using native PAGE gels. **f.** The saturation curve of the binding of an increasing amount of AngII to AT1 receptor captured on a CM5 chip, acquired from the SPR data. **g.** SPR sensorgrams of the binding of an increasing amount of TSP-1 to AT1 receptor captured on a CM5 chip.
