# Supplementary material for: Cartilage oligomeric matrix protein is an endogenous β-arrestin-2-selective allosteric modulator of AT1 receptor counteracting vascular injury
Source: Cell Res. 2021 Jan 28;31(7):773–90. doi: 10.1038/s41422-020-00464-8 (PMC8249609; doi:10.1038/s41422-020-00464-8)
Supplement: Supplementary file 21 — Supplementary information, Figure S11 [file 41422_2020_464_MOESM21_ESM.pdf]

Supplementary Information, Figure S11

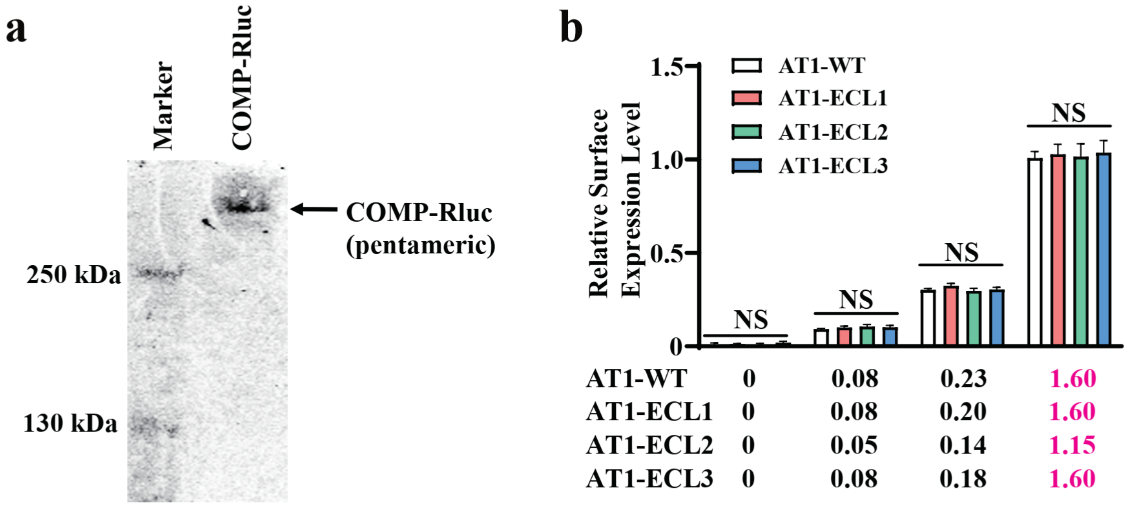

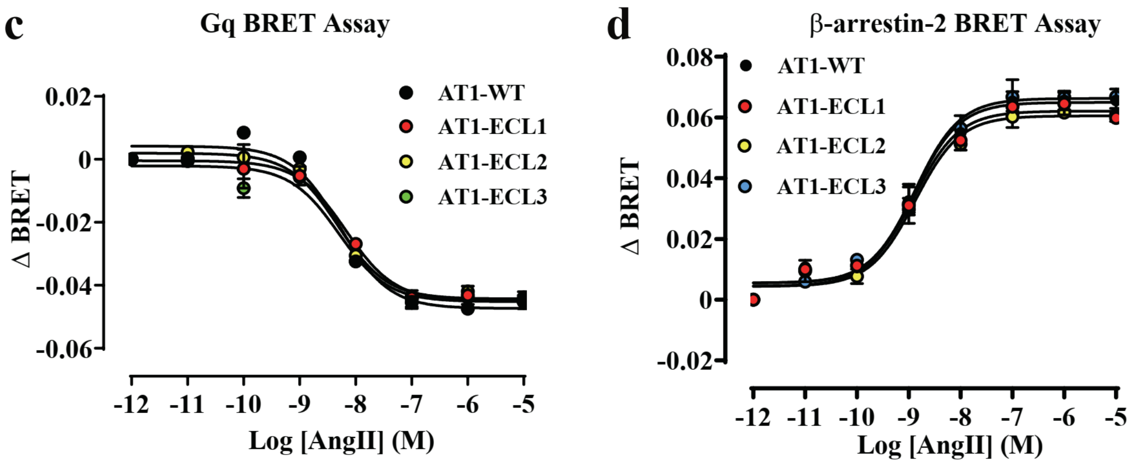

	EC50 (nM)	Emax (%)
AT1R-WT	6.02 ± 0.90	100
AT1R-ECL1	6.87 ± 1.60	99.34 ± 7.01
AT1R-ECL2	6.28 ± 1.58	97.34 ± 4.20
AT1R-ECL3	6.24 ± 1.83	100.1 ± 8.97

	EC50 (nM)	Emax (%)
AT1R-WT	1.49 ± 0.11	100
AT1R-ECL1	1.42 ± 0.09	96.11 ± 0.86
AT1R-ECL2	1.33 ± 0.02	99.35 ± 4.57
AT1R-ECL3	1.41 ± 0.07	105.2 ± 2.23

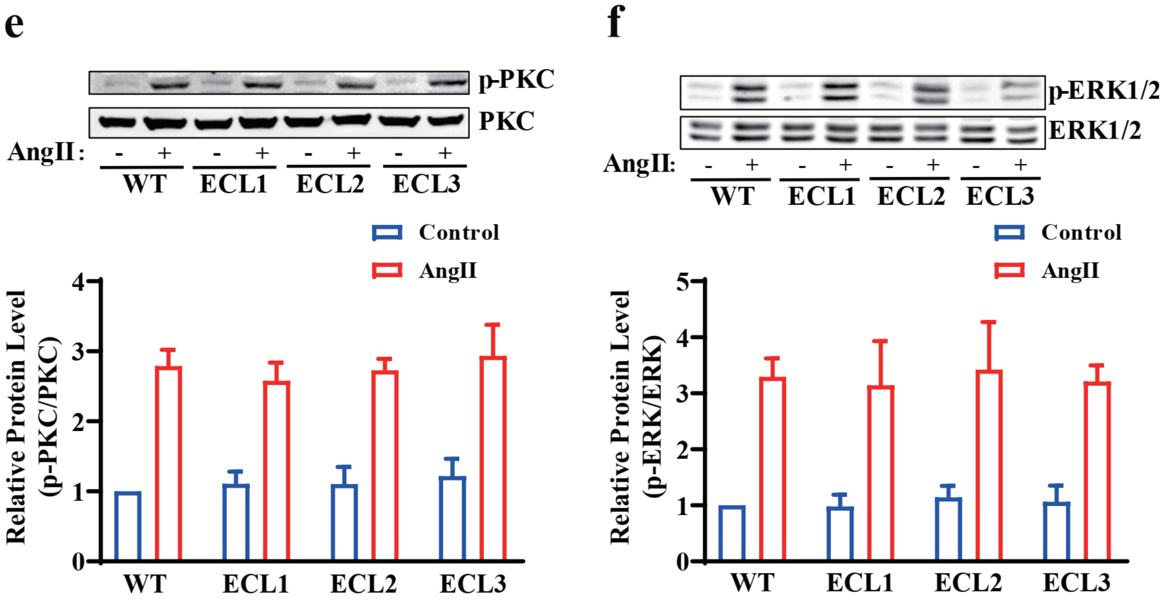

Fig. S11: a. Non-reduced western blot analysis of pentameric COMP (~524 kDa) produced by HEK293T cells overexpressing COMP-Rluc fusion protein by using native PAGE gels. **b.** The whole-cell ELISA measuring the cell surface expression of AT1 receptor in HEK293T transfected with various amounts (μg per 5×10^5 cells) of plasmids encoding the Flag-AT1 with or without the insertion of FlAsH motif at ECL1, ECL2 or ECL3. n=3; NS, no significance in One-way ANOVA followed by the Bonferroni test. The amounts highlighted in red text were applied for further experiments. **c.** The Gq activation BRET assay using HEK293T cells overexpressing the various chimeric AT1 receptors in response to an increasing amount of AngII stimulation for 2 min. n=3; NS, no significance in One-way ANOVA followed by the Bonferroni test. **d.** The β-arrestin-2 recruitment BRET assay using HEK293T cells overexpressing the various chimeric AT1 receptors in response to an increasing amount of AngII stimulation for 5 min. n=3; NS, no significance in One-way ANOVA followed by the Bonferroni test. **e-f.** Western blot analysis of p-PKC/pan-PKC (**e**) and p-ERK/ERK (**f**) levels in AngII-induced HEK293A cells overexpressing Flag-AT1 FlAsH-BRET sensors. n=3, One-way ANOVA followed by the Bonferroni test.

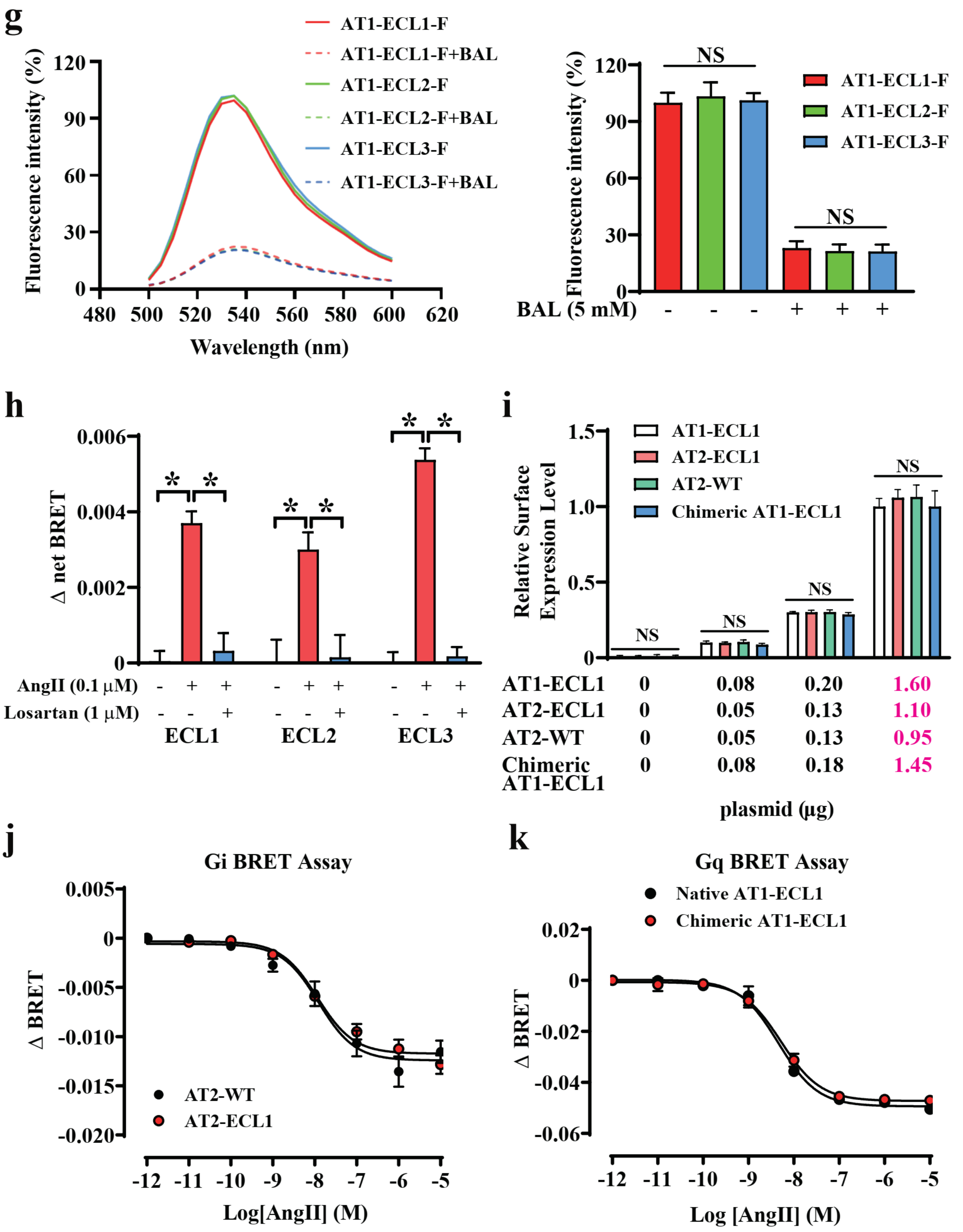

	EC50 (nM)	Emax (%)
AT2-WT	10.26 ± 1.07	100
AT2-ECL1	9.79 ± 1.27	98.47 ± 1.52

	EC50 (nM)	Emax (%)
Native AT1-ECL1	5.93 ± 1.23	100
Chimeric AT1-ECL1	6.47 ± 1.49	98.10 ± 2.06

Fig. S11: g. Left panel: Fluorescence emission spectrum of AT1-ECL-FlAsH after labelling. HEK293T cells overexpressing AT1-ECL-FlAsH were labelled with FlAsH-EDT2 and the membrane fractions were isolated and treated with or without 5 mM BAL (2,3-dimercaptopropanol) before fluorescence was measured with 460 nm excitation. Right panel: Quantification of fluorescence of AT1-ECL-FlAsH after labelling. Data were normalized to the fluorescence of AT1-ECL1-FlAsH without BAL treatment. n=3, NS, no significance in One-way ANOVA followed by the Bonferroni test. **h.** BRET signal of three AT1-ECL-FlAsH mutants with RLuc fused at N-terminus in response to AngII stimulation. n=3, $^*P<0.05$ in One-way ANOVA followed by the Bonferroni test. **i.** The whole-cell ELISA measuring the cell surface expression of AT1 or AT2 receptor in HEK293T transfected with various amounts (μg per 5×10^5 cells) of plasmids encoding the Flag-AT1 or Flag-AT2 with or without the insertion of FlAsH motif at ECL1. n=3; NS, no significance in One-way ANOVA followed by the Bonferroni test. The amounts highlighted in red text were applied for further experiments. **j.** The Gi activation BRET assay using HEK293T cells overexpressing WT or mutant AT2 receptors in response to an increasing amount of AngII stimulation for 2 min. n=3; NS, no significance in One-way ANOVA followed by the Bonferroni test. **k.** The Gq activation BRET assay using HEK293T cells overexpressing the native or chimeric AT1-ECL-FlAsH in response to an increasing amount of AngII stimulation for 2 min. n=3; NS, no significance in One-way ANOVA followed by the Bonferroni test.
