# Supplementary material for: Cartilage oligomeric matrix protein is an endogenous β-arrestin-2-selective allosteric modulator of AT1 receptor counteracting vascular injury
Source: Cell Res. 2021 Jan 28;31(7):773–90. doi: 10.1038/s41422-020-00464-8 (PMC8249609; doi:10.1038/s41422-020-00464-8)
Supplement: Supplementary file 22 — Supplementary information, Figure S12 [file 41422_2020_464_MOESM22_ESM.pdf]

Supplementary Information, Figure S12

a

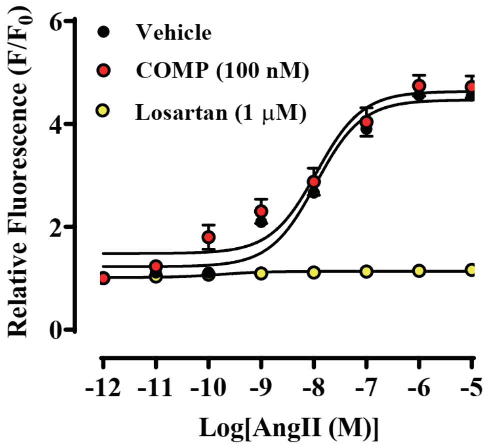

	EC50 (nM)	Emax (%)
**Vehicle**	11.09 ± 1.06	100
**COMP**	11.33 ± 2.14	103.74 ± 3.89
**Losartan**	ND	ND

b

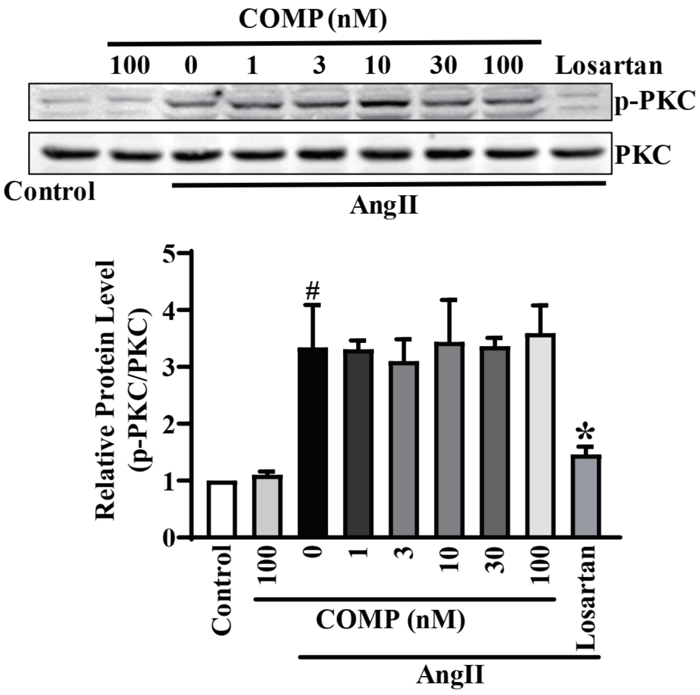

Fig. S12: a. HEK293A cells were transfected with Flag-AT1 receptor. The transfected cells were preincubated with purified COMP (100 nM) or losartan (1 μM), followed by the stimulation of an increasing amount of AngII for 2 min. The intracellular Ca^{2+} mobilization was measured using Fluo-4-AM. n=3, One-way ANOVA followed by the Bonferroni test. **b.** Representative Western blot analysis (upper panel) and quantification (lower panel) of p-PKC and pan-PKC levels in AngII-induced HEK293A cells overexpressing the human AT1 receptor

in the presence of various concentrations of purified COMP. n=3, Two-way ANOVA followed by the Bonferroni test, $^{\#}P$<0.05 *vs.* Control, *P<0.05 *vs.* AngII.
