# Supplementary material for: Cartilage oligomeric matrix protein is an endogenous β-arrestin-2-selective allosteric modulator of AT1 receptor counteracting vascular injury
Source: Cell Res. 2021 Jan 28;31(7):773–90. doi: 10.1038/s41422-020-00464-8 (PMC8249609; doi:10.1038/s41422-020-00464-8)
Supplement: Supplementary file 23 — Supplementary information, Figure S13 [file 41422_2020_464_MOESM23_ESM.pdf]

Supplementary Information, Figure S13

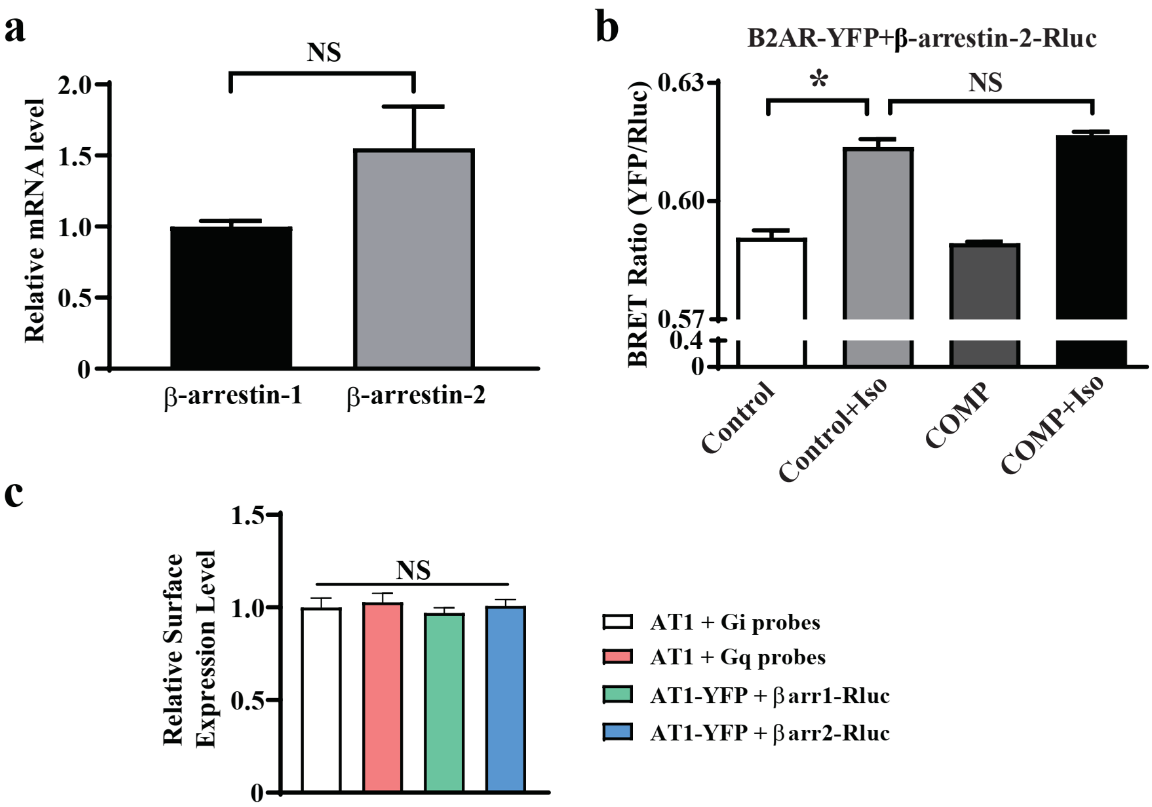

Fig. S13: a. Real-time PCR for measuring the expression level of β-arrestin-1 and -2 in suprarenal aortas from C57 mice. n=5, NS, no significance in the unpaired Student's *t*-test. **b.** Effects of COMP on isoprenaline-induced β-arrestin-2 recruitment through B2AR. HEK293T cells overexpressing B2AR-YFP and β-arrestin-2-RLuc were pre-incubatedwith purified COMP (5 μg/mL). Then, the BERT signal was measure after isoprenaline (Iso, 10 nM) was applied for 5 min. n=3, Two-way ANOVA followed by the Bonferroni test, *$P<0.05$, NS, no significance. **c.** The whole-cell ELISA measuring the cell surface expression of AT1 receptor in HEK293T cells (5×10^5 cells) transfected with 2 μg of AT1 plasmids, including Flag-AT1 and Flag-AT1-YFP applied in Gq/Gi BRET asaay and β-arrestin-1/β-arrestin-2 BRET assay, respectively. n=3; NS, no significance in One-way ANOVA followed by the Bonferroni test.

d

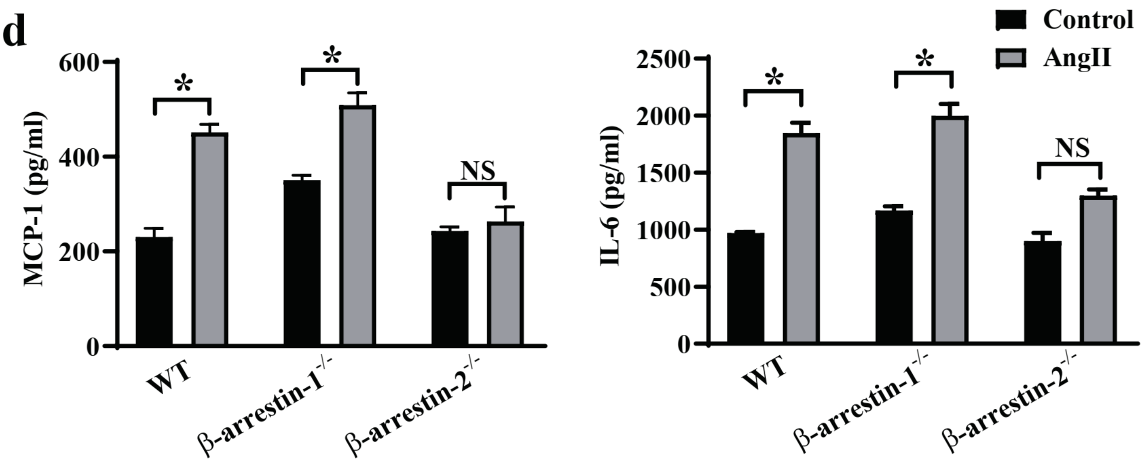

e

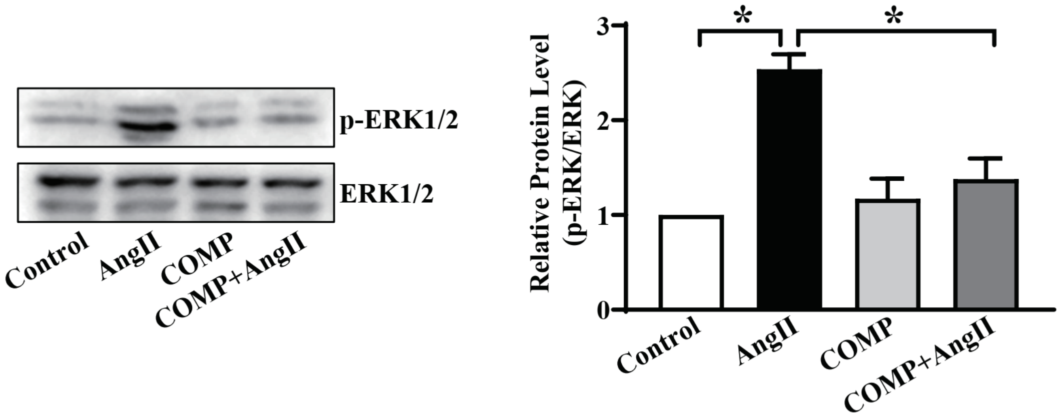

Fig. S13: d. ELISA measurements of MCP-1 and IL-6 in conditional media of suprarenal aortic rings from WT, *β-arrestin-1*$^{-/-}$ or *β-arrestin-2*$^{-/-}$ mice in absence or presence of AngII (1 μM) treatment for 48 hours. n=5, *$P<0.05$ in Two-way ANOVA followed by the Bonferroni test. **e.** HEK293A cells were transfected with Flag-AT1 receptor. The transfected cells were preincubated with purified COMP (100 nM), followed by the stimulation of the AngII (0.1 μM) for 15 min. Western blot analysis of p-ERK1/2 and total ERK1/2 expression. n=3, *$P<0.05$ in One-way ANOVA followed by the Bonferroni test.
