# Supplementary material for: Cartilage oligomeric matrix protein is an endogenous β-arrestin-2-selective allosteric modulator of AT1 receptor counteracting vascular injury
Source: Cell Res. 2021 Jan 28;31(7):773–90. doi: 10.1038/s41422-020-00464-8 (PMC8249609; doi:10.1038/s41422-020-00464-8)
Supplement: Supplementary file 24 — Supplementary information, Figure S14 [file 41422_2020_464_MOESM24_ESM.pdf]

Supplementary Information, Figure S14

a

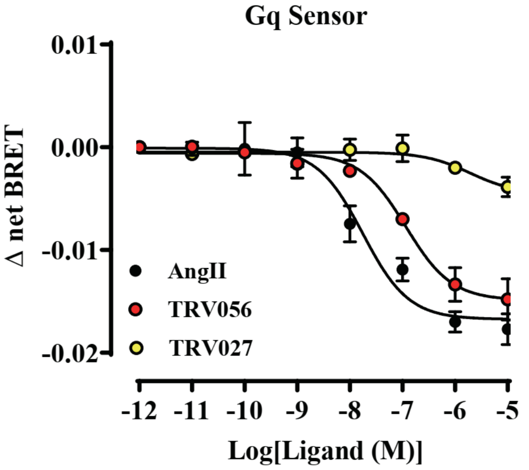

	EC50 (nM)	Emax (%)
**AngII**	14.43 ± 1.71	100
**TRV056**	112.6 ± 1.90	84.68 ± 0.83
**TRV027**	ND	ND

b

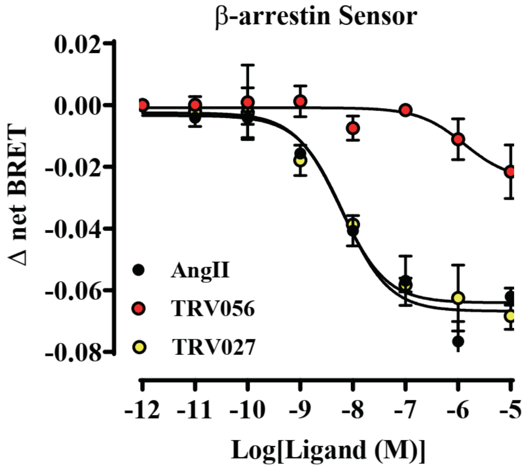

	EC50 (nM)	Emax (%)
**AngII**	7.10 ± 0.44	100
**TRV056**	ND	ND
**TRV027**	6.73 ± 0.53	100.7 ± 1.90

c

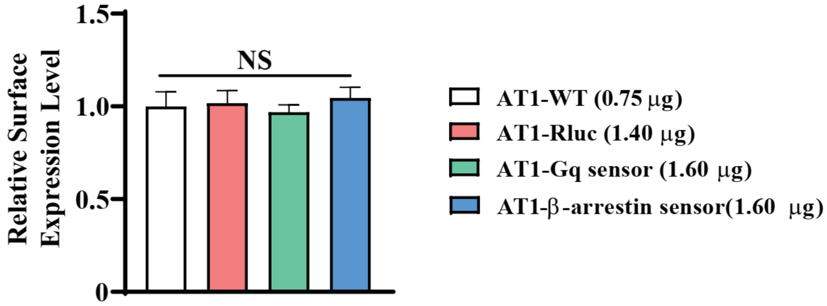

d

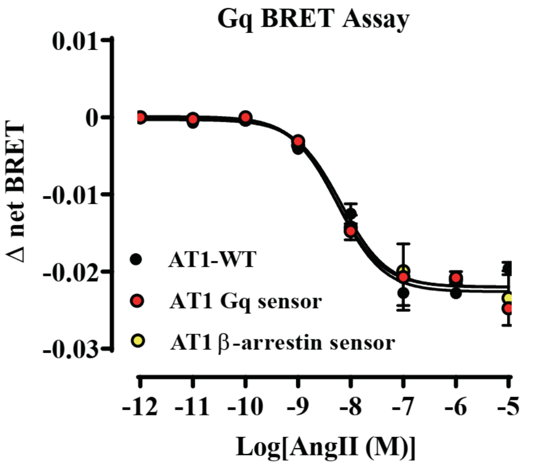

	EC50 (nM)	Emax (%)
**AT1-WT**	6.34 ± 0.40	100
**AT1-Gq sensor**	6.56 ± 0.48	99.75 ± 2.50
**AT1-Arr sensor**	6.32 ± 0.43	98.29 ± 1.30

e

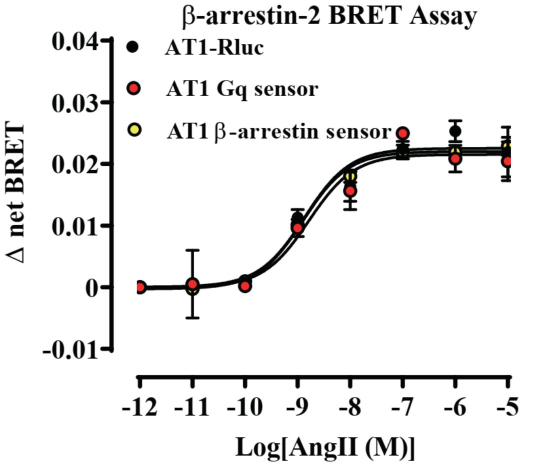

	EC50 (nM)	Emax (%)
**AT1-WT**	1.44 ± 0.07	100
**AT1-Gq sensor**	1.41 ± 0.16	101.0 ± 2.86
**AT1-Arr sensor**	1.43 ± 0.09	102.1 ± 2.62

Fig. S14: a. The FlAsH-BRET measurements in HEK293T cells transfected with the Gq-specific conformational sensor of AT1 receptor. Transfected cells were stimulated with an increasing amount of AngII, TRV056 (a Gq-selective agonist) and TRV026 (a β-arrestin-selective agonist). n=3, One-way ANOVA followed by the Bonferroni test. **b.** The FlAsH-BRET measurements in HEK293T cells transfected with the β-arrestin-specific conformational sensor of AT1 receptor. Transfected cells were stimulated with an increasing amount of AngII, TRV056 (a Gq-selective agonist) and TRV026 (a β-arrestin-selective agonist). n=3, One-way ANOVA followed by the Bonferroni test. **c.** The whole-cell ELISA measuring the cell surface expression of AT1 receptor in HEK293T transfected with wide-type or mutant AT1 plasmids. n=3; NS, no significance in One-way ANOVA followed by the Bonferroni test. **d.** The Gq activation BRET assay using HEK293T cells overexpressing the WT AT1 or AT1 conformational sensors in response to an increasing amount of AngII stimulation for 2 min. n=3; NS, no significance in One-way ANOVA followed by the Bonferroni test. **e.** The β-arrestin-2 activation BRET assay using HEK293T cells overexpressing the AT1-RLuc or AT1 conformational sensors in response to an increasing amount of AngII stimulation for 5 min. n=3; NS, no significance in One-way ANOVA followed by the Bonferroni test.
