# Supplementary material for: Cartilage oligomeric matrix protein is an endogenous β-arrestin-2-selective allosteric modulator of AT1 receptor counteracting vascular injury
Source: Cell Res. 2021 Jan 28;31(7):773–90. doi: 10.1038/s41422-020-00464-8 (PMC8249609; doi:10.1038/s41422-020-00464-8)
Supplement: Supplementary file 25 — Supplementary information, Figure S15 [file 41422_2020_464_MOESM25_ESM.pdf]

Supplementary Information, Figure S15

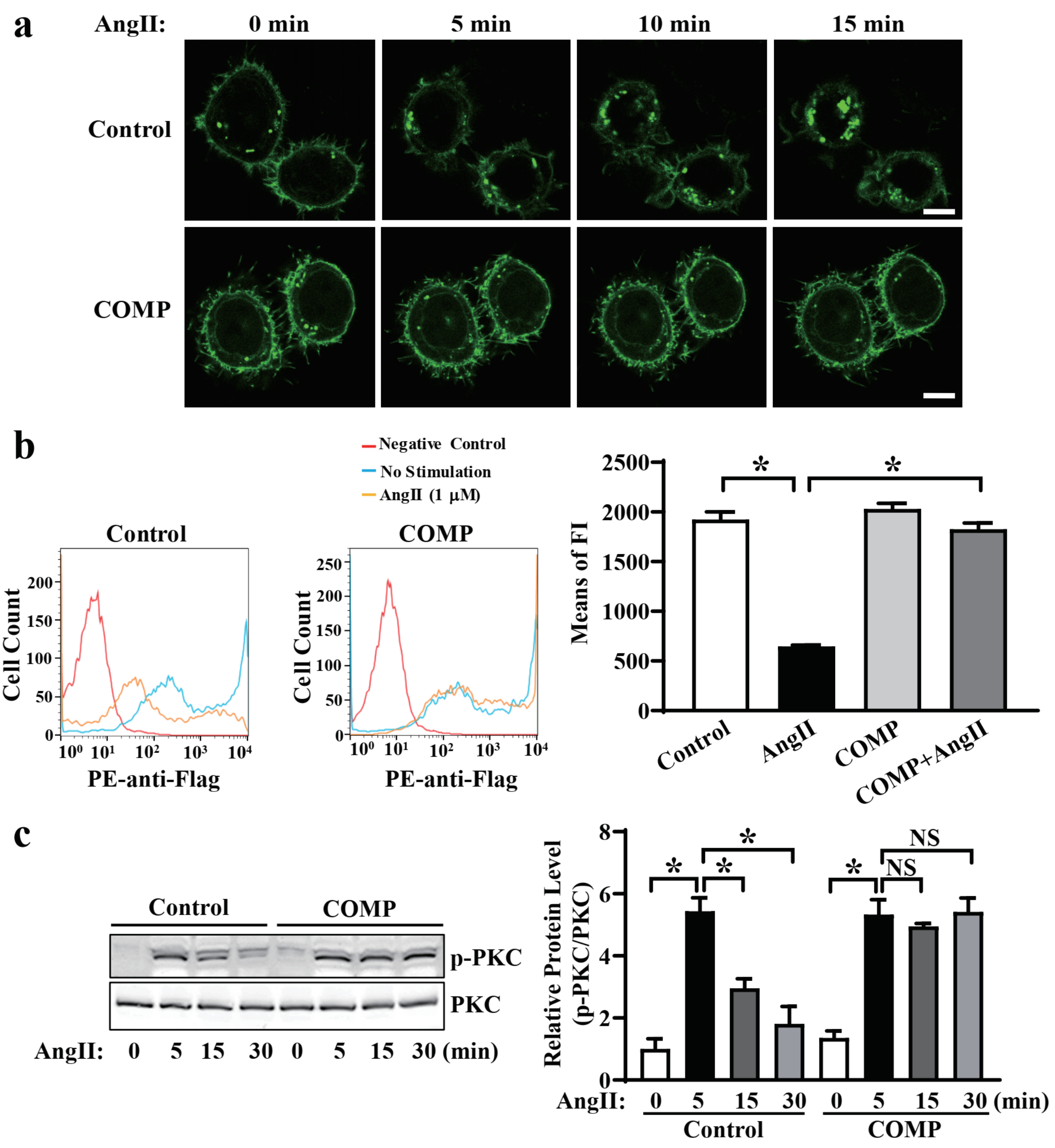

Fig. S15: a. Hela cells transfected with AT1-GFP plasmid were pretreated with COMP (5 μg/ml) for 30 min. Following stimulation with AngII (1 μM) for 15 min, receptor internalization was observed under a confocal laser scanning microscope at various time points. Scale bar, 10 μm. **b.** HEK293A cells transfected with AT1-Flag plasmid (with a Flag-tag at N terminus) were pretreated with COMP (5 μg/ml) for 30 min. Following stimulation with AngII

(1 μM) for 15 min, flow cytometry analysis of the cell surface AT1 location in the cells by PE-anti-Flag antibodies, whereas mouse IgG1 was applied as a negative control of labeling antibodies. The means of fluorescent intensity (FI) in each sample were utilized for data quantification. n=4. $*P<0.05$ in Two-way ANOVA followed by the Bonferroni test. **c.** Representative Western blot analysis (left panel) and quantification (right panel) of p-PKC and pan-PKC levels in HEK293A cells overexpressing the human AT1 receptor, in the absence or presence of various concentrations of purified COMP, induced by AngII (1 μM) for various time points. n=3, Two-way ANOVA followed by the Bonferroni test, $*P<0.05$; NS, no significance.
