# Supplementary material for: Cartilage oligomeric matrix protein is an endogenous β-arrestin-2-selective allosteric modulator of AT1 receptor counteracting vascular injury
Source: Cell Res. 2021 Jan 28;31(7):773–90. doi: 10.1038/s41422-020-00464-8 (PMC8249609; doi:10.1038/s41422-020-00464-8)
Supplement: Supplementary file 26 — Supplementary information, Figure S16 [file 41422_2020_464_MOESM26_ESM.pdf]

**Supplementary Information, Figure S16**

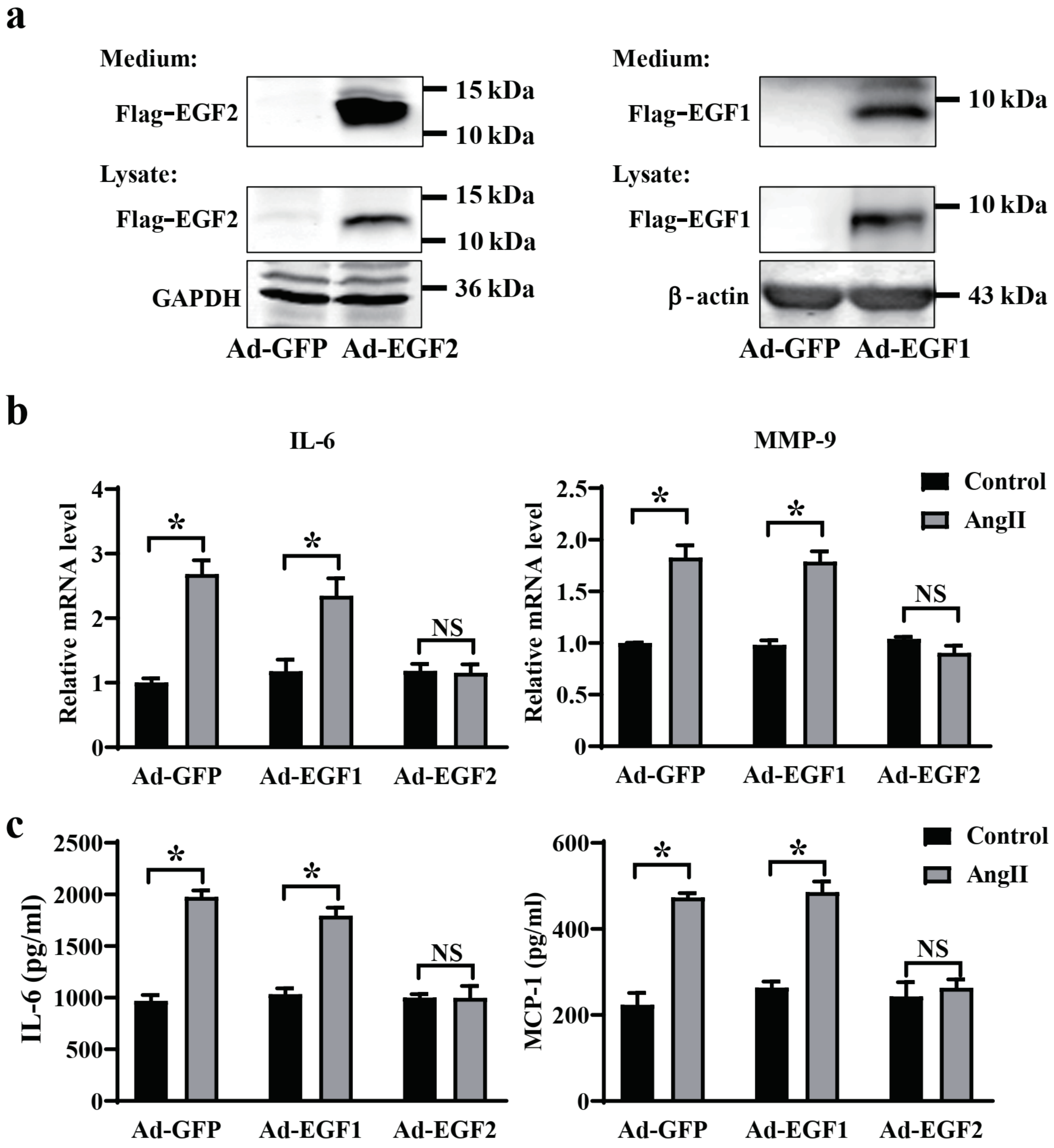


**Fig. S16: a.** Western blot analysis of COMP-EGF2 (53 aa) and COMP-EGF1 (39 aa) domain overexpressed in COS-7 cells through adenovirus infection (Ad-GFP: Control, Ad-EGF2: COMP-EGF2 and Ad-EGF1: COMP-EGF1) in both cell lysate and condensed conditional media. **b.** Real-time PCR analysis of IL-6 and MMP-9 expressions in suprarenal aortic rings from *ApoE*$^{-/-}$ mice infected with various adenovirus for 7 days, followed by the AngII (1 μM)

treatment for 48 hours. n=3, *$P$<0.05 in Two-way ANOVA followed by the Bonferroni test. **c.** ELISA measurements of IL-6 and MCP-1 in conditional media of suprarenal aortic rings from *ApoE*$^{-/-}$ mice infected with various adenovirus for 7 days, followed by the AngII (1 μM) treatment for 48 hours. n=3, *$P$<0.05 in Two-way ANOVA followed by the Bonferroni test.

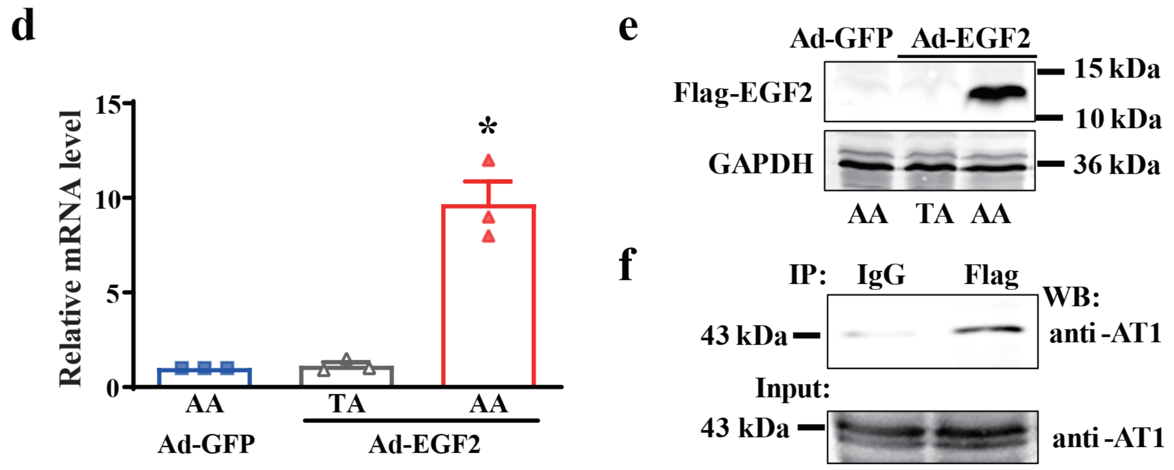


**Fig. S16: d-e.** Real-time PCR (**d**) and Western blot (**e**) to measure the overexpression of COMP EGF2 domain in thoracic (TA) or suprarenal (AA) aortas of 5-month-old male *ApoE*$^{-/-}$ mice 3 days after the periadventitial infection with Ad-GFP and Ad-EGF2. n=3, One-way ANOVA followed by the Bonferroni test, *$P$<0.05 *vs.* AA infected with Ad-GFP. **f.** Co-IP assay of suprarenal aortas from 5-month-old male *ApoE*$^{-/-}$ mice 3 days after the periadventitial infection with Ad-EGF2. Aortic extracts were immunoprecipitated with an anti-Flag antibody or control IgG. The AT1 protein was then examined using a western blot analysis. Input was evaluated using aortic lysates before immunoprecipitation.
