# Supplementary material for: Cartilage oligomeric matrix protein is an endogenous β-arrestin-2-selective allosteric modulator of AT1 receptor counteracting vascular injury
Source: Cell Res. 2021 Jan 28;31(7):773–90. doi: 10.1038/s41422-020-00464-8 (PMC8249609; doi:10.1038/s41422-020-00464-8)
Supplement: Supplementary file 27 — Supplementary information, Figure S17 [file 41422_2020_464_MOESM27_ESM.pdf]

Supplementary Information, Figure S17

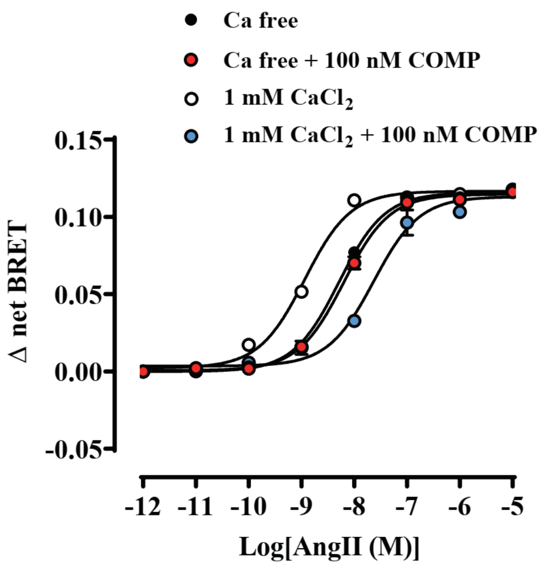

	EC50 (nM)	Emax (%)
**Ca free**	5.60 ± 0.25	100
**Ca free + COMP**	5.93 ± 0.46	102.2 ± 3.45
**1 mM $CaCl_2$**	1.06 ± 0.05#	104.5 ± 7.18
**1 mM $CaCl_2$ + COMP**	21.27 ± 1.52*	99.71 ± 8.69

Fig. S17: Effect of Ca^{2+} on the antagonistic function of COMP at AngII-induced β-arrestin2 recruitment to AT1 receptor. HEK293T cells overexpressing AT1-YFP and β-arrestin2-RLuc were pre-incubated with without COMP (100 nM), followed by stimulation with an increasing amount of AngII in Ca^{2+}-depleted (1 mM EGTA) and Ca^{2+}-containing (1 mM $CaCl_2$) system. BRET signal between YFP and RLuc was measured and calculated. n=3, One-way ANOVA followed by the Bonferroni test. *P<0.05 *vs.* 1 mM $CaCl_2$; #P<0.05 *vs.* Ca free.
